# Targeting a Glutamic Acid in PDEδ with Fluoromethyl-Aryl Electrophiles Impairs K‑Ras Signaling

**DOI:** 10.1021/acs.jmedchem.5c02082

**Published:** 2026-01-07

**Authors:** Ruirui Zhang, Maxim A. Huetzen, Aylin Binici, Pablo Martín-Gago, Raphael Gasper, Elena Rudashevskaya, Jie Liu, Chinta Nagaraju, Elena S. Reckzeh, Alana S. T. Stuedle, Ann-Sophie Hopff, Andrea Mesaros, Anke Unger, Melanie Thelen, Petra Janning, H. Christian Reinhardt, Slava Ziegler, Ron D. Jachimowicz, Herbert Waldmann

**Affiliations:** † Department of Chemical Biology, 28268 Max Planck Institute of Molecular Physiology, Otto-Hahn-Street 11, Dortmund 44227, Germany; ‡ Max Planck Research Group Mechanisms of DNA Repair, 130368 Max Planck Institute for Biology of Ageing, Cologne 50931, Germany; § Department I of Internal Medicine, Center for Integrated Oncology Aachen Bonn Cologne Duesseldorf (CIO ABCD), University of Cologne, Cologne 50931, Germany; ∥ Cologne Excellence Cluster on Cellular Stress Response in Aging-Associated Diseases, University of Cologne, Cologne 50931, Germany; ⊥ Center for Molecular Medicine Cologne, University of Cologne, Cologne 50931, Germany; # Faculty of Chemistry and Chemical Biology, 14311 Technical University Dortmund, Otto-Hahn-Street 6, Dortmund 44221, Germany; ¶ Crystallography and Biophysics Facility, Max Planck Institute of Molecular Physiology, Otto-Hahn-Street 11, Dortmund 44227, Germany; ∇ Phenotyping Core Facility, Max Planck Institute for Biology of Ageing, Cologne 50931, Germany; ○ Lead Discovery Center GmbH, Otto-Hahn-Str. 15, Dortmund D-44227, Germany; ⧫ Department of Hematology and Stem Cell Transplantation, University Hospital Essen, West German Cancer Center, German Cancer Consortium Partner Site Essen, Center for Molecular Biotechnology, University of Duisburg-Essen, Hufelandstraße 55, Essen 45147, Germany

## Abstract

For targeted covalent modification at low-reactivity carboxylates with biocompatible electrophiles, new approaches are in high demand. Engineering of the HaloTag protein facilitates such a covalent reaction between chloroalkanes and an aspartate residue. We demonstrate that conversely, engineering stable ligands can also enable covalent targeting of an acid residue in a protein binding site. Using the chaperone PDEδ, which shuttles lipidated oncoproteins and thereby mediates their signaling activity, we show that equipping noncovalent inhibitors with a benzyl fluoride-based electrophile leads to covalent modification of a specific glutamate p.E88 in the ligand binding site. The best inhibitor, Deltafluorine, embodies a 3-fluoromethyl-pyridyl group and is stable to nucleophiles like glutathione, phosphate, acetate, and citrate. In cells, Deltafluorine combines noncovalent and covalent reactivity to demonstrate distinct cellular profiles and inhibits signaling through the MAP-kinase and Akt-mTOR pathways. In an autochthonous mouse model of highly aggressive Kras^G12D^-driven lung adenocarcinoma, Deltafluorine treatment significantly reduces tumor volume.

## Graphical abstract

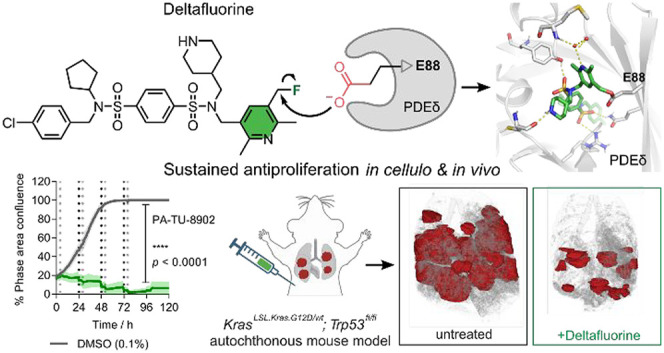

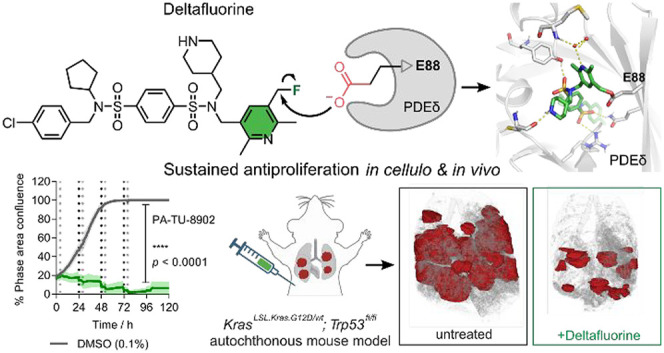

## Introduction

Small molecules equipped with electrophilic reactive groups are invaluable tools for the study of protein function and hold significant therapeutic potential.^1–3^ Covalent targeting offers a reactivity-driven strategy that is particularly effective for the selective modification of specific residues.^1–3^ Among nucleophilic amino acid residues suitable for covalent strategies, carboxylates (Glu, E and Asp, D) are especially notable, as these residues are abundant in the proteome (approximately 12%). They are essential for numerous biochemical processes, functionally diverse, and often display heightened or aberrant activity in key drug targets,^4–6^ which makes them appealing sites for targeted covalent ligand discovery.^7,8^ However, due to their low intrinsic nucleophilicity, these residues in general are targeted with reactive electrophiles, which may have limited stability in physiologically relevant media and be prone to also covalently react with more nucleophilic amino acids, in particular Cys and Lys, such that Glu and Asp residues have thus far been targeted covalently in relatively few instances.^5,6,9–19^ Such undesired off-target reactivity and limited stability might be overcome if carboxylates in binding sites could be targeted with low reactivity electrophiles, and new approaches to achieve this goal are in high demand.

We recently developed a covalent glutamic acid targeting strategy utilizing biocompatible and selective warheads inspired by the HaloTag technology.^20^ The HaloTag system employs a covalent conjugation reaction between ligands with a reactive chloroalkane linker and aspartic acid D106 in the HaloTag protein, forming a stable ester construct (Figure 1a).^21^ In parallel, covalent reactions were achieved in lipoprotein binding chaperone phosphodiesterase of retinal rod delta subunit (PDEδ) with alkyl bromides derived from reversible inhibitors to target its binding site glutamate p.E88.^20^ In contrast to the HaloTag system, however, we observed that the corresponding alkyl chlorides derived from the same reversible PDEδ inhibitor failed to react with p.E88 in PDEδ.^20^ The reactivity of alkyl chlorides for covalency in the HaloTag system is achieved through generations of protein engineering, during which key amino acid residues were selected for site-specific mutagenesis to optimize the binding site and disposition of D106 for labeling kinetics and duration (Figure 1a).^21–25^ We reasoned that, alternatively, the precise position and orientation of the warhead toward the selected residue for covalency would also be possible from a classical ligand-directed TCI approach. We envisioned that with structural fine-tuning of small molecule ligands, targeted covalent binding can be achieved with innately low-reactivity warheads, such as alkyl fluoride-based electrophiles, which may offer reduced unspecific activity for covalent compounds (Figure 1b).^26^

**Figure 1:**
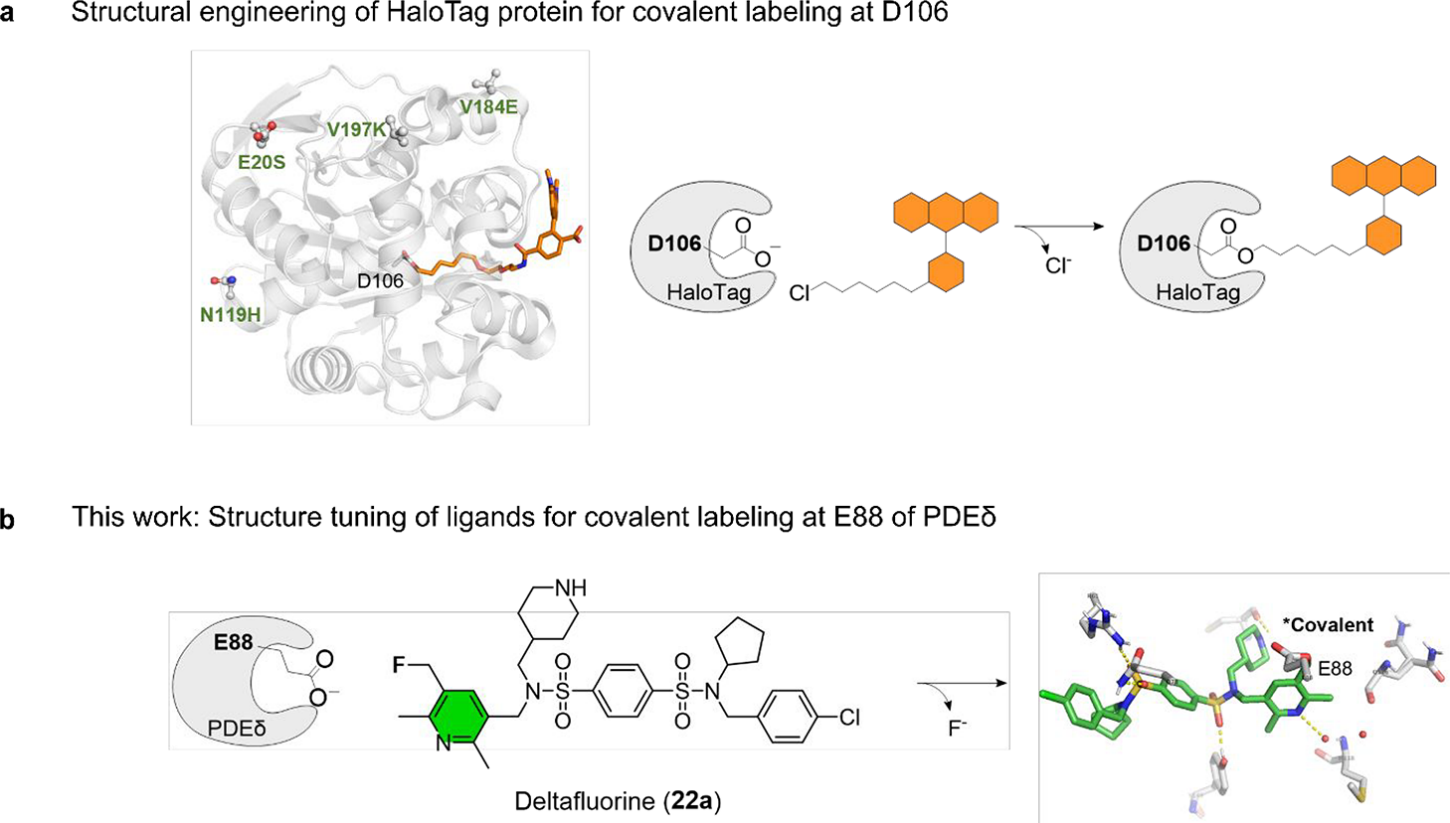
Concept for structural tuning to enable covalent targeting of carboxylate residues. (a) Structural engineering of HaloTag protein to optimize covalent binding between chloroalkane ligands and aspartate D106. Crystal structure of a TMR ligand bound to HaloTag protein (PDB code: 6Y7A) is shown.^52^ Beneficial mutation sites (green labels, specifically E20S, N119H, V184E, and V197 K mutations) that improve labeling speed are highlighted with ball-and-stick representations of side chains.^25^ (b) This work: structural tuning of small molecule ligands to achieve covalency with a pyridylmethyl fluoride-based warhead. Crystal structure of the covalent adduct formed from Deltafluorine (**22a**) and PDEδ (this work; PDB code: 9RP7) is shown with key interactions in the PDEδ prenyl binding pocket (hydrogen bond as yellow dotted line, red spheres as bound water molecules). Structural development of Deltafluorine (**22a**) was guided by cocrystal structures of previously developed inhibitors with PDEδ (PDB codes: 4JVF, 5E80, 5ML3, 5NAL, and 9HMD,^19,20,33–35^ see Figure S1 for details).

The intrinsic reactivity of fluoride motifs to nucleophiles was expected to be low, with poor leaving group properties of fluoride and the inherent stability of the C–F bond.^27,28^ However, in a few instances, fluoroalkyl groups have been employed to covalently target cysteines in cells.^29,30^ Additionally, the reactivity of alkyl halides in the α-position to carbonyl groups can be significantly enhanced, enabling α-fluoromethyl ketones to be employed as protease or kinase probes with demonstrated reactivity toward cysteines, and typically catalytic serines and threonines.^12,31,32^ The enhancement of the halide reactivity also applies to the benzylic position, albeit to a lesser extent. We therefore focused on covalent ligands incorporating benzyl fluoride-based warheads to achieve targeted covalency at p.E88 in the PDEδ model system. For compound design, we drew from insight gained during the development of potent reversible inhibitors, as well as two covalent inhibitor chemotypes previously reported (Figure S1).^20,33–35^ More reactive benzyl chloride-based counterparts were also considered for comparison during structure–activity analysis and refinement.

The lipoprotein binding chaperone PDEδ binds the prenylated termini of its lipidated cargos, including GTPases such as Ras, Rab, Rheb, and Rho proteins.^36^ PDEδ establishes the dynamic intracellular localization of these cargos, for instance, the Ras^33–35^ and Rheb proteins,^37^ and thereby plays an essential role in the regulation of their membrane localization and proper function. For inhibitor development targeting PDEδ, we^33–35^ and others (the Abankwa group,^38,39^ the Ismail group,^40^ and the Sheng group,^41–44^ ) have reported several potent (*K*_D_ values in the low nanomolar and picomolar range), reversible inhibitors of PDEδ,^33–35^ but their efficacy is intrinsically limited by the counteracting Arl2/3 GTPases, which upon allosteric binding stabilize an ‘open’ form of PDEδ such that even high-affinity ligands have an increased off-rate and are released from the lipoprotein binding site.^37^ PDEδ degraders that can act substoichiometrically circumvented the requirement for permanent binding PDEδ and showed improved antitumor activity as proof-of-concept for the design strategy.^45–48^ On the other hand, covalent-acting Deltasonamide-derived inhibitor bearing an isoxazolium warhead^19^ and Deltazinone-derived DeltaTag bearing an alkyl bromide warhead^20^ overcame ligand release by Arl2, yet their physiological and PK/PD stability largely limited further in vivo validation of antitumor activity.

Herein, we present the development and evaluation of benzyl fluoride-based ligands to achieve targeted modification at p.E88 in PDEδ. We demonstrate that ligand-focused structural fine-tuning may enable covalent modification of a low reactivity nucleophile with an inherently unreactive electrophile. The best fluoro-substituted ligand, termed Deltafluorine, achieves slow yet sustained covalent labeling of PDEδ (>85%) in vitro over a 7 day incubation at physiologically relevant conditions and induces a marked difference in phenotypic cellular profiles when compared to its nonfluorine-containing reversible counterpart. We also demonstrate that Deltafluorine impacts signal transduction through the MAPK family- and PI3K-Akt-mTOR signaling cascades, where PDEδ cargos such as the GTPases Ras and Rheb play a central role.^35,36,49–51^ Moreover, application of Deltafluorine in KRAS-dependent human and murine lung cancer models, both in cellulo and in vivo, proves antiproliferative activity of the inhibitor. These findings highlight the potential of fluoromethyl-substituted aryl systems for targeted covalent labeling of a specific carboxylate residue in proteins. With the observed promising biological effects of deltafluorine, it could also offer an exciting new therapeutic strategy for cancer treatment.

## Results

### Structural Development of Benzyl Halide Electrophiles for Covalent Targeting of a Glutamic Acid

To explore the possibility of covalently targeting PDEδ at its glutamic acid residue p.E88 with benzyl halide electrophiles, we examined the cocrystal structures of the reversible inhibitors Deltazinone derivative and Deltasonamide 1 and found that the distance of the common aryl moiety at the distal end of the structures to the p.E88 residue in the protein is approximately 3–6 Å, which suggested the suitability to attach a one-carbon unit reactive group (Figure 2a). Therefore, we structurally modified Deltazinone and Deltasonamide at the position proximal to p.E88 with benzyl halide electrophiles, yielding structures **1–22** (Figure 2b), and screened for covalent modification of PDEδ by matrix-assisted laser desorption/ionization (MALDI) intact protein mass spectrometry. In this screen, the degree of covalent modification was estimated from the relative intensity of the signals recorded for the formed adducts (Figure 2c, see Figure S2 for details). For structural development based on Deltazinone 1, initial investigations (**1–7**, Figure 2b) found relatively low labeling efficiency (Figure S2). While we observed a moderate extent of modification with the reactive benzyl chloride warheads (at most 70 ± 7% modification with **4b** by 24 h at 37 °C, Figure 2c), all benzyl fluoride-based warheads failed to label PDEδ under the same conditions. To improve the binding affinity of Deltazinone-based structures, we incorporated a piperidine ring into the side chain of the amide, which is found in both the more potent Deltarasin (*K*_D_ = 38 ± 16 nM) and Deltasonamide 1 (*K*_D_ = 203 ± 31 pM) and forms a hydrogen bond with the carbonyl group of Cys56 (C56) of PDEδ (Figure S1).^34,35^ This fragment-based hybrid design strategy^53^ was successfully employed in the development of Deltazinone-based DeltaTag in the covalent modification of PDEδ (Figure S1).^20^ However, although pyridylmethyl-chloride-containing amides **8–11** displayed covalent labeling efficiency up to 100% after 24 h, we could not observe any reactivity of the fluoride counterparts (Figures 2c and S2). For unsymmetrically substituted amides, we consistently observed rotamers due to slow rotation around the amide bond, which might limit the extent of covalent modification. Bioisosteric replacement of amides by sulfonamides^54^ overcame this limitation, as previously exemplified in the development of DeltaTag in covalent labeling of PDEδ.^20^ Consistent with the bioisosteric approach yielding sulfonamides **12**–**15**, we further improved labeling efficiency to 100% by 30 min with the pyridylmethyl chloride warhead in **13b** (Figures 2c and S2) and interestingly observed some very limited labeling of 2 ± 1% with the fluoride counterpart in **13a** at 24 h (Figure 2c).

**Figure 2:**
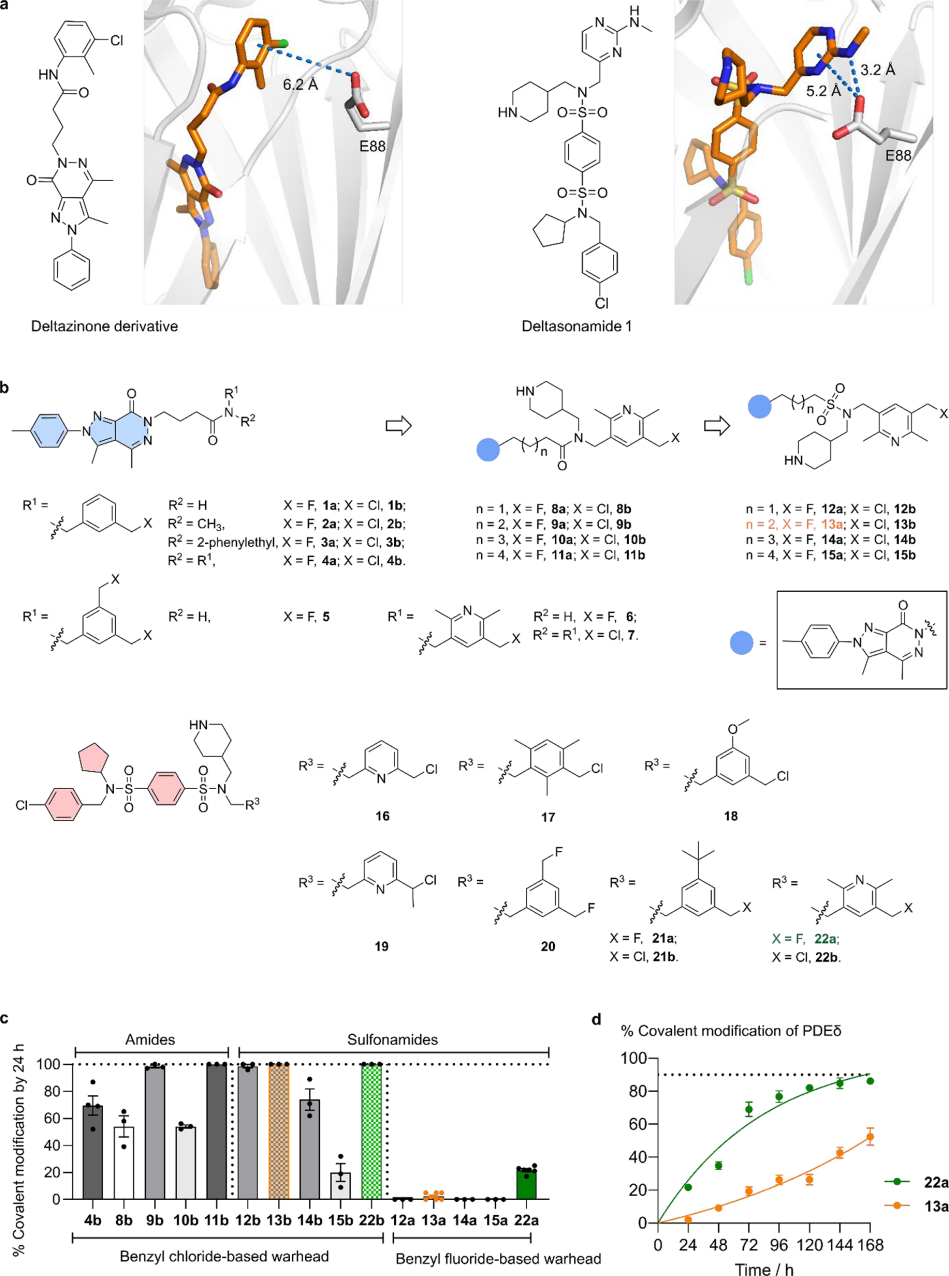
Structural development of halide electrophiles for covalent modification of PDEδ. (a) Cocrystal structures of Deltazinone derivative (PDB code: 5E80)^33^ and Deltasonamide 1 (PDB code: 5ML3)^34^ with PDEδ, highlighting the distance of the distal aryl ring to its glutamic acid residue p.E88. (b) Structures of Deltazinone-based and Deltasonamide-based compounds. IC_50_ values of PDEδ binding for **13a** and **22a** were determined to be 336 ± 106 nM and 27 ± 13 nM, respectively, by a competitive fluorescence polarization assay; their *K*_D_ values were further characterized with isothermal titration calorimetry (ITC) to be 667 ± 20 nM for **13a** and 148 ± 9 nM for **22a**, see Figure S3 for details. (c) Covalent modification [%] of representative compounds at 24 h as determined by MALDI mass spectrometry. PDEδ (20 μM) was incubated with compounds (60 μM, 0.6% DMSO) in HEPES buffer (20 mM HEPES, 150 mM NaCl, pH = 7.5) at 37 °C for 24 h before analysis by MALDI. Percentages of covalent adduct formation were estimated by the relative intensity of the respective peaks in MALDI spectra. Data are presented as mean ± standard error of the mean (*n* = 3, except *n* = 6 for **13a** and **22a**). (d) Kinetics of covalent modification of PDEδ (20 μM) by **13a** and **22a** (60 μM). Data are presented as mean ± s.e.m. (*n* = 6).

In parallel, structural modifications of the Deltasonamide structure similarly yielded a series of compounds (**16–18**, **21–22b**) that achieved fast labeling of PDEδ in vitro with reactive benzyl chloride-based warheads (100% by 24 h, Figures 2c and S2) and some modification with the fluoride counterpart (**22a**, 22 ± 1% by 24 h, Figures 2c and S2). Among compounds bearing benzyl fluoride-based warheads, the compound with the highest labeling efficiency was **22a**, which achieved a maximum modification of 86 ± 2% after 7 days (with an approximate second-order rate constant *k*_inact_/*K*_I_ = 5.4 M^–1^ s^–1^, Figure S2c), while the Deltazinone-based counterpart **13a** was limited to 52 ± 5% labeling over this extended time frame (Figure 2d).

Chloroalkane and chloromethyl-substituted aryl ring-based warheads are known to covalently label cysteine residues in proteins.^12,55,56^ We therefore investigated whether compounds **9b**, **11b**, and **13b**, which all showed rapid and full labeling of PDEδ by 24 h (Figures 2c and 3a), indeed modified p.E88 in its binding pocket instead of other reactive residues, including cysteines. We analyzed covalent adducts formed from PDEδ and these compounds with mass spectrometric evaluation of the peptides formed following a Glu-C digestion and identified the peptide sequence QKVQKVYFKGQCLEE with the exact mass, which corresponds to p.E88-modified adduct peptide (amino acids 78–89 of PDEδ, Figure 3b). The results indicated that **9b** and **13b**, both with four methylene units between the pyrazolopyridazinone fragment and the amide or sulfonamide, covalently modified PDEδ at a glutamate (either at p.E88 or at p.E89) with a higher probability for modification at p.E88 (Figure 3b). We also searched in parallel for other potential nucleophilic sites for covalent modifications (for example, other Glu, Asp, Cys, Thr, Ser, Tyr, Lys, and His residues) and did not observe other modified peptides (Table S1), suggesting that the selectivity of labeling the weakly nucleophilic glutamic acid p.E88 as compared to other more reactive and/or more accessible residues of PDEδ was conferred by the relatively high affinity of the core Deltazinone scaffold (Deltazinone 1: *K*_D_ = 8 ± 4 nM) to its binding pocket. It is noteworthy that for amide structure **11b** with six methylene units between the pyrazolopyridazinone fragment and the amide, we identified other possible modification sites, consistently at p.E114/S115 in addition to p.E88/E89 (Figure 3b and Table S1). While **9b** and **11b** demonstrated similar kinetics of modifying PDEδ over 24 h in vitro (Figure 3a), consistent with the results for the sulfonamides series **12**–**15**, the linker length of four methylene units was the most favorable. As with **9b**, the labeling kinetics represents selective modification at p.E88, while with **11b**, the comparable kinetics reflects heterogeneous modification at different nucleophilic residues, especially as p.E114 and p.S115 are located in proximity at the entrance of the PDEδ pocket and are surface exposed. This result reinstates the significance of structural optimization of the core scaffold for correct positioning and orientation of the electrophilic warhead toward the desired amino acid residue to control reaction specificity.

**Figure 3:**
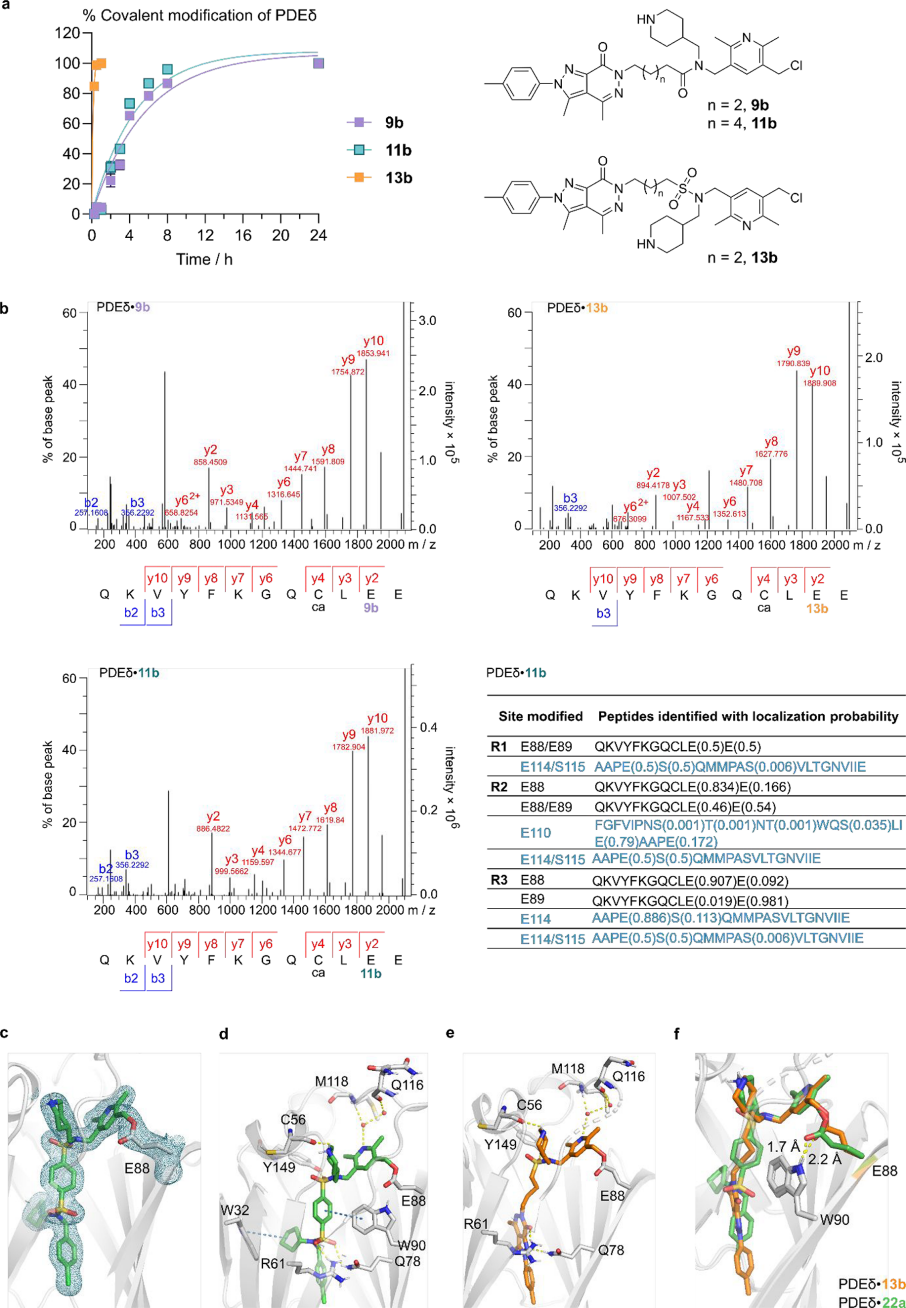
Structural basis for selectivity of covalent modification at p.E88 and reactivity of benzyl fluoride-based warheads. (a) Kinetics of covalent modification of PDEδ by **9b**, **11b**, and **13b**. PDEδ (20 μM) was incubated with compounds (60 μM, 0.6% DMSO) in HEPES buffer (20 mM HEPES, 150 mM NaCl, pH = 7.5) at 37 °C before analysis by MALDI. Percentages of covalent adduct formation were estimated by the relative intensity of the respective peaks in MALDI spectra. Data are presented as mean ± s.e.m. (*n* = 3). (b) Representative LC–MS/MS spectra of compound **9b**-, **11b**-, or **13b**-modified PDEδ peptides (amino acid 78–89), showing the modification site localization at p.E88. The sequence and detected fragments of the peptide are shown. Sequence-matched peaks are labeled in red and blue. Identified modifications at the amino acids are indicated below the peptide sequence—“ca” stands for carbamidomethylation of cysteine. Other **11b**-modified adduct peptides are shown in the table with the peptide sequence and localization probability. (c) 2*F*_0_–*F*_c_ map for ligand **22a** and p.E88 in cyan mesh (σ = 1.0). (d,e) Key bonding interactions of ligand **22a** (green) and **13b** (orange) to PDEδ, respectively. Hydrogen bonds are shown as yellow dotted line; aromatic-π interactions as blue dotted line. Bound water molecules are represented as red spheres. (f) Superimposition of X-ray cocrystal structures of covalent adducts, PDEδ·**22a** (1.9 Å, PDB code: 9RP7) and PDEδ·**13b** (1.8 Å, PDB code: 9RP6). See Table S5 for details of X-ray Crystallography Data Collection and Refinement Statistics.

Although **13b** exhibited favorable reactivity toward glutamate p.E88 in PDEδ, its instability in aqueous media and high reactivity with GSH (Figure S4) led us to discontinue further investigation. In contrast, in vitro stability and GSH reactivity tests demonstrated that the alkyl fluoride-based warhead in **22a** was highly stable and nonreactive in the presence of different nucleophiles such as glutathione, phosphate, acetate, and citrate in aqueous environments (Figure S4). The stability of the warhead and its demonstrated reactivity toward glutamate p.E88 in PDEδ prompted us to investigate **22a** further. We analyzed the cocrystal structure of the adduct formed from the treatment of **22a** and PDEδ. The crystal structure confirmed covalent binding in PDEδ’s binding pocket at p.E88, with unambiguous electron density showing an ester linkage between the ligand **22a** and p.E88 (Figure 3c). Inherited from the bis-sulfonamide structure of Deltasonamide 1, **22a** ligand was held in the binding pocket of PDEδ with hydrogen bonding with Cys56 (C56), Arg61 (R61), Gln78 (Q78), and Tyr149 (Y149) besides largely hydrophobic interactions (Figure 3d). The pyridyl nitrogen formed an additional hydrogen bond with a water molecule in a matrix held together by Met118 (M118) and Gln116 (Q116). While the Deltazinone counterpart **13b** with a reactive pyridyl-methyl chloride warhead comparatively achieved quantitative labeling by 30 min in vitro, we could not record a similar degree of modification by a fluoride replacement in **13a** even over an extended time frame of 7 days (Figure 2d). A comparison of the crystal structures of covalent PDEδ adducts formed with compounds **22a** and **13b** revealed that both ligands were anchored in the pocket by the same set of hydrogen bonds (as shown in Figure 3d and 3e). The aromatic-π interactions from Trp32 (W32) and Trp90 (W90) to **22a** (Figure 3d) provide enhanced stabilization, which might explain the observation of enhanced apparent binding affinity of **22a** (*K*_D_ = 148 ± 9 nM for **22a** and *K*_D_ = 667 ± 20 nM for **13a**, Figure S3). Overlay of the crystal structures indicated that the ligand from **13b** with the Deltazinone scaffold is located considerably deeper in the prenyl binding pocket of PDEδ as compared to **22a**. Additionally, the ester bond formed between ligand **13b** and the p.E88 residue is oriented at a different angle relative to the ester bond in covalent adduct PDEδ·**22a** (Figure 3f). The carbonyl group of p.E88 forms a hydrogen bond with the proximal Trp90 (W90), which was determined as 1.7 and 2.2 Å, respectively, in the covalent PDEδ·**22a** and PDEδ·**13a** adducts (Figure 3f). The significant difference in binding affinity, relative position of the ligands in the binding pocket of PDEδ, and the different orientation of the p.E88 side chain might provide an explanation why **22a** exhibited approximately twice the labeling rate of PDEδ compared to **13a** in vitro. The much higher binding affinity stabilized **22a** in the PDEδ binding pocket, and the higher positioning of **22a** (Figure 3f) and the closer distance of the aryl moiety to p.E88 (approximately 3 Å, Figure 2a) could orient the pyridylmethyl fluoride warhead in close proximity to the carboxylate residue with a high degree of overlap in their molecular orbitals, forcing a covalent reaction. In contrast, **13a** binds to PDEδ with a much weaker binding affinity, and the deeper and lower position of the Deltazinone scaffold in the binding pocket left the same warhead more distant from p.E88. The more reactive pyridylmethyl chloride in **13b**, with chloride being a much better leaving group, could forge a covalent modification with an altered orientation of p.E88 away from its interaction with W90 to accommodate a bond formation with adjusted angles, but we hypothesized that the probability of the same reaction could be much lower with the fluoride counterpart in **13a**. This observation highlights the potential for structural fine-tuning of small-molecule ligands to achieve targeted covalent labeling of a glutamic acid, even with an apparently inert benzyl fluoride-based warhead. We combined two parallel structural investigations based on Deltazinone and Deltasonamide derivatives and collectively chose to advance **22a** further to biological characterization. The compound was termed Deltafluorine to highlight its unique fluorine-containing structure.

### Deltafluorine Induces Unique Cellular Profiles in KRAS-Dependent Cancer Cell Lines

In the development of the reversible PDEδ inhibitor Deltasonamide 1, a correlation had been demonstrated between the inhibition of the cellular interaction of PDEδ and its client protein KRAS and the antiproliferative activity of Deltasonamide 1 in a panel of KRAS-dependent human cancer cell lines.^34^ The strongest antiproliferative activity of Deltasonamide 1 and PDEδ inhibitors Deltarasin and Deltazinone 1, was consistently observed in KRAS-dependent pancreatic ductal adenocarcinoma PA-TU-8902 cells.^33–35^ By analogy, we investigated the dynamic biological effects of Deltafluorine through the global proteome (Figure S5a–d) and phosphoproteome analysis in PA-TU-8902 cells (Figures 4c,d, and S5e,f). Upon treatment with Deltafluorine (**22a**) at 5 μM for 2 h in a live-cell setting, we identified 17 out of 3694 (0.46%) proteins differentially regulated (adjusted *p*-value ≤0.05, log_2_fold change ≥1 or ≤ −1, paired *t*-statistics-based FDR correction, Figure S5a). We observed the most significant and pronounced downregulation in PTB domain-containing engulfment adapter protein 1 (GULP1, log_2_fold change = −2.09, adjusted *p*-value = 1.62 × 10^–13^), which plays a role in endosomal trafficking and lipid transport, and syntaxin-10 (STX10, log_2_fold change = −1.82, adjusted *p*-value = 1.62 × 10^–13^), a Golgi apparatus associated membrane protein involved in intracellular protein transport. Additionally, we noted modulation in HLA-C (log_2_fold change = 3.97, adjusted *p*-value = 1.62 × 10^–13^), Golgin subfamily A member 1 (GOLGA1, log_2_fold change = 1.75, adjusted *p*-value = 0.03) and WASH complex subunit 2C (FAM21C, log_2_fold change = −2.03, adjusted *p*-value = 0.04), all of which are involved in the endosomal pathway and membrane trafficking of protein cargos. When analyzing these hits for clustering or functional relatedness, we performed a Reactome pathway overrepresentation analysis of the significant hits,^57^ which revealed notable regulation of pathways related to vesicle-mediated transport across the extended Golgi-Endoplasmic Reticulum (ER) membrane system (see Figure S5b–d for overview and Table S2 for details), besides general interferon-mediated signal translation pathways (see Figure S5e,f and Table S2 for details). The PDEδ lipoprotein chaperone binds to the prenylated termini of several GTPases, including Ras^33–35^ and Rheb^37^ family members, influencing their intracellular trafficking between membrane compartments. This regulation of membrane localization and signal transduction may provide a link to the observed biological activity of Deltafluorine.

**Figure 4:**
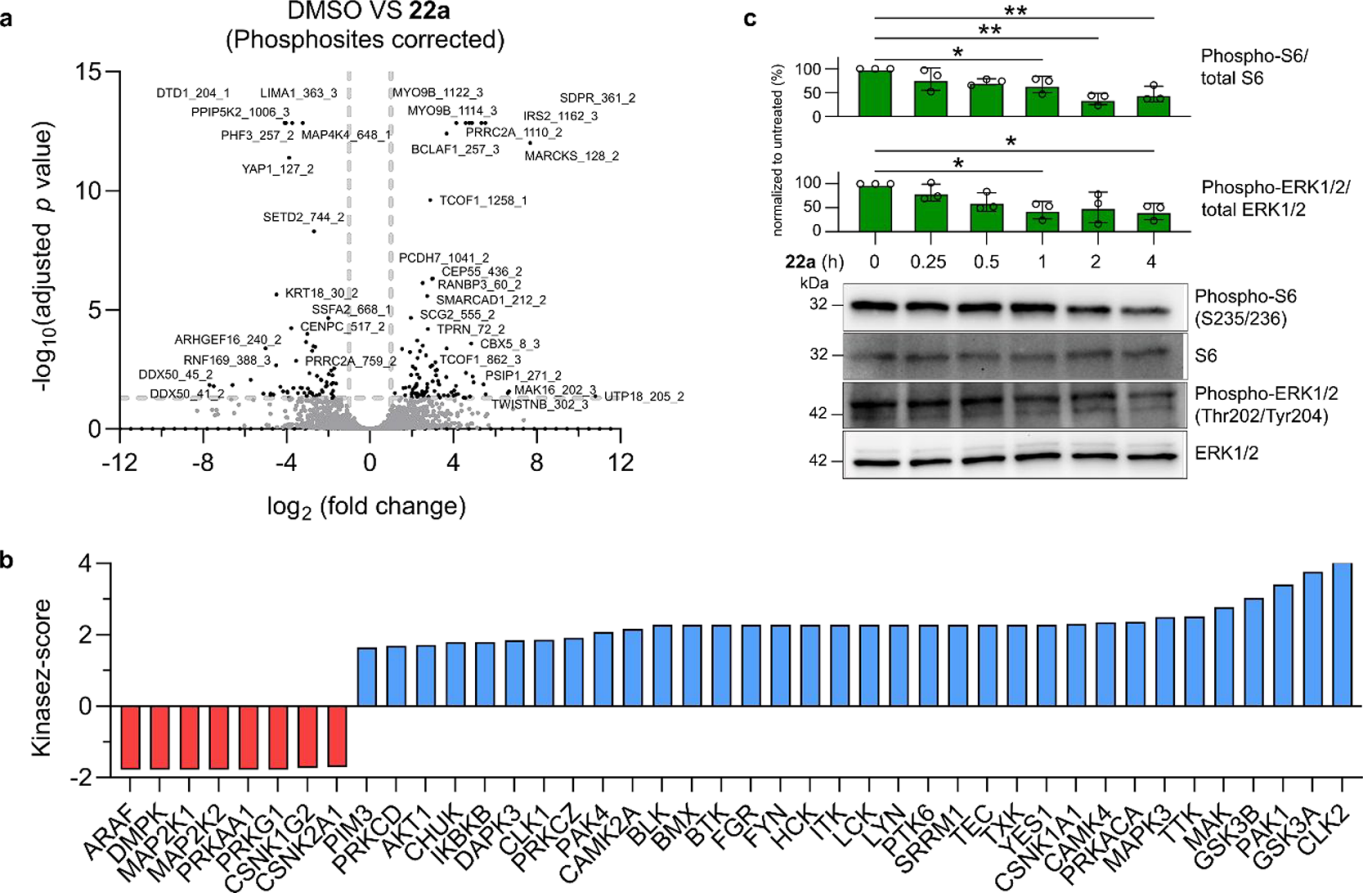
Deltafluorine (**22a**) impairs MAPK and Akt-mTOR pathways. (a) Phosphoproteome profiling. Volcano plot with -log_10_ (adjusted *p*-value) plotted against log_2_fold change between DMSO-treated and compound-treated conditions is shown. Significant hits (adjusted *p*-value ≤0.05, log_2_fold change ≥1 or ≤ −1) are labeled with their gene names and phosphosites localization. (b) Plot of kinase z-scores (*p* ≤ 0.05) after kinase-substrate enrichment analysis of significant phosphosites by KSEA App with NetworKIN (substrate count cutoff = 2, NetworKIN score cutoff = 1).^59–61^ (c) Immunoblot analysis examining phosphorylation of ERK1/2 at Thr202/Tyr204 and phosphorylation of S6 at S235/236 in PA-TU-8902 cells upon a time-course treatment with 5 μM Deltafluorine. Data presented as mean ± standard deviation (*N* = 3, see Figure S6 for details). Ordinary one-way ANOVA, multiple comparisons, *p-value < 0.05, and **p-value < 0.01.

To gain further insight into the molecular basis downstream of PDEδ-Ras and PDEδ-Rheb inhibition, we employed Phospho-Analyst to analyze the phosphoproteome monitored in parallel with the global proteome upon Deltafluorine treatment.^58^ With background correction to the global proteome, we identified 161 out of 8062 (2%) phosphosites significantly changed with 5 μM Deltafluorine (**22a**) treatment for 2 h (adjusted *p*-value ≤0.05, log_2_fold change ≥1 or ≤ −1, paired *t*-statistics-based FDR correction, Figure 4a). From kinase-substrate enrichment analysis (KSEA) of the significant changes in phosphorylation,^59–61^ we observed significant regulation of kinases related to the MAPK family signaling cascades (PhosphoSitePlus data set with NetworKIN cutoff = 1, substrate count cutoff = 2, *p* ≤ 0.05, Figure 4b). In particular, we noted strong downregulation of Ras-mediated A-Raf kinase (encoded by ARAF) and dual specificity mitogen-activated protein kinases 1 and 2 (MAP2K1 and 2), as well as the Ras homologue gene family member A (RhoA)-implicated cGMP-dependent protein kinase 1 (PRKG1), all of which presented with a kinase z-score of −1.78 (Figure 4b). Although we observed a higher number of upregulated compared to downregulated kinases, pathway analysis of the upregulated kinases suggested that their increased activity was likely due to secondary effects of intracellular signaling (Table S3). Further input of the significantly suppressed kinases into Reactome for pathway overrepresentation analysis^57^ additionally confirmed the marked downregulation of oncogenic MAPK signaling (number of reactions found = 33, *p*-value = 2.86 × 10^–5^, see Figure S5e,f and Table S4 for details) upon Deltafluorine (**22a**) treatment. Voronoi visualization of the Reactome pathway analysis also revealed that, in addition to Ras-mediated MAPK signaling, there was considerable suppression in the Rheb-mediated mTOR pathway (number of reactions found = 5, *p*-value = 0.03, see Figure S5e,f and Table S4 for details), which is also connected to Ras signaling.

Consistently for Deltafluorine, the downregulation of the Ras pathway downstream of MAPK and the related PI3K-Akt-mTOR signaling pathway was evident from phosphoproteome analysis. Additionally, we performed immunoblot analysis examining phosphorylation of downstream targets of the MAPK and PI3K pathways following a time-course of exposure of PA-TU-8902 cells to Deltafluorine (Figure 4c). Our results demonstrate that Deltafluorine treatment leads to decreased phosphorylation of ERK1/2 and S6 dephosphorylation between 1 and 4 h. These timed dependencies are in line with our proteomic analysis (Figure 4a,b), and thus provide additional support on the activity of Deltafluorine on suppression of Ras-related signaling.

Therefore, we further examined the antiproliferative activity of Deltafluorine along with Deltasonamide 1 in a panel of human cancer cell lines, selected for their varied KRAS dependency,^62–64^ mutational status, and diverse tissue origin (Figure 5). We first demonstrated target engagement of Deltafluorine with PDEδ by means of a cellular thermal shift assay (CETSA) showing significant thermal stabilization of PDEδ upon compound treatment (in-cell CETSA with **22a** treatment at 10 μM for 2 h, melting temperature shift Δ*T*_m_ = 16.6 ± 3.2 °C, paired *t*-test *p = 0.01, Figure 5a). Morphological cellular profiling using the Cell Painting assay (CPA)^65^ revealed that for both Deltafluorine and Deltasonamide 1, cytotoxicity was observed for concentrations above 5 μM upon 20 h treatment, and that at nontoxic doses, both compounds showed high similarity to the previously identified lysosomotropic profile (Figure S7a).^66,67^ The lysosomotropic activity and cytotoxicity of Deltafluorine and Deltasonamide 1 could mask the specific cellular effects resulting from ligand–target interactions, for in single-dose treatment, we did not observe significant changes in their cellular potency (72 h IC_50_ of Deltafluorine = 3.1 ± 0.1 μM versus IC_50_ of Deltasonamide 1 = 3.8 ± 0.1 μM in PA-TU-8902 cells, Figure S7b). Therefore, we investigated their antiproliferative effects by a wash-out experiment with a sequential treatment regimen mimicking therapeutic dosing and noted that the superiority in cellular activity of Deltafluorine over Deltasonamide 1 was only evident under the wash-out conditions (Figures 5b and S7c). By measuring cell confluency over time with real-time live-cell imaging by Incucyte, we observed a marked difference in cellular potency of Deltafluorine and Deltasonamide 1 with a general trend of correlation with KRAS dependency (Figure 5c, see Figure S8 for details). In-cell target engagement for the short-term treatment regime employed in the wash-out experiments was supported by the observation that growth impairment of HAP1 PDEδ knockout cells by Deltafluorine (**22a**) was less pronounced compared to HAP1 wild type cells at concentrations 5–10 μM for 4 h treatment (paired *t*-test, two-tailed ****p-value < 0.0001, Figure S7d). Strong inhibitory activity of Deltafluorine (**22a**) was observed in KRAS-dependent pancreatic ductal adenocarcinoma cell lines PA-TU-8902 and MIA PaCa-2 cells with an average growth inhibition of 84 ± 2% and 87 ± 4%, respectively, upon repeated dosing and washing at 5 μM concentration (4 h treatment +20 h wash-out regimen) over 4 days. The reversible nonfluoride-containing PDEδ inhibitor Deltasonamide 1 failed to retain the antiproliferative activity under the same washing conditions (unpaired *t*-test ****p < 0.0001, Figure 5b,c). The differential sensitivity to the treatment regimen was also observed in KRAS-dependent SW480 (colorectal) and NCI-H358 (lung) cells, while the difference diminished in KRAS-independent PANC-1 (pancreatic ductal), LS-174T (colorectal), A549 (lung), and KRAS wild-type-expressing BxPC-3 (pancreatic) and HT-29 (colorectal) cells (Figure 5c). Deltafluorine was slightly more active in the aforementioned KRAS mutant and dependent cell lines in comparison to the HRAS mutant Hs 578T cell line, with an approximately 2-fold difference in their IC_50_ values, while no difference in IC_50_ was observed to the BRAF mutant HT-29 cells (Figure S8). Exceptions to the correlation between differential sensitivity and KRAS dependency were noted in HCT116 (colorectal), NCI-H441 (lung), and SK-LU-1 (lung) cells (Figure 5c). HCT116 and SK-LU-1 cells were classified as KRAS independent, but their degree of Ras dependency was at the borderline of cutoff^62–64^ (Figure 5c, 90 ± 3% growth inhibition by **22a** vs 10 ± 7% by Deltasonamide 1 at 5 μM in HCT-116, unpaired *t*-test ****p < 0.0001; 76 ± 5% growth inhibition by **22a** vs 28 ± 21% by Deltasonamide 1 at 5 μM in SK-LU-1, unpaired *t*-test *p = 0.02). For KRAS-dependent NCI-H441 cells, there was no substantial difference in the cellular response observed between the pair (Figure 5c, 16 ± 4% growth inhibition by **22a** vs 7 ± 2% by Deltasonamide 1 at 5 μM, unpaired *t*-test *p* = 0.03). This discrepancy may be related to the abundance and balance level of Ras isoforms, for NCI-H441 cells exhibit elevated levels of KRAS4a, while PDEδ is only capable of translocating the other splice variant KRAS4b between membrane compartments.^68,69^

**Figure 5:**
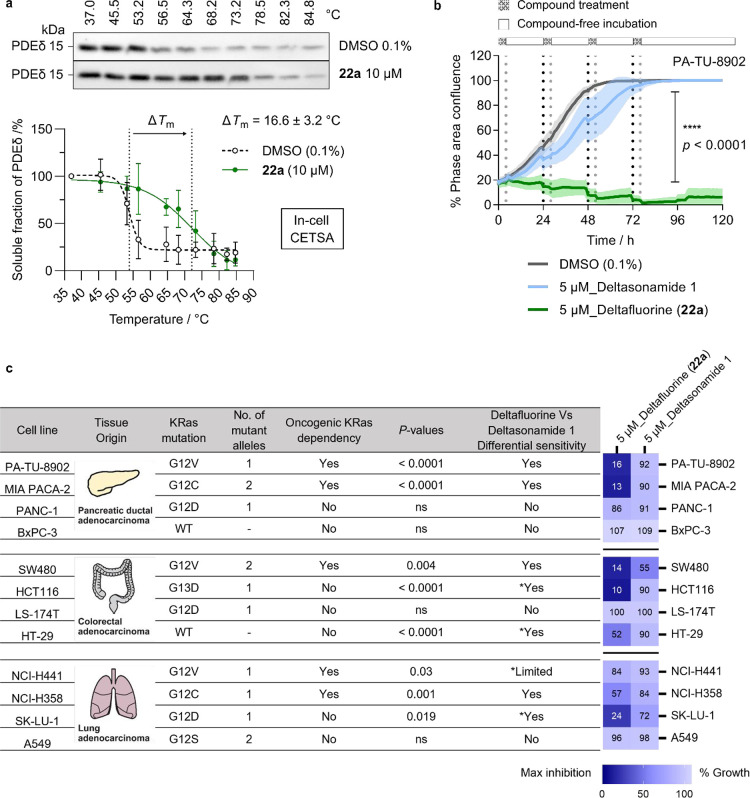
Profiling of Deltafluorine (**22a**) in KRAS mutant cell lines. (a) In-cell CETSA. Jurkat cells were treated with the compound (**22a**) at 10 μM or vehicle (0.1% DMSO) for 2 h under live-cell settings before cell lysis and heat treatment. Intensities of the PDEδ immunoblot bands were analyzed by Image Lab and normalized to the first band of each condition. Data are presented as mean ± standard deviation, representative of three biological replicates (*n* = 3, see Figure S11 for details). (b) Scheme and representative cellular growth curves of the wash-out assay in PA-TU-8902 cells. Time-points for compound addition are marked with black dotted lines and compound removal by washing with fresh culture medium are marked with gray dotted lines. Data are presented as mean ± standard deviation, representative of three biological replicates (*n* = 3). (c) Overview of growth inhibition in the wash-out assay for the selected cell panel. Cell growth was determined by area under the curve integration at 120 h after the first dose of compound treatment monitored by real-time cell analysis of percent phase area confluence, normalized to DMSO control. Mean values of relative growth were plotted for the heat map, representative of three biological replicates (*n* = 3). Unpaired *t*-tests were carried out between Deltafluorine (**22a**) and Deltasonamide 1 treatment conditions with two tailed *p*-values denoting differential sensitivity of Deltafluorine (**22a**) in each cell line. Exceptions to the general correlation between differential sensitivity of Deltafluorine (**22a**) in comparison to Deltasonamide 1 and KRAS dependency are marked with * in the table.

### Deltafluorine Demonstrates In Vivo Single-Agent Therapeutic Efficacy in a Kras-Driven Mouse Model of Lung Adenocarcinoma

Given the demonstrated general correlation between in cellulo investigation of Deltafluorine and cell line KRAS dependency, as well as the modulation of Ras downstream signaling of the MAPK and of the PI3K-mTOR pathway, we further investigated Deltafluorine in vivo. First, the Deltafluorine dosage was evaluated in healthy wild-type mice to assess pharmacokinetics and tolerability. Intraperitoneal administration showed the highest bioavailability (Figure S9a–c). A 21 day repeat dose study in wild-type animals 15 mg/kg q.d. i.p. was generally well tolerated with only transient signs of discomfort and a maximum mean body weight loss of 10% (Figure S9d). With Deltafluorine being a tool compound and not yet a fully pharmacologically optimized drug candidate, we selected the highest tolerated dose for further experiments. For a proof-of-concept study, we utilized an autochthonous, conditional Kras^LSL.G12D/wt^;Trp53^fl/fl^ (KP) mouse model.^70,71^ In this mouse model for highly aggressive lung adenocarcinoma, we induced tumor formation by intratracheal Adeno-CMV-Cre instillation, leading to Kras^G12D^ activation and Trp53 deletion (Figure 6a), and monitored tumor onset via μCT imaging. Once mice developed measurable tumor lesions (pretreatment CT), we randomized mice 1:1 to either Deltafluorine, 15 mg/kg i.p., q.d. for 21 days, or left them untreated. Of note, all mice included in this study had comparable cancer onset and tumor burden before treatment start, as assessed by μCT imaging (Figure S10). All KP lung cancer mice received a post-treatment μCT scan within 6 weeks of treatment initiation. For accurate tumor volume measurements, we reconstructed the μCT scans from the post-treatment time point and measured the volume of detectable lung tumors with 3D image modeling. This sensitive computer-aided method of tumor volumetry calculation revealed that the average tumor load after 3 week Deltafluorine treatment was significantly reduced by 42% compared to the untreated KP control group (Figure 6b,d). We further normalized tumor load with the corresponding lung volume of each animal to account for variability in the lung sizes of tested mice. Strikingly, while tumors occupied 47.5% of the lung volume in untreated KP mice, Deltafluorine-treated KP-tumor-bearing animals exhibited only 21.6% tumor burden (Figure 6c,d). Although future investigations are needed to confirm that the activity of Deltafluorine in cellulo translates to its in vivo effect, the current data demonstrated the clear effectiveness of short-term Deltafluorine treatment in reducing tumor growth in this highly aggressive lung adenocarcinoma in vivo model.

**Figure 6:**
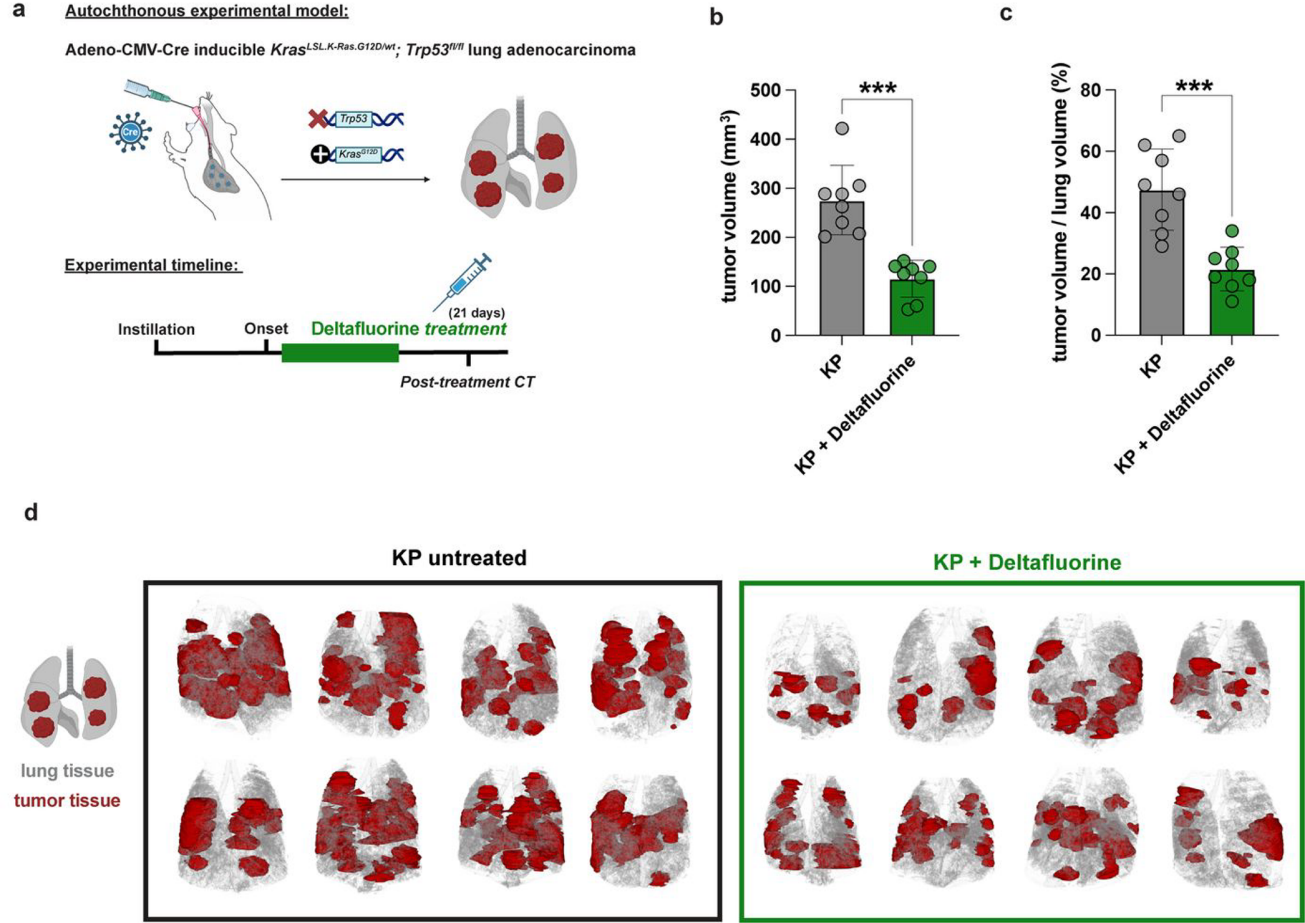
In vivo study of Deltafluorine (**22a**) in Kras^LSL.G12D/wt^;Trp53^fl/fl^ (KP) mouse model. (a) Experimental setup. (b) Tumor volumes were measured in post-treatment μCT scans of lungs from eight mice per group. Unpaired *t*-test ***p < 0.001. (c) Tumor volumes from (b) were normalized to total lung volume of the respective lung, resulting in percentage of tumor-occupied lung volume. Unpaired *t*-test ***p < 0.001. (d) 3D renderings of analyzed lung μCT scans. Gray represents lung tissue, white air, and red tumor tissue.

## Discussion

KRAS-mutated cancers were considered undruggable for a long time. The FDA-approved non-small cell lung cancer drugs, sotorasib^72^ and adagrasib,^73^ along with the more recently discovered MK-1084,^74^ covalently target the acquired cysteine in the K-Ras^G12C^ mutation, thereby locking K-Ras in a signaling-incompetent state and effectively inhibiting its activity. Furthermore, the recently reported first-in-class small molecule inhibitor BBO-8520 engages KRAS^G12C^ in both active and inactive conformations.^75^ In contrast, covalent inhibition of K-Ras^G12D^, the most prevalent single variant in K-Ras-driven tumors, has remained elusive due to the more challenging carboxylate-targeting chemistry. Isolated cases of covalent inhibitors for K-Ras^G12D^ have been reported—Yu et al. have reported a dual K-Ras^G12D/G12C^ inhibitor bearing an epoxide warhead;^5^ Shokat et al. have employed a malolactone-based warhead for mutant-selective targeting of K-Ras^G12D^;^6^ and RMC-9805, which bears a trisubstituted aziridine warhead, has also been investigated in preclinical models of K-Ras^G12D^ cancers.^9^ We were hence interested in the expansion of carboxylate-targeting chemistry for covalent inhibitor design and exemplified the utility of a fluoromethyl-substituted aryl warhead with a PDEδ inhibitor Deltafluorine targeting its binding site, glutamate p.E88. For PDEδ as a lipoprotein chaperone involved in membrane localization and hence proper signaling of GTPases, including Ras,^33–35^ targeting PDEδ may also offer an alternative strategy to treat K-Ras-driven cancers, evidenced from the clear effectiveness of Deltafluorine demonstrated in the in vivo Kras^LSL.G12D/wt^;Trp53^fl/fl^ (KP) mouse model of lung adenocarcinoma.

From structure-based analysis of reversible PDEδ inhibitors Deltazinone and Deltasonamide, we explored the possibility of benzyl fluorides as covalent warheads to achieve targeted inhibition of a glutamate residue p.E88 in PDEδ’s binding pocket. Being otherwise stable and unreactive toward other nucleophiles, the specific reactivity of Deltafluorine to PDEδ’s p.E88, might be attributed to its high binding affinity and optimal positioning within the binding pocket, with favorable ligand disposition ensuring close proximity between the warhead and p.E88 to facilitate the reaction. In comparison, the Deltazinone counterpart, with approximately 10-fold lower binding affinity and more distant targeting of p.E88 in PDEδ (∼3 Å further), could only achieve ∼50% covalent inhibition under the same conditions. The Deltasonamide-derived Deltafluorine, with a pyridylmethyl fluoride-based warhead, covalently bound to PDEδ at p.E88 with >85% modification efficiency in a sustained rate over a 7 day course in vitro. We approximated its apparent second order rate constant, *k*_inact_/*K*_I_ to be 5.4 M^–1^ s^–1^, which is admittedly extremely slow as compared to many TCIs with reported *k*_inact_/*K*_I_ in the range of 10^5^–10^7^ M^–1^ s^–1^.^76^ For PDEδ’s catalytic role in translocating membrane-associated GTPases,^37^ a high level of target inhibition would be required for any observed cellular activity.^33^ There seemed to be an apparent discordance between the very slow covalent reactivity observed in vitro and the distinct cellular phenotype under the wash-out assays with 4 h incubation for each dose. This could in part be explained by cellular PDEδ concentration in the nanomolar range^38^ coupled with treatment in the micromolar range, leading to a pseudo-first reaction kinetic. We also reasoned that PDEδ’s slow cellular turnover (synthesis half-life = 113 h; degradation half-life = 40 h)^77^ compensated for the weak covalent reactivity of Deltafluorine and that its covalency-driven effect could be accumulated to a substantial amount for biological effects with repeated dosing and sustained exposure. Therefore, using a wash-out assay mimicking a therapeutic dosing regimen, we observed a distinct phenotypic difference between Deltafluorine and the reversible nonfluoride-containing counterpart Deltasonamide 1 in suppressing the proliferation of KRAS-dependent cell lines. Statistical analysis of the dynamic changes in the proteome revealed significant regulation in the endosomal-related membrane trafficking of protein cargos, with notable suppression of KRAS-related MAPK- and PI3K-Akt-mTOR signaling pathways. These in cellulo observations upon Deltafluorine interference were consistent with PDEδ inhibition^20,33–35^ and aligned with PDEδ’s role as a lipoprotein chaperone regulating membrane localization of GTPases, including Ras.^33–35^ The link between a unique phenotypic change with Deltafluorine treatment in cells and its covalent mode of action, however, remains unclear, which is most likely due to a mixed effect of noncovalent and covalent inhibitions to PDEδ.

To evaluate Deltafluorine in a clinically relevant in vivo context, we employed the autochthonous Kras^LSL.G12D/wt^;Trp53^fl/fl^ (KP) mouse model of lung adenocarcinoma. Lung cancer remains the leading cause of cancer-related mortality,^78^ and KRAS mutations are found in approximately 32% of cases,^79^ rendering this mouse strain a highly relevant model system to test the effectiveness of Deltafluorine in cancer. Importantly, this mouse model represents extraordinarily aggressive lung adenocarcinoma with a mean survival of only 120 days, and the strain has remained incurable since its implementation over 15 years ago. Prior publications in the autochthonous KP model tested the effect of chemotherapy. Cisplatin was shown to yield only marginal tumor control,^80^ while paclitaxel–carboplatin was ineffective relative to untreated controls.^81^ This well-documented chemoresistance reflects the distinctive KP tumor biology, establishing a high yet appropriate bar for proof-of-concept evaluations aligned with clinical need and translational development in this very aggressive lung tumor model, in which both KRAS and TRP53 are altered. From a clinical point of view, this is exactly the patient population with the biggest need for novel therapeutic approaches. Consistently, published trajectories for untreated cohorts and for standard chemotherapies in KP mirror the behavior observed in our study and experience with the model.^81,82^

Remarkably, we observed a marked reduction in tumor growth following a short-term Deltafluorine treatment. This robust in vivo effect, together with minimal observed side-effects, indicates the compound’s therapeutic potential. We therefore presented a generally interesting covalent mode of action with a fluoromethyl-substituted aryl ring system reacting with a glutamate in a target protein binding site. Notably, with elaborate downstream biological characterizations both in cellulo and in vivo, the best compound, Deltafluorine, bearing a fluoromethyl-pyridyl ring, also demonstrates promising therapeutic potentials, for example, for targeted cancer treatment approaches.

## Materials and Methods

### Materials and Equipment

Materials, reagents, and equipment are listed in Supporting Information, Section 4—Material List.

### Compound Synthesis and Analysis

Details related to compound synthesis and analysis can be found in Supporting Information, Section 3—Chemical Synthesis. All compounds investigated were isolated and purified and were ≥95% pure by HPLC analysis.

### Protein Purification

All proteins were expressed in *Escherichia coli* strain Rosetta (BL21DE3). Competent cells were transformed with the respective pET plasmids and inoculated on a cell plate with TB medium supplemented with 100 μg/mL ampicillin and 30 μg/mL chloramphenicol for overnight incubation at 37 °C. A single colony was used to inoculate TB medium supplemented with 100 μg/mL ampicillin and incubated at 37 °C with simultaneous shaking overnight. The next day, 5 L of TB medium was inoculated with 50 mL of the Rosetta suspension and incubated at 37 °C until an OD ∼1.0. Cells were induced at OD ∼1.0 with 100 μM isopropyl β-D-1-thiogalactopyranoside (IPTG) and incubated at 20 °C for 7 h. Cells were harvested and lysed in lysis buffer (30 mM Tris–HCl, pH = 7.5, 150 mM NaCl and 1 mM β-mercaptoethanol, 1 mM PMSF, i.e., phenylmethylsulfonyl fluoride) with sonication on ice. Supernatant of histidine-tagged protein was collected by centrifugation at 13,000*g* and 10 °C for 35 min and subsequently loaded onto a Ni-NTA column (QIAGEN) and eluted with elution buffer (30 mM Tris–HCl, pH = 7.5, 150 mM NaCl, 1 mM dithiothreitol (DTE), and 250 mM imidazole), followed by gel filtration on a Superdex G75 S26/60 column using elution buffer without imidazole. Protein purity was checked by SDS-PAGE.

### MALDI Mass Spectrometry

PDEδ (20 μM) was incubated with compounds (60 μM) in HEPES buffer (20 mM HEPES, 150 mM NaCl, pH = 7.5) with 0.6% DMSO at 37 °C for the specified time (2, 4, 6, 8, 24 h, and subsequently every 24 h up to 7 days). The solution was briefly centrifuged before a sample was taken at the designated time for analysis by MALDI. A saturated solution of sinapinic acid (SA) in EtOH was used as matrix A, and it was added on an MTP 384 ground steel target plate (Bruker) and dried in air. A saturated solution of SA in 30/70 acetonitrile (ACN)/H_2_O with 0.1% TFA was used as matrix B. One microliter portion of the sample was mixed with 2 μL of matrix B, and 1 μL of this mixture was placed on top of matrix A on the MTP 384 ground steel target plate and dried in air. Mass spectra were obtained over the *m*/*z* range 15,000–25,000 using a Bruker UltrafleXtreme XIAL DI-TOF/TOF mass spectrometer. Percentages of covalent adduct formation were estimated by the relative intensities of the respective peaks in MALDI spectra.

### Fluorescence Polarization Assay

Binding to PDEδ was validated and quantified by means of a direct displacement assay employing a fluorescence labeled analogue of the HMG-CoA reductase inhibitor atorvastatin (FA probe), which has previously been shown to also bind to PDEδ.^83^ Reported *K*_D_ of FA probe = 7.1 ± 4 nM and experimentally validated *K*_D_ of FA probe = 10.6 ± 2.3 nM. The *K*_D_ and IC_50_ values against PDEδ were determined by competitive fluorescence polarization assay, adapted from the method we described before.^35^ IC_50_ values were generated and fitted with GraphPad Prism 9.2 (GraphPad software, USA) using a four-parameter variable slope nonlinear regression curve fit. To serially diluted solutions of compound in PBS buffer (containing 0.05% Chaps, 1% DMSO) in a black, nonbinding round-bottom 384-well plate (Corning #4514) (10 μL/well) was added an equal volume of premixed solution of PDEδ (80 nM) and FA probe (48 nM) in PBS buffer (containing 0.05% Chaps, 1% DMSO) so that the final concentration of compound was adjusted to the range of 0–500 nM, PDEδ to 40 nM, FA probe to 24 nM and DMSO to 1% for all conditions. The sealed plates were centrifuged, shaken (600 rpm) overnight at room temperature, and briefly centrifuged again before measurement of fluorescence polarization values (excitation wavelength at 485 nm and emission wavelength at 535 nm) by a plate reader at 25 °C (Tecan SPARK).

### Isothermal Titration Calorimetry

ITC was performed using the MicroCal PEAQ-ITC system (Malvern) at 25 °C. Purified PDEδ was buffer-exchanged into PBS buffer containing 1 mM TCEP using a 3K Amicon Ultra centrifugal filter (Millipore). The buffer exchange was repeated ten times to ensure complete removal of interfering components. The final protein sample was diluted in the same phosphate buffer prior to titration. 300 μM protein in the buffer was loaded into the syringe, while 30 μM **22a** or 40 μM **13a** in the buffer was loaded to the cell. All samples were adjusted to 25 °C and degassed before loading. Titrations were performed at 25 °C with one injection of 0.4 μL, followed by 18 injections of 2 μL. Experiments were performed with three biological replicates (*n* = 3). Data were analyzed using the MicroCal PEAQ-ITC Analysis software and were plotted using GraphPad Prism 9.0 (GraphPad, USA).

### Compound Stability in Aqueous Buffers and in the Presence of GSH

Compound (1 mM) was incubated with the respective buffers (HEPES 20 mM, 150 mM NaCl, pH = 7.5; 0.2 M sodium acetate buffer, pH = 5.6; 0.2 M sodium citrate buffer, pH = 6.2; 0.2 M potassium phosphate buffer, pH = 7.4, and 1 mM EDTA, with or without 10 mM GSH), with 1% DMSO adjusted for all conditions. The resulting solutions were incubated with shaking (600 rpm) at 37 °C and protected from light. At the respective time points (0, 2, 4, 6, 8, 24, 30, 48, 56, and up to 72 h), a sample was taken and diluted in acetonitrile and analyzed by HPLC-MS (Agilent Technologies 1290 Infinity, 6150 Quadrupole LC/MS). Percent (%) compound remaining in the solution was estimated by the ratio of areas under the curve.

### Mass Spectrometry Analysis of Covalent Peptide Adducts of PDEδ after Glu-C Digestion

Covalent adducts of PDEδ or unbound vehicle DMSO-treated PDEδ (1.5 μg/sample) were denatured, reduced, alkylated, and digested by Glu-C and desalted before analysis by mass spectrometry. For denaturation, 4 μL of each sample was added to 18 μL of 8 M guanidine hydrochloride (cas 50-01-1, Carl Roth, #0037.1) solution (to a final 6.5 M) and boiled for 15 min at 95 °C. For reduction, the solution was then cooled and added to 0.5 μL of 50 mM dithiothreitol (DTT, cas 3483-12-3, Gerbu Biotechnik, #1008-100g) solution (to a final 1 mM DTT) and incubated for 20 min at 60 °C. Subsequently, for alkylation, 2.5 μL of 50 mM 2-chloroacetamide (CAA, cas 79-07-2, Sigma-Aldrich, #22790) was added to each sample (to a final 5 mM CAA) and incubated for 30 min at room temperature with protection from light. For each sample, 225 μL of 20 mM ammonium bicarbonate (cas 1066-33-7, Sigma-Aldrich, #A6141–500g) solution was then added to dilute the concentration of guanidine hydrochloride to less than 0.8 M. For digestion, 1.5 μL of Glu-C (0.05 μg/μL, Promega V1651) was added to each sample (1:20 enzyme to protein, w/w) and incubated overnight at 37 °C, 400 rpm with protection from light. On the next day, the reaction was quenched by adding 5 μL of 10% trifluoracetic acid (TFA, cas 76-05-1, Sigma-Aldrich, #302031), and samples were desalted by stage tip purification with C18 extraction disks (Empore high performance extraction disks, 47 mm, 3 M Bioanalytical Technologies #2215). Each stage tip (2 layers of C18 disks) was activated by 100 μL of methanol, washed once by 100 μL of buffer B (containing 0.1% formic acid, 80% acetonitrile in water), followed by twice washing with 100 μL of buffer A (containing 0.1% formic acid in water) prior to sample loading. Loaded stage tip was further washed once with 100 μL of buffer A, and sample was eluted with 20 μL of buffer B, with centrifugation at 4000 rpm at room temperature for 5 min and then dried by SpeedVac at 30 °C. All details related to nanoHPLC-MS/MS analysis and data evaluation can be found in Supporting Information—Methods section.

### X-ray Crystallography

Compound **13b** was cocrystallized with PDEδ by incubating 2 mM of small molecule (10:1 compound to protein) with 200 μM of PDEδ in HEPES buffer (HEPES 20 mM, 150 mM NaCl, pH = 7.5), with final 1% DMSO, at 37 °C, 600 rpm for 30 min (when complete covalent modification was verified by MALDI-TOF mass spectrometry measurement), followed by washing and concentration with Amicon 3k filters (Millipore, UFC5003BK) in protein buffer (containing 30 mM Tris–HCl, 150 mM NaCl, 1 mM β-mercaptoethanol, pH = 7.5) to approximately 20 g/L.

Compound **22a** was cocrystallized with PDEδ by incubating 2 mM of small molecule (4:1 compound to protein) with 500 μM of PDEδ in TRIS buffer (25 mM TRIS–HCl, pH 7.5, 150 mM NaCl, and 3 mM DTE), with final 2.5% DMSO, at 37 °C, 400 rpm, until complete covalent modification was verified by MALDI-TOF mass spectrometry measurement. Precipitates were removed via centrifugation at 20,000 g and 4 °C for 5 min, followed by washing and concentration with Amicon 3k filters (Millipore, UFC5003BK) in protein buffer (containing 30 mM Tris–HCl, 150 mM NaCl, 1 mM β-mercaptoethanol, pH = 7.5) to approximately 20 g/L.

0.1 μL portion of each protein solution was mixed with 0.1 μL of precipitant solution in a sitting-drop setup (MRC 3-drop plates, Jena Bioscience, UK for PDEδ·**13b** and iQ plates, SPT Labtech for PDEδ·**22a**), and crystals were obtained from a self-made crystal optimization plate (0.1 M NaOAc, pH = 4.71, 5.6% w/v PEG4000, 30% v/v glycerol) for PDEδ·**13b** and from a Qiagen Classics suite (20% (w/v) PEG3350 and 0.2 M potassium formate) for PDEδ·**22a**, harvested after 10–12 days incubation at 20 °C and flash frozen in liquid nitrogen with cryoprotectant solution containing the mother liquor components in addition to 25% glycerol or 20% PEG400.

A data set of PDEδ·**13b** was taken at the European Synchrotron Radiation Facility (ESRF) with beamline ID 30B. A data set of PDEδ·**22a** was obtained from the Synchrotron X-ray diffraction data using the X10SA beamline at the Swiss Light Source. Both data sets were analyzed and scaled using XDS and XSCALE.^84^ The structures were solved using PHASER^85^ within the PHENIX software suite,^86^ with the structure of 5E80 and 5ML3, respectively, chain A serving as the model for molecular replacement. Refinement was conducted using COOT^87^ for manual refinement and phenix.refine.^88^ Figures were created with Pymol (Version 2.5.4, Schrödinger, LLC). Topology files for both ligands were generated using AceDRG^89^ within the CCP4 suite,^90^ based on their SMILES strings. Restraints for the geometry of the covalent bonds were manually created by modifying the parameter file for phenix.refine. The PDB ID code, data collection, and refinement statistics are presented in Table S5. The crystal structures of PDEδ·**13b** and PDEδ·**22a** were deposited in the Protein Data Bank (PDB) with accession numbers 9RP6 and 9RP7.

### Cell Lines and Cell Culture

All mammalian cells were cultured and maintained in a sterile environment with a humidified atmosphere at 37 °C and 5% CO_2_. Jurkat (ACC282, RRID: CVCL_0065), PA-TU-8902 (ACC179, RRID: CVCL_1845), BxPC3 cells (ACC760, RRID: CVCL_0186), HCT116 (ACC581, RRID: CVCL_0291), LS-174T (ACC759, RRID: CVCL_1384), and A549 (ACC107, RRID: CVCL_0023) were purchased from DSMZ GmbH (Germany). MIA PaCa-2 (CRM-CRL-1420, RRID: CVCL_0428), PANC-1 (ATCC-CRL-1469, RRID: CVCL_0480), SW480 (CCL-228, RRID: CVCL_0546), HT-29 (ATCC-HTB-38, RRID: CVCL_0320), and NCI-H441 (ATCC-CRM-HTB-174, RRID: CVCL_1561) were obtained from ATCC (USA). NCI-H358 (ATCC-CRL-5807, RRID: CVCL_1559) cells were purchased from LGC Standards (Germany). U2OS (CLS-300364, RRID: CVCL_0042) and SK-LU-1 (CLS-300335, RRID: CVCL_0629) cells were obtained from CLS Cell Lines Service GmbH (Germany). HAP1 wild type (Horizon #C631, RRID: CVCL_Y019) and HAP1 PDE6D knockout (Horizon #HZGHC006484c003, RRID: CVCL_XR47) cells were purchased from Horizon (Horizon Discovery, UK). Hs 578T (RRID: CVCL_0332) cells were obtained from the NIH/NCI-DTP. PA-TU-8902, MIA PaCa-2, PANC-1, HCT116, A549, U2OS, and Hs 578Tcells were cultured in Dulbecco’s Modified Eagle’s medium (DMEM, P04-03550, PAN Biotech) supplemented with 10% FBS (Gibco, #10270-106), 1% nonessential amino acids (NEAA, P08-32100, PAN Biotech), and 1 mM sodium pyruvate (P04-43100, PAN Biotech). Jurkat, BxPC3, SW480, LS-174T, NCI-441, and NCI-H358 cells were cultured in RMPI-1640 medium (P04-18047, PAN Biotech) with 10% of FBS (Gibco, #10270-106) and 1% NEAA (P08–32,100, PAN Biotech). HT-29 cells were cultured in MyCoy’s 5A (P04-05500, PAN Biotech) and SK-LU-1 in MEM Eagle (P04–08500, PAN Biotech) supplemented with 10% FBS, respectively. HAP1 wild type and PDE6D knockout cells were cultured in Iscove’s Modified Dulbecco’s Medium (IMDM, P04-20350, PAN Biotech) supplemented with 10% FBS. Mycoplasma tests with the MycoAlert Mycoplasma Detection Kit (Lonza, #LT07-318) were carried out on a regular basis and confirmed that cells were free of contaminations at all times.

### Fluorescence Lifetime Imaging Microscopy

Fluorescence lifetime images were acquired by using a confocal laser-scanning microscope (Leica SP8). For the detection of the donor mCitrine, the sample was excited with a supercontinuum White Light Laser (WLL) with a notch line filter at 470 nm at a 40 MHz repetition frequency. Fluorescence signals were collected through an oil immersion objection and spectrally filtered using a narrow-band emission filter and detected with a photon-counting HyD detector from 519–541 nm. Images were analyzed in real-time on a global scale using the in-built Leica Application Suite X (LAS X) for FLIM, with lifetime calculated with fitting to the in-built model of monoexponential reconvolution.

### Global Proteome and Phosphoproteome Analysis

PA-TU-8902 cells (8 × 10^6^ cells/dish) were seeded in two 15 cm dishes and incubated in a humidified atmosphere at 37 °C and 5% CO_2_ overnight. Cells were then treated with 5 μM of compound **22a** or DMSO, with 0.1% DMSO adjusted for both conditions in fresh medium for 2 h incubated in a humidified atmosphere at 37 °C and 5% CO_2_. All details related to subsequent sample preparation, nanoHPLC–MS/MS analysis, and data evaluation can be found in the Supporting Information—Methods section.

### CETSA in Intact Cells (In-Cell CETSA)

Jurkat cells (adjusted to 9 × 10^6^ cells/flask) were seeded in two T75 tissue culture flasks and treated with 10 μM compound or vehicle (DMSO), with 0.1% DMSO adjusted, for 15 min at 37 °C. Cells were collected and resuspended in cold PBS and further washed three times in cold PBS. The sample from each treatment condition was distributed equally into ten tubes and subjected to heating at different temperatures in the MasterCycler EpGradient S (Eppendorf SE, DE). Afterward, the NP40 alternative was added to a final concentration of 0.4% (v/v), and cells were lysed by five consecutive freeze/thaw cycles. Soluble fractions were separated from denatured proteins by ultracentrifugation at 100,000 g and 4 °C for 25 min (Beckman Coulter Optima MAX-XP, with TLA-120.1 rotor). Supernatants were transferred to new tubes, and equal volumes of each sample were loaded on a self-made 15% Tris-glycine SDS-polyacrylamide gel and subjected to immunoblot analysis with gel electrophoresis in Tris-glycine SDS running buffer (25 mM Tris, 0.2 M glycine, 0.1% SDS) at 90 V for 15 min stacking, followed by 120 V until sufficient separation was visualized by the PageRuler prestained protein ladder. Proteins were transferred onto a polyvinylidene difluoride (PVDF) membrane (Thermo Fisher Scientific, #88518, 0.45 μm) using a wet-tank blotting system (Bio-Rad) with precooled transfer buffer (25 mM Tris, 0.2 M glycine, 10% methanol) at 100 V for 30 min. Membranes were blocked with Intercept Blocking Buffer (LI-COR Biosciences, #927-70001) for 1 h at room temperature, incubated with the primary antibody anti-PDEδ (Invitrogen, PA5-22008, RRID: AB_11154288, 1:500) overnight at 4 °C in blocking buffer, then washed with PBS-T (PBS with 0.1% Tween-20) five times and incubated with IRDye 800CW-conjugated secondary antibody (LI-COR Biosciences, #926-32210, RRID: AB_621842, 1:5000) in blocking buffer for 1 h at room temperature with protection from light. After washing with PBS-T five times, secondary antibody-incubated membranes were imaged with the ChemiDocMP Imaging System (BIO-RAD Laboratories) directly with IRDye800CW channel for visualization of bands. Relative band intensities were quantified using Image Lab (BIO-RAD) and normalized to the intensities of the bands at 37 °C.

### SDS-PAGE and Immunoblotting

PA-TU-8902 cells (8 × 10^6^ cells/dish) were seeded in two 15 cm dishes and incubated in a humidified atmosphere at 37 °C and 5% CO_2_ overnight. Cells were then treated with 5 μM of compound **22a** for the indicated time points. Cells were washed and harvested by scraping. Cells were lysed by resuspension in 100 μL of sodium dodecyl sulfate (SDS) lysis buffer and sonicated in the Bioruptor (Diagenode Inc.). SDS-lysed samples were heated at 95 °C for 10 min and quantified via the DC assay. Samples were diluted in Laemmli buffer, heated at 95 °C for 10 min, and loaded on an SDS polyacrylamide gel (5% stacking gel, 10% separating gel). The Mini-PROTEAN Tetra Cell immunoblotting system (BioRad Laboratories, Inc.) was used for electrophoresis and transfer. Gels were transferred onto a PVDF membrane (Merck Millipore) via Trans-Blot Turbo Semi Dry Blotting at 25 V for 7 min. Membranes were washed in 1x TBS-T and blocked in 5% BSA in TBS-T for 1 h. For immunostaining, the membranes were incubated overnight with primary antibodies diluted in 5% BSA at 4 °C (Anti-pERK1/2, CST9107, 1:1000; Anti-ERK1/2, Abcam36991, 1:1000; Anti-pS6, CST4856, 1:1000; Anti-S6, CST2317, 1:100). After three washes in 1x TBS-T for 10 min each, the membranes were incubated with Horseradish Peroxidase (HRP) conjugated secondary antibody diluted in 5% BSA at RT for 1 h, following another three washes. ECL Prime Blotting Detection Reagent (Merck Millipore) was used for protein detection using the ChemiDoc Imaging System (BioRad Laboratories Inc.). Signal intensities from SDS-PAGE bands were quantified by using FIJI software. Square ROI were defined around each band, and background correction was performed by subtracting the mean intensity of an equivalent ROI positioned above each band. For phosphorylated proteins, band intensities were normalized to their corresponding total protein levels. 22a treatment data were presented in percentages relative to the untreated control lane (set as 100%).

### Real-Time Live-Cell Analysis by Incucyte

Real-time live-cell analysis monitoring cell growth was performed with an IncuCyte ZOOM and Incucyte S3 live-cell analysis instrument (Sartoris AG, Germany). For each adherent cell line, 5 × 10^3^ to 1.2× 10^4^ cells were seeded in each well of the 96-well plates in 100 μL of cell culture medium and incubated overnight in a humidified incubator at 37 °C with 5% CO_2_ before replacement of fresh medium and addition of respective compounds, with 0.5% DMSO adjusted for all conditions. Plates were inserted into the Incucyte S3 live-cell analysis instrument and incubated in a humidified incubator at 37 °C with 5% CO_2_. Images of cells (2 images/well) were taken with the phase channel every 2 h until full confluency was reached in the DMSO controls. Images were analyzed with the basic analyzer inbuilt in the Incucyte Zoom (2018A) and IncuCyte S3 (2019B, Rev2) analysis software with a confluence mask calculating the percent phase area confluence of cells as an indicator of cell growth. To display dose–response curves, percent phase area confluence at 72 h of treatment was normalized to the DMSO control, plotted, and fitted with GraphPad Prism 9.2 (GraphPad software, USA) using a four-parameter variable slope nonlinear regression curve fit to calculate cellular IC_50_ values. For calculation of growth rate, percent phase area confluence was plotted against time for each respective condition in GraphPad Prism, and the area below the curve was integrated for 96 h after administration of the first dose of drugs and normalized to the DMSO control.

### Kras^LSL.G12D/wt^;Trp^53fl/fl^ (KP) Mouse Model Study

All details related to the in vivo mouse model study can be found in the Supporting Information—Methods section. The mouse experiments were licensed by the State Agency for Food and Consumer Protection (LAVE) under license 81-02.04.2020.A281. Breeding of mouse lines was permitted by the State Agency for Food and Consumer Protection (LAVE) under licenses 81-02.04.2019.A009 and 2024-225.

## Supplementary Material





## Data Availability

All unique/stable materials and reagents generated in this study are available from the lead contact with a completed material transfer agreement. The crystal structures of covalent PDEδ adducts modified by compounds **13b** and **22a** (Deltafluorine) were deposited in the Protein Data Bank (PDB) with the accession numbers 9RP6 and 9RP7.

## Notes

Structures of selected PDEδ inhibitors and their cocrystal structures with PDEδ describing the same figure; supplementing design strategy of compound development; structural development of benzyl halide electrophiles; binding affinities of **13a** and **22a** to PDEδ; stability of **13b** and **22a** in aqueous buffers and in the presence of GSH; global proteome profiling of **22a**; phosphoproteome profiling of **22a**; quantified immunoblots of phosphorylation of ERK1/2 and S6 in PA-TU-8902 cells; cellular profiling of Deltafluorine (**22a**), including cell painting, single-dose treatment in PA-TU-8902 cells, and HAP1 wildtype versus PDEδ knockout cells; cellular dose–response curves of Deltafluorine **22a**; PK/PD studies of Deltafluorine **22a**; pretreatment μCT data; uncropped immunoblots of CETSA; mass spectrometry analysis of covalent peptide adducts of PDEδ; Reactome pathway analysis of global proteome upon **22a** treatment; Reactome pathway analysis of upregulated kinases upon **22a** treatment; Reactome pathway analysis of downregulated kinases upon **22a** treatment; X-ray crystallography data collection and refinement statistics; detailed methods for mass spectrometry analysis of covalent peptide adducts of PDEδ after Glu-C digestion, global proteome and phosphoproteome analysis, and Kras^LSL.G12D/wt^;Trp^53fl/fl^ (KP) Mouse Model Study; detailed description, methods, and analysis of compound synthesis; material list for this study (PDF)
SMILES and activity data of all investigated compounds (CSV)

## Abbreviations

Akt: protein kinase B
Arl: ADP ribosylation factor-like GTPase
HLA-C: human leukocyte antigen-C
KP: Kras^LSL.G12D/wt^;Trp53^fl/fl^
mTOR: mammalian target of rapamycin
PDEδ: phosphodiesterase (6) delta
PTB: phosphotyrosine-binding domain
Rab: Ras-related protein
Raf: rapidly accelerated fibrosarcoma protein
Rheb: Ras homologue enriched in brain
Rho: Ras homologue
S6: ribosomal protein S6
TCI: targeted covalent inhibitor
TMR: tetramethylrhodamine
Trp53: transformation-related protein 53
μCT: microcomputed tomography
