# Supplementary material for: Targeting a Glutamic Acid in PDEδ with Fluoromethyl-Aryl Electrophiles Impairs K‑Ras Signaling
Source: J Med Chem. 2026 Jan 7;69(2):964–81. doi: 10.1021/acs.jmedchem.5c02082 (PMC12833852; doi:10.1021/acs.jmedchem.5c02082)
Supplement: Supplementary file 1 [file jm5c02082_si_001.pdf]

**Supporting Information**

# Targeting a Glutamic Acid in PDEδ with Fluoromethyl-Aryl Electrophiles Impairs K-Ras Signaling

Ruirui Zhang[1,§], Maxim A. Huetzen[2,3,4,5,§], Aylin Binici[1,6,§], Pablo Martín-Gago[1,§], Raphael Gasper[7], Elena Rudashevskaya[1], Jie Liu[1], Chinta Nagaraju[1], Elena S. Reckzeh[1], Alana S.T. Stuedle[2,3,4,5], Ann-Sophie Hopff[2,3,4,5], Andrea Mesaros[8], Anke Unger[9], Melanie Thelen[2,3,4,5], Petra Janning[1], H. Christian Reinhardt[10], Slava Ziegler[1], Ron D. Jachimowicz[2,3,4,5,*], Herbert Waldmann[1,6,*]

[1] Max Planck Institute of Molecular Physiology, Department of Chemical Biology, Otto-Hahn-Street 11, 44227 Dortmund, Germany

[2] Max Planck Research Group Mechanisms of DNA Repair, Max Planck Institute for Biology of Ageing, 50931 Cologne, Germany

[3] Department I of Internal Medicine, Center for Integrated Oncology Aachen Bonn Cologne Duesseldorf (CIO ABCD), University of Cologne; 50931 Cologne, Germany

[4] Cologne Excellence Cluster on Cellular Stress Response in Aging-Associated Diseases, University of Cologne; 50931 Cologne, Germany

[5] Center for Molecular Medicine Cologne, University of Cologne; 50931 Cologne, Germany

[6] Technical University Dortmund, Faculty of Chemistry and Chemical Biology, Otto-Hahn-Street 6, 44221 Dortmund, Germany

[7] Max Planck Institute of Molecular Physiology, Crystallography and Biophysics Facility, Otto-Hahn-Street 11, 44227 Dortmund, Germany

[8] Phenotyping Core Facility, Max Planck Institute for Biology of Ageing, 50931 Cologne, Germany

[9] Lead Discovery Center GmbH, Otto-Hahn-Str. 15 D-44227 Dortmund, Germany

[10] Department of Hematology and Stem Cell Transplantation, University Hospital Essen, West German Cancer Center, German Cancer Consortium Partner Site Essen, Center for Molecular Biotechnology, University of Duisburg-Essen, Hufelandstraße 55, 45147 Essen, Germany

[§] These authors contributed equally to this work.

[*] Corresponding authors, email: Herbert.waldmann@mpi-dortmund.mpg.de and rjachimowicz@age.mpg.de

**Table of Contents**

**1. Supplementary Figures and Tables** .......... **2**

**Supplementary Figure 1.** Selected PDEδ Inhibitors and Co-crystal Structures with PDEδ. .......... 2

**Supplementary Figure 2.** Structural Development of Benzyl Halide Electrophiles. .......... 3

**Supplementary Figure 3.** Binding Affinities of **13a** and **22a** to PDEδ. .......... 5

**Supplementary Figure 4.** Stability of **13b** and **22a** in Aqueous Buffers and in Presence of GSH. .......... 6

**Supplementary Figure 5a-d.** Overview of Pathway Enrichment for Global Proteome Profiling of **22a**. .......... 7

**Supplementary Figure 5e-f.** Overview of Pathway Enrichment for Phosphoproteome Profiling of **22a**. .......... 9

**Supplementary Figure 6.** Quantified Immunoblots Related to Figure 4c. .......... 10

**Supplementary Figure 7.** Cellular Profiling of Deltafluorine (**22a**). .......... 11

**Supplementary Figure 8.** Cellular Dose-response Curves of Deltafluorine (**22a**). .......... 13

**Supplementary Figure 9.** PK/PD Studies of Deltafluorine (**22a**). .......... 15

**Supplementary Figure 10.** Pre-treatment μCT data show similar tumor burden in both groups. .......... 17

**Supplementary Figure 11.** Uncropped Immunoblots Related to Figure 5a. .......... 18

**Supplementary Table 1.** Mass Spectrometry Analysis of Covalent Peptide Adducts of PDEδ. .......... 19

**Supplementary Table 2.** Reactome Pathway Analysis of Global Proteome upon **22a** Treatment. .......... 20

**Supplementary Table 3.** Reactome Pathway Analysis of Upregulated Kinases upon **22a** Treatment. .......... 21

**Supplementary Table 4.** Reactome Pathway Analysis of Downregulated Kinases upon **22a** Treatment. .......... 22

**Supplementary Table 5.** X-ray Crystallography Data Collection and Refinement Statistics. .......... 23

**2. Methods** .......... **24**

Mass Spectrometry Analysis of Covalent Peptide Adducts of PDEδ after Glu-C Digestion .......... 24

Global Proteome and Phosphoproteome Analysis .......... 25

*Kras*$^{LSL.G12D/wt}$; *Trp*$^{53fl/fl}$ *(KP)* Mouse Model Study .......... 28

**3. Chemical Synthesis** .......... **30**

General Information .......... 30

Compound Characterization .......... 33

Spectral Characterization for Representative Compounds .......... 47

**4. Material List** .......... **59**

**5. References** .......... **62**

## 1. Supplementary Figures and Tables

**Supplementary Figure 1.** Selected PDEδ Inhibitors and Co-crystal Structures with PDEδ.

Selected structures of PDEδ inhibitors

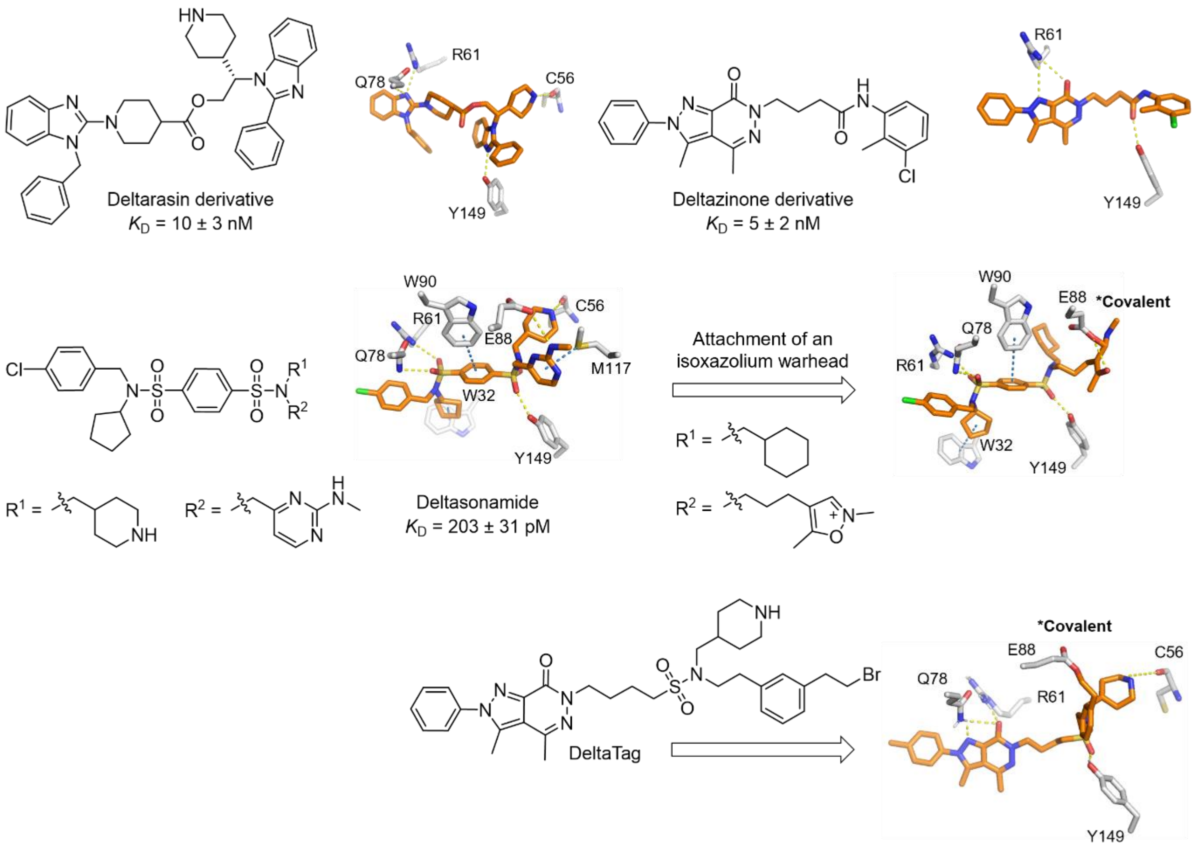


Structures of Deltarasin derivative, Deltazinone derivative, Deltasonamide, covalent Deltasonamide derivative with an isoxazolium warhead and covalent DeltaTag are shown with key interactions in the PDEδ prenyl binding pocket (PDB codes: 4JVF, 5E80, 5ML3, 5NAL and 9HMD,[1-5]). Key interacting amino acid side chains in the binding pocket of PDEδ are shown as ball-and-stick representation. Hydrogen bonds are shown as yellow dotted line. Red spheres are bound water molecules.

**Supplementary Figure 2.** Structural Development of Benzyl Halide Electrophiles.

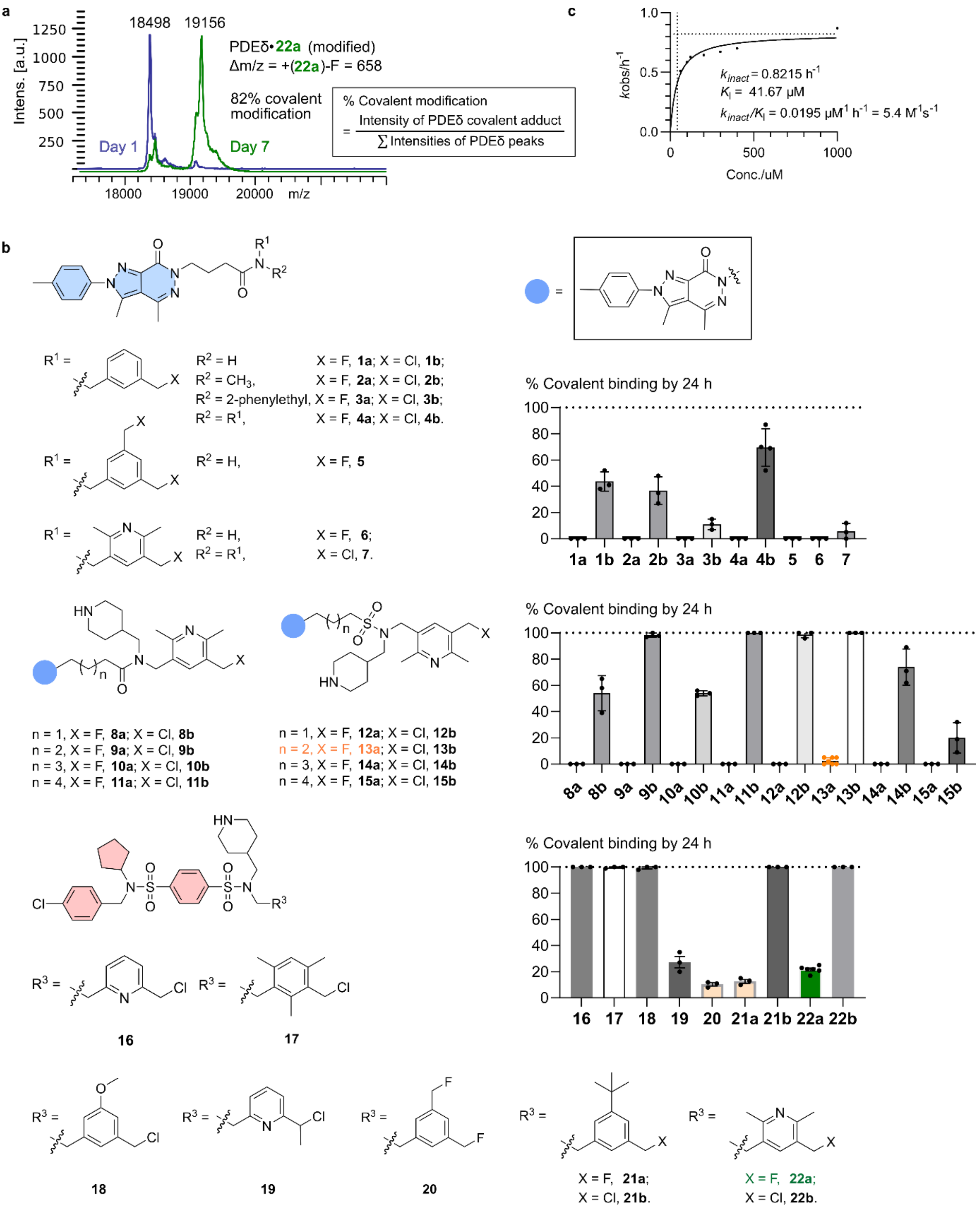


**a**, Representative positive ion-mode MALDI-TOP mass spectra of PDEδ (20 μM) in the presence or absence of **22a** (60 μM, 0.6% DMSO) after 1 and 7 days of incubation in HEPES buffer (20 mM HEPES, 150 mM NaCl, pH = 7.5) at 37 °C. Percentages of covalent adduct formation were estimated by the relative intensity of the respective peaks in MALDI spectra. **b**, Compound structures and their

percent [%] covalent modification at 24 hours by MALDI mass spectrometry. PDEδ (20 µM) was incubated with compounds (60 µM) in HEPES buffer (20 mM HEPES, 150 mM NaCl, pH = 7.5) at 37 °C for 24 hours before analysis by MALDI. Percentages of covalent adduct formation were estimated by the relative intensity of the respective peaks in MALDI spectra. Data are presented as mean ± standard error of the mean (n = 3 with the exceptions of n = 4 for **4b**, n = 6 for **13a** and **22a**). **c**, Second-order PDEδ labelling kinetics by compound **22a**. PDEδ (20 µM) was incubated with compound **22a** (60 µM – 1 mM) in HEPES buffer (20 mM HEPES, 150 mM NaCl, pH = 7.5) at 37 °C. Apparent pseudo-first order $k_{obs}$ was estimated by percentages of covalent adduct formation at 30 h by Velos Pro HPLC-MS. Plot of $k_{obs}$ ($h^{-1}$) against concentration (µM) was fitted by GraphPad Prism Michaelis-Menten model and separate values for $k_{inact}$ and $K_I$ were obtained to calculate apparent second-order kinetic $k_{inact}/K_I$.

**Supplementary Figure 3.** Binding Affinities of **13a** and **22a** to PDEδ.

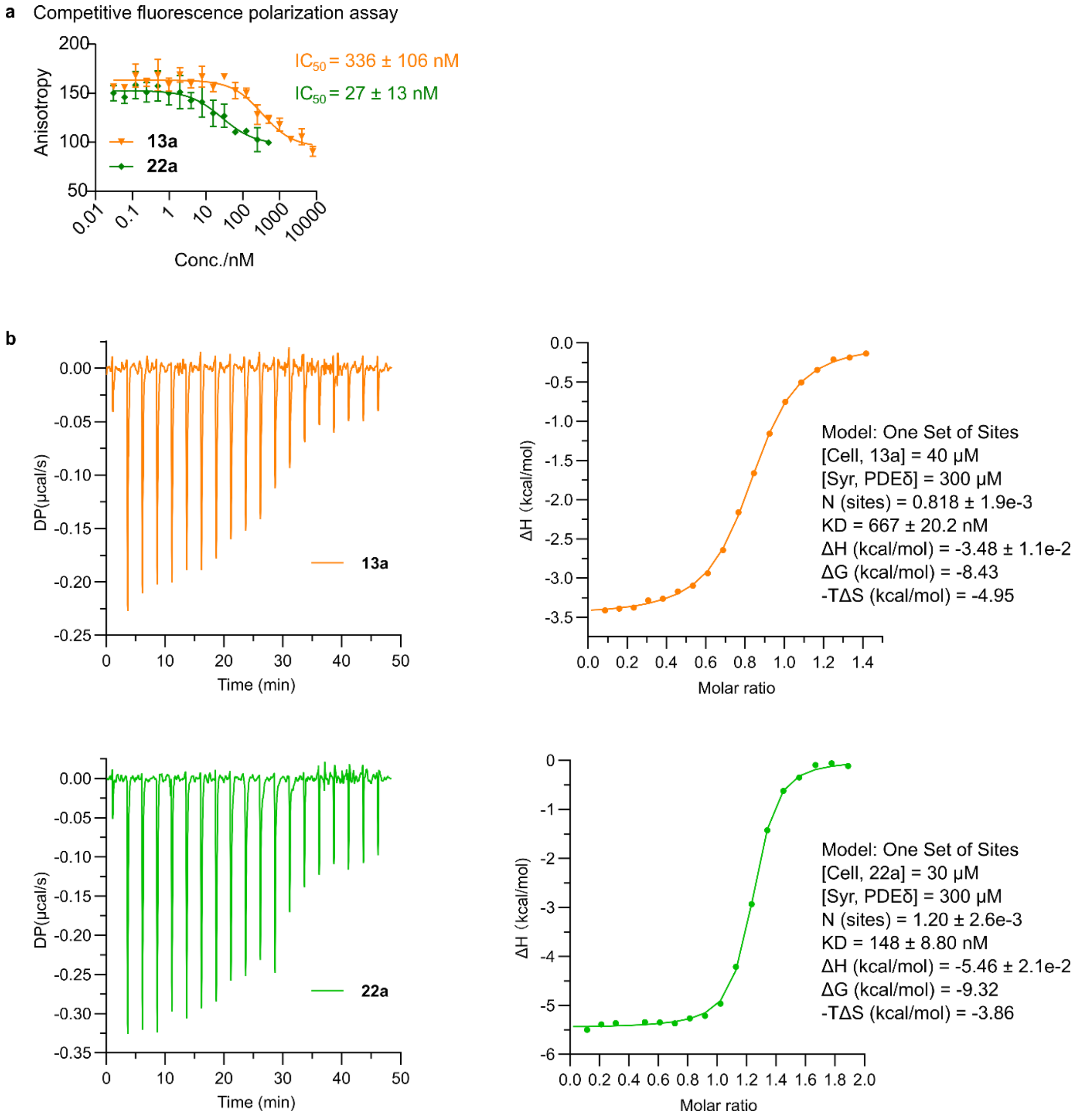


**a**, Competitive fluorescence polarization assay for $IC_{50}$ values of **13a** and **22a** by incubating the compounds with PDEδ (40 nM) and FA probe (Fluorescein-PEG-Atorvastatin probe, 24 nM) at room temperature overnight. Data are presented as mean ± standard deviation (n = 3). **b**, Isothermal titration calorimetry (ITC) measurement for evaluating the binding affinity of **13a** and **22a** to PDEδ at 25 °C. All curves were fit using a one-site model with defined compound and PDEδ concentrations. Left plots show baseline corrected data, while right plots show integrated binding isotherms versus compound to PDEδ ratio. PDEδ protein (300 μM) was titrated into the stirred sample cell containing **13a** (40 μM) or **22a** (30 μM). Representative data for three independent titrations (n = 3).

**Supplementary Figure 4.** Stability of **13b** and **22a** in Aqueous Buffers and in Presence of GSH.

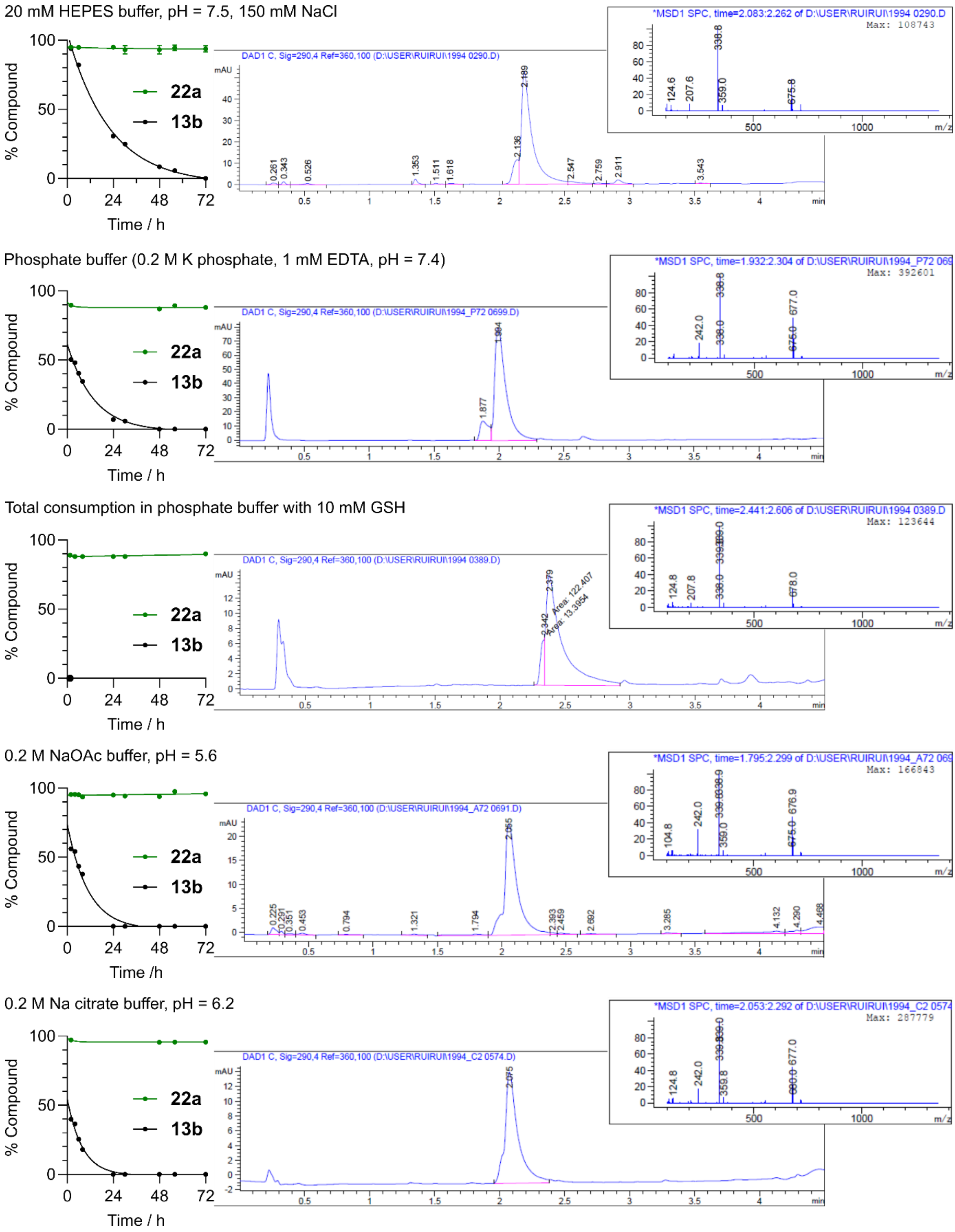


Initial compound concentration = 1 mM, incubated at 37 °C, 600 rpm, protected from light. Comparative stability data between **13b** and **22a** were shown in the left panels. HPLC spectra and MS for **22a** at 72 h were shown for each condition to the right panels.

**Supplementary Figure 5a-d.** Overview of Pathway Enrichment for Global Proteome Profiling of **22a**.

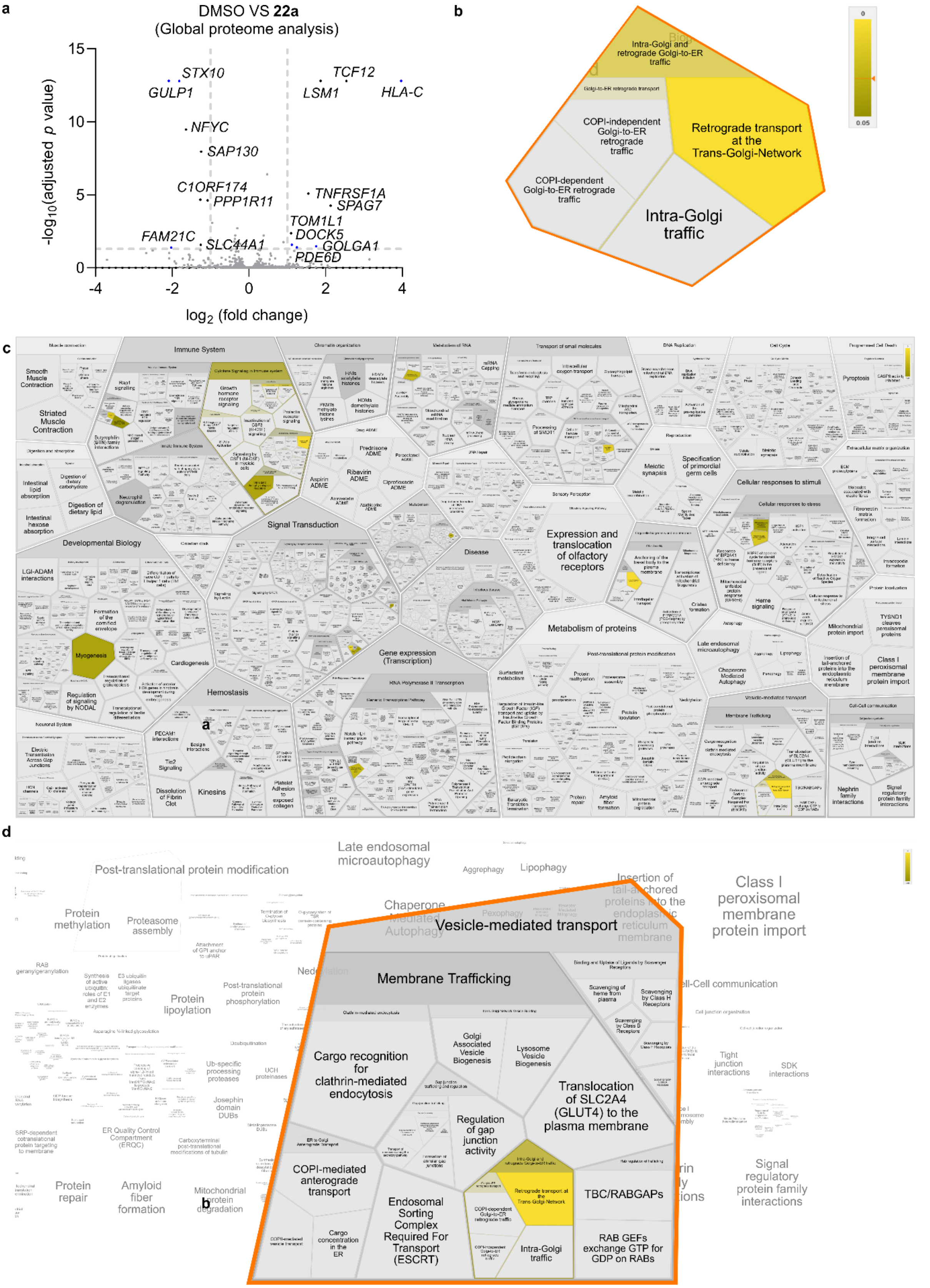

**a**, Global proteome analysis. Volcano plot with -$log_{10}$ (adjusted *p*-value) plotted against $log_2$fold change between DMSO treated and compound treated conditions is shown. Significant hits (adjusted *p*-value $\leq 0.05$, $log_2$fold change $\geq 1$ or $\leq -1$) are labeled with their gene names. Protein hits involved in the endosomal pathway and membrane trafficking of protein cargos are highlighted with blue dots. **b**, Reactome pathway overrepresentation[6] of significant hits from global proteome profiling, with Voronoi visualization zooming into membrane trafficking across Golgi-Endoplasmic Reticulum (ER) network. The scale of color intensity is an indication of *p*-value of pathway overrepresentation. **c** and **d**, Overview of Voronoi visualization for Reactome pathway overrepresentation[6] of significant hits from global proteome profiling, uncropped images.

**Supplementary Figure 5e-f.** Overview of Pathway Enrichment for Phosphoproteome Profiling of **22a**.

e

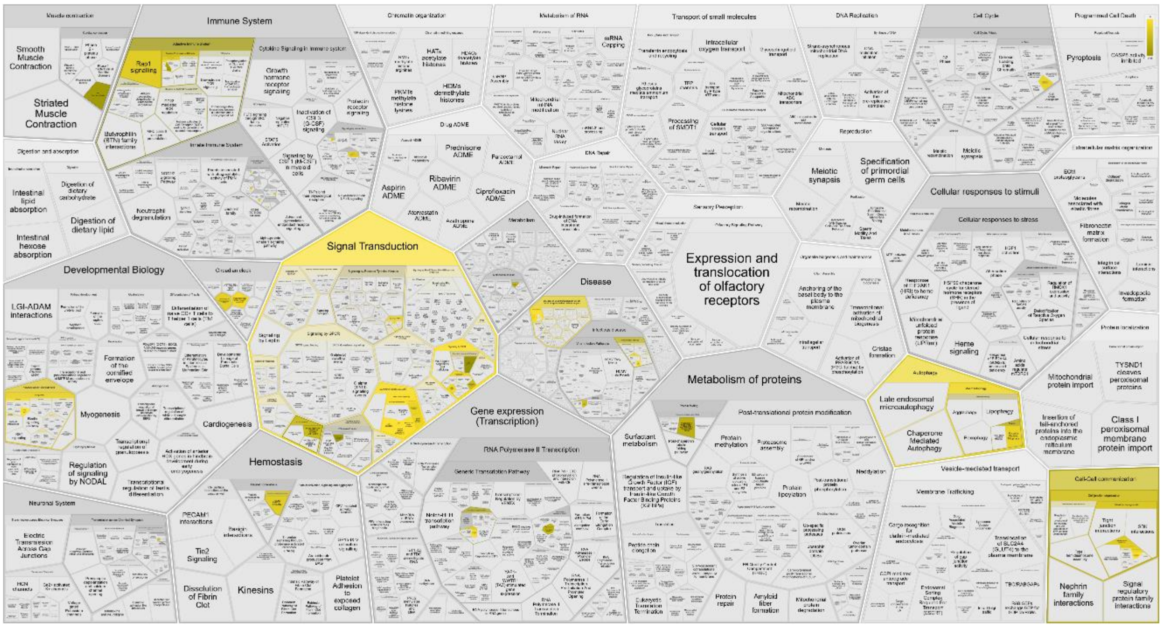


f

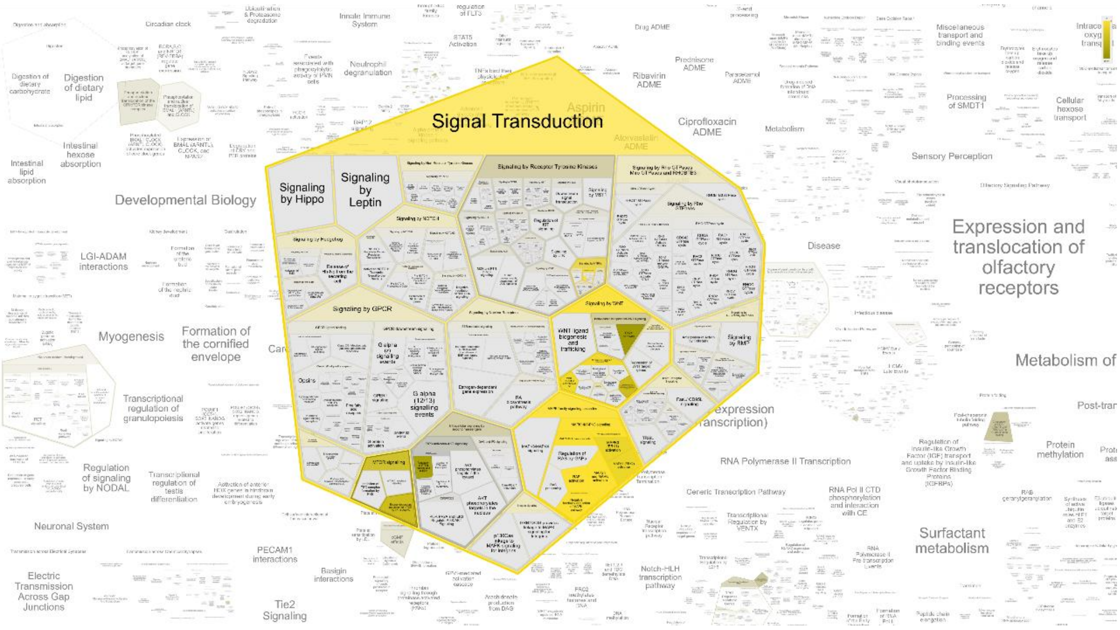


**e** and **f**, Overview of Voronoi visualization for Reactome pathway overrepresentation[6] of significantly suppressed hits from kinase-substrate enrichment analysis[7-9] of phosphoproteome profiling, uncropped images.

**Supplementary Figure 6.** Quantified Immunoblots Related to Figure 4c.

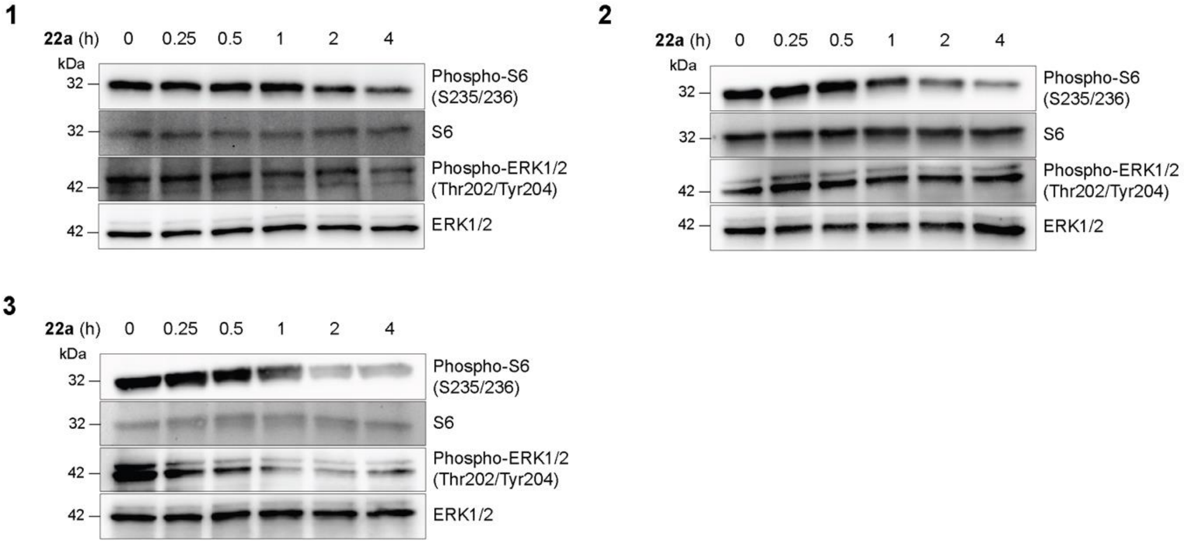


Immunoblot analysis examining phosphorylation of ERK1/2 at Thr202/Tyr204 and phosphorylation of S6 at S235/236 in PA-TU-8902 cells upon a time-course treatment with 5 µM Deltafluorine. Data represents 3 individual experiments (N = 3).

**Supplementary Figure 7.** Cellular Profiling of Deltafluorine (**22a**).

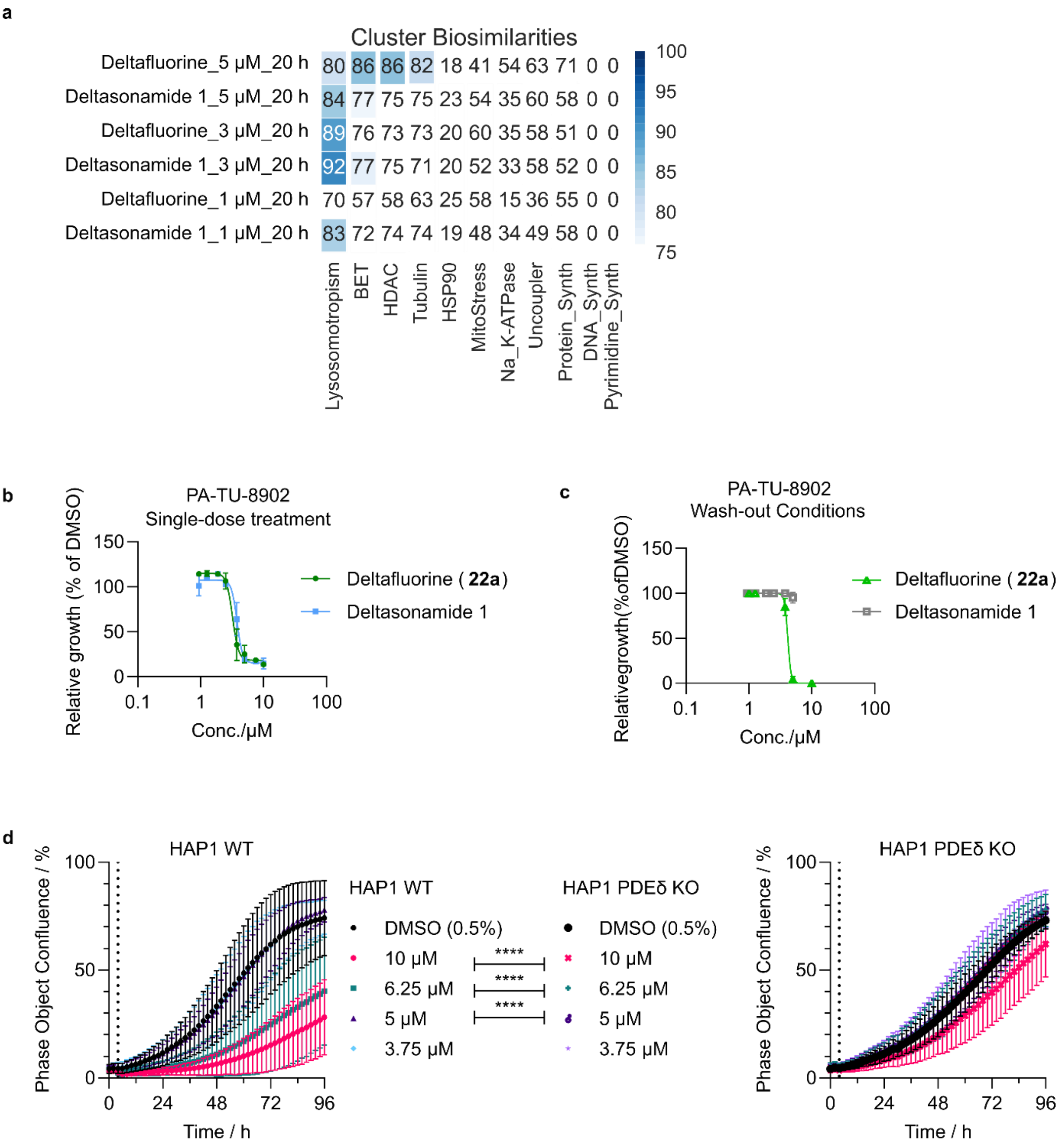


**a,** Morphological cellular profiling of Deltafluorine (**22a**) in comparison to Deltasonamide 1. The Cell Painting assay (CPA)[10] revealed that both Deltafluorine (**22a**) and Deltasonamide 1 showed high similarity to the previously identified lysosomotropic profile[11,12] at non-toxic doses. **b**, Single-dose treatment of Deltafluorine (**22a**) in PA-TU-8902 cells in comparison to Deltasonamide 1. Relative cell growth after 72 hours of compound treatment was normalized to DMSO control. Data were plotted as mean ± standard deviation, representative of two biological replicates (n = 2). Curves were fitted in GraphPad Prism with nonlinear fitting model [inhibitor] vs response with variable slope (four parameters) for cellular $IC_{50}$ determination. 72 h $IC_{50}$ of Deltafluorine = 3.1 ± 0.1 μM. 72 h $IC_{50}$ of

Deltasonamide 1 = 3.8 ± 0.1 µM in PA-TU-8902. **c**, 72 h $IC_{50}$ comparison for Deltafluorine and Deltasonamide 1 under wash-out regime. Relative cell growth after 72 hours of first compound treatment was normalized to the respective DMSO control. Data were plotted as mean ± standard deviation, representative of three biological replicates (n = 3 for all concentrations; n = 5 for 5 µM). Curves were fitted in GraphPad Prism with nonlinear fitting model [inhibitor] vs response with variable slope (four parameters) for cellular $IC_{50}$ determination. 72 h $IC_{50}$ of single-dose Deltafluorine = 3.1 ± 0.1 µM. 72 h $IC_{50}$ of Deltafluorine with wash-out (after three doses of 4 h treatment) = 4.2 ± 0.1 µM in PA-TU-8902. **d**, Cell proliferation with dose-dependent treatment of Deltafluorine (**22a**) monitored with real-time live-cell imaging by Incucyte. Cells were treated with the compound or vehicle for 4 hours and compound removal by washing with fresh culture medium are marked with grey dotted lines. Data of percent (%) phase object confluence were plotted as mean ± standard deviation (representative of biological replicates n = 3). Paired *t*-test at each concentration between two cell lines with phase object confluence normalized to DMSO control at each time point, two-tailed *p*-value, ****$p$-value < 0.0001.

**Supplementary Figure 8.** Cellular Dose-response Curves of Deltafluorine (**22a**).

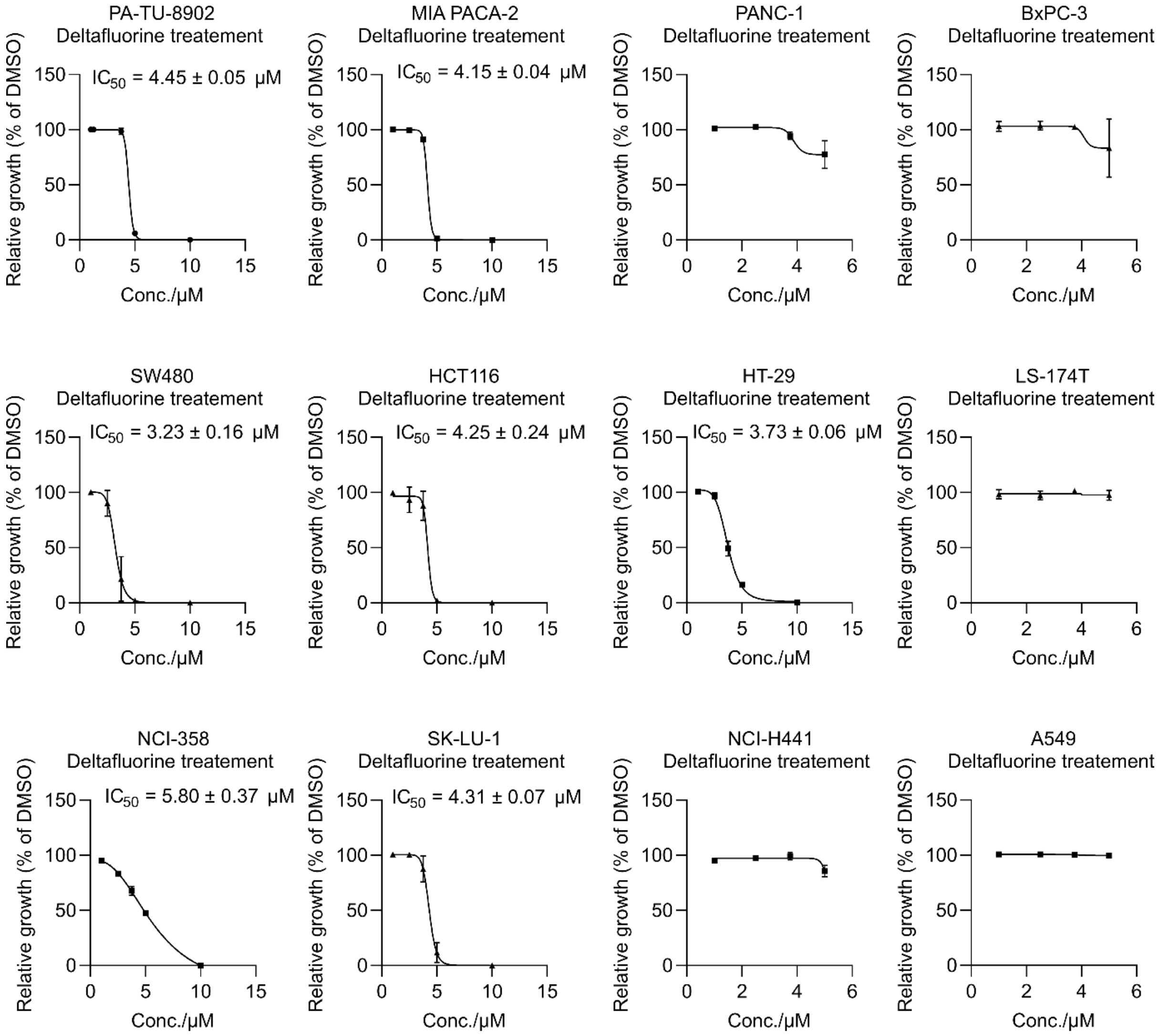


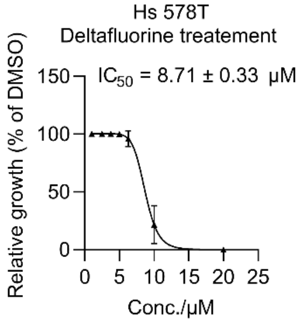


| | Cell line | IC$_{50}$ values / μM |
|---|---|---|
| *KRAS* mutant | PA-TU-8902 | 4.45 ± 0.05 |
| | MIA PACA-2 | 4.15 ± 0.04 |
| | SW480 | 3.23 ± 0.16 |
| | HCT116 | 4.25 ± 0.24 |
| | NCI-H358 | 5.80 ± 0.37 |
| | SK-LU-1 | 4.31 ± 0.07 |
| *HRAS* mutant | Hs 578T | 8.71 ± 0.33 |
| *BRAF* mutant | HT-29 | 3.73 ± 0.06 |

Relative cell growth after 96 hours of first compound treatment (after four doses of 4 h treatment) was normalized to the respective DMSO control. Data were plotted as mean ± standard deviation, representative of three biological replicates (n = 3 for all cell lines). Curves were fitted in GraphPad

Prism with nonlinear fitting model [inhibitor] vs response with variable slope (four parameters) for cellular $IC_{50}$ determination. Comparison of $IC_{50}$ values were made among KRAS mutant cell lines, *HRAS* mutant and *BRAF* mutant cell lines.

**Supplementary Figure 9.** PK/PD Studies of Deltafluorine (**22a**).

a b

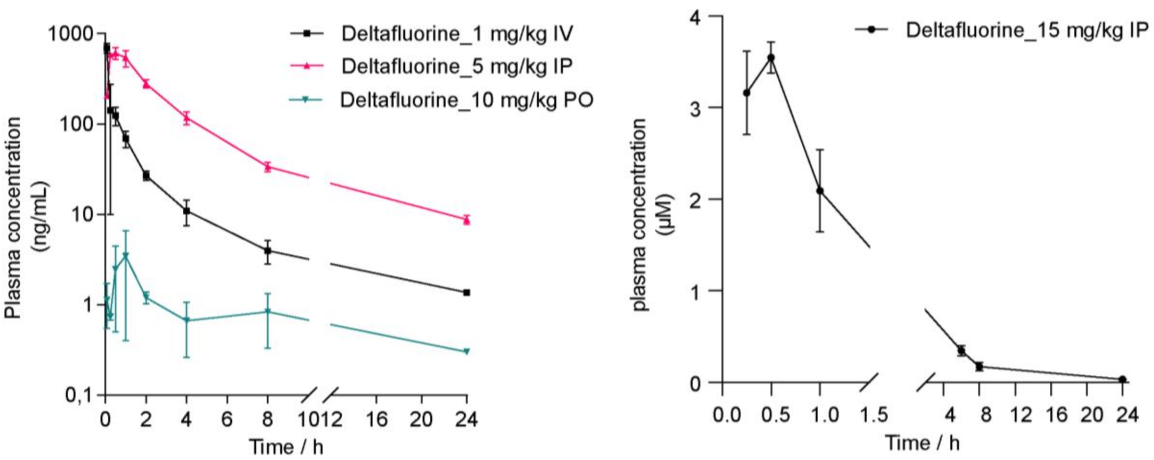


c

| | 1 mg/kg IV | 5 mg/kg IP | 10 mg/kg PO | 15 mg/kg IP |
|---|---|---|---|---|
| **Vehicle** | 20% (v/v) DMSO and 80% (v/v) PEG400 | 5% (v/v) DMSO, 30% (v/v) HP -*β*-CD and 0.5% (v/v) HPMC | | |
| **$C_{max}$ [ng/ml]** | - | 608 | 4 | 2402 |
| **$t_{max}$ [h]** | - | 0.5 | 1.0 | 0.5 |
| **$t_{1/2}$ [h]** | 7.3 | 6.0 | 12.5 | 5.7 |
| **V/f [L/kg]** | - | 21.1 | 7674.0 | 16.2 |
| **CL/f [L/(h*kg)]** | - | 2.5 | 474.2 | 1.9 |
| **F [%]** | - | 114 | 0.7 | - |

d

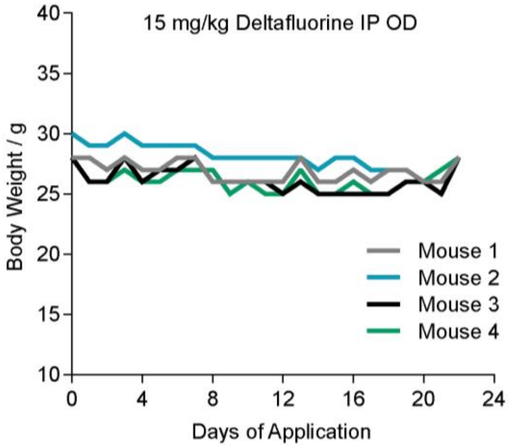


**a**, Comparison of the dosage routes of Deltafluorine for *in vivo* studies. For intravenous (IV) administration, a vehicle consisting of 20% (v/v) DMSO and 80% (v/v) PEG400 was used. In contrast, for intraperitoneal (IP) and oral (PO) administrations, a solution of 5% (v/v) DMSO, 30% (v/v) 2-hydroxypropyl-beta-cyclodextrin (HP-*β*-CD), and 0.5% (v/v) hydroxypropylmethylcellulose (HPMC)

in water was employed. **b**, Plasma concentration of the Deltafluorine route used in *in vivo* studies. **c**, PK parameters for Deltafluorine treatment across different dosage routes and treatment concentrations. $C_{max}$ [ng/ml] = maximum compound concentration observed in plasma; $t_{max}$ [h] = time to achieve maximum compound concentration in plasma; $t_{1/2}$ [h] = compound half-life in plasma; V/f [L/kg] = volume of distribution; CL/f [L/(h*kg)]: total body clearance, and F [%] = bioavailability. **d**, Effects of a 22-day daily treatment with 15 mg/kg Deltafluorine administered via IP on mice, monitored by tracking body weight.

**Supplementary Figure 10.** Pre-treatment µCT data show similar tumor burden in both groups.

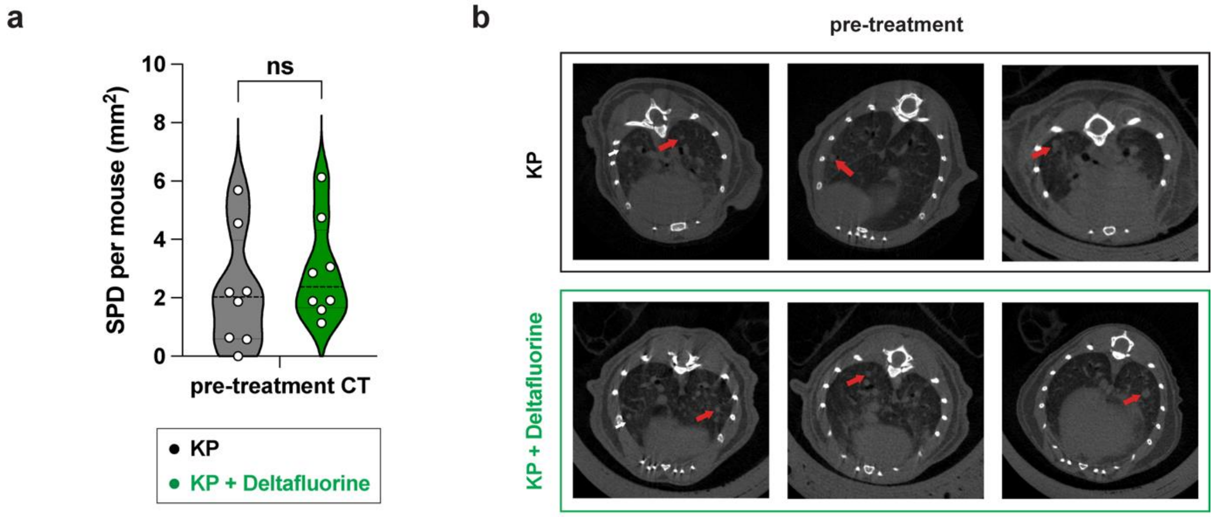


(a) Cumulative tumor burden per mouse (sum of the products of diameters) at pre-treatment µCT imaging. (b) Exemplary transversal µCT images from the center of lungs pre-treatment of three mice per group. Identified tumors are indicated with red arrows.

**Supplementary Figure 11.** Uncropped Immunoblots Related to Figure 5a.

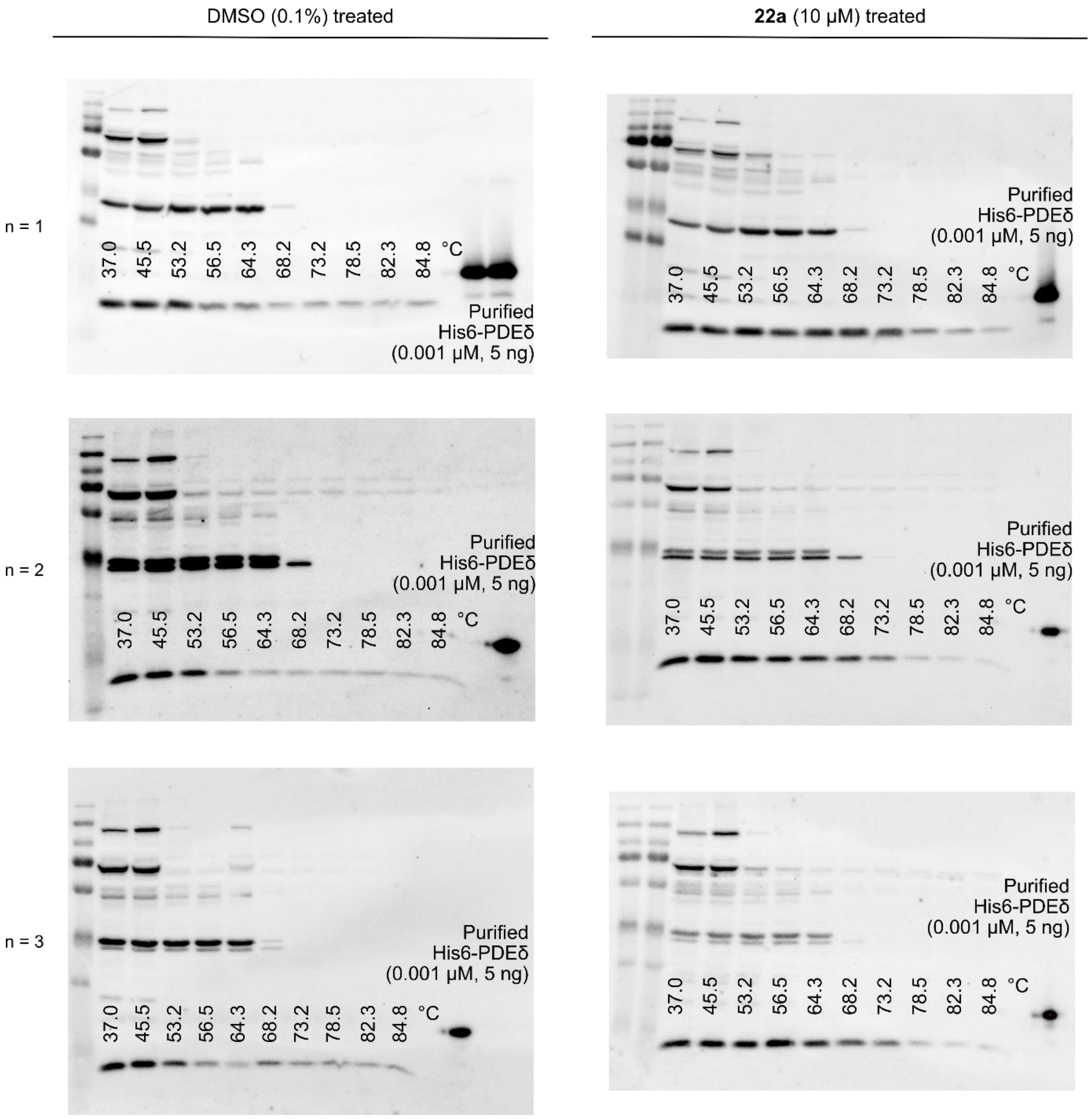

**Supplementary Table 1.** Mass Spectrometry Analysis of Covalent Peptide Adducts of PDEδ.

| Site modified | | Peptides identified with localisation probability[a] | Score[b] | PEP[c] | Intensity[d] | Ratio modified /base[e] |
|---|---|---|---|---|---|---|
| PDEδ·**9b** | | | | | | |
| R1 | E88 | QKVYFKGQCLE(0.868)E(0.132) | 67.385 | $1.77 \times 10^{-4}$ | $2.19 \times 10^{7}$ | 3.19 |
| R2 | E88 | QKVYFKGQCLE(0.807)E(0.193) | 59.265 | $7.22 \times 10^{-4}$ | $4.75 \times 10^{7}$ | 0.480 |
| | E89 | QKVYFKGQCLE(0.139)E(0.861) | 87.618 | $3.24 \times 10^{-6}$ | $1.19 \times 10^{7}$ | 0.120 |
| R3 | E88 | QKVYFKGQCLE(0.863)E(0.137) | 76.332 | $1.21 \times 10^{-5}$ | $2.58 \times 10^{7}$ | 0.935 |
| | E89 | QKVYFKGQCLE(0.135)E(0.865) | 97.214 | $9.08 \times 10^{-9}$ | $5.18 \times 10^{6}$ | 0.188 |
| PDEδ·**11b** | | | | | | |
| R1 | E88/ E89 | QKVYFKGQCLE(0.5)E(0.5) | 76.533 | $3.68 \times 10^{-5}$ | $9.56 \times 10^{6}$ | 0.704 |
| | E114 /S115 | AAPE(0.5)S(0.5)QMMPAS(0.006)V LTGNVIIE | 67.995 | $6.46 \times 10^{-6}$ | $1.07 \times 10^{7}$ | 1.20 |
| R2 | E88 | QKVYFKGQCLE(0.834)E(0.166) | 50.806 | $1.25 \times 10^{-4}$ | $7.65 \times 10^{7}$ | 0.621 |
| | E88/ E89 | QKVYFKGQCLE(0.54)E(0.46) | 74.326 | $4.23 \times 10^{-5}$ | $1.74 \times 10^{7}$ | 0.141 |
| | E110 | FGFVIPNS(0.001)T(0.001)NT(0.001 )WQS(0.035)LIE(0.79)AAPE(0.172) | 58.672 | $2.58 \times 10^{-5}$ | $2.85 \times 10^{7}$ | 0.0771 |
| | E114 /S115 | AAPE(0.5)S(0.5)QMMPAS(0.006)V LTGNVIIE | 106.94 | $1.54 \times 10^{-13}$ | - | - |
| R3 | E88 | QKVYFKGQCLE(0.907)E(0.093) | 58.676 | $3.78 \times 10^{-4}$ | $4.46 \times 10^{7}$ | 0.708 |
| | E89 | QKVYFKGQCLE(0.019)E(0.981) | 102.4 | $2.28 \times 10^{-7}$ | $8.91 \times 10^{6}$ | 0.141 |
| | E114 | AAPE(0.886)S(0.113)QMMPASVL TGNVIIE | 132.13 | $3.31 \times 10^{-22}$ | $3.00 \times 10^{7}$ | 0.915 |
| | E114 /S115 | AAPE(0.5)S(0.5)QMMPAS(0.006)V LTGNVIIE | 79.511 | $1.60 \times 10^{-8}$ | - | - |
| PDEδ·**13b** | | | | | | |
| R1 | E88/ E89 | QKVYFKGQCLE(0.5)E(0.5) | 75.78 | $1.70 \times 10^{-4}$ | $1.31 \times 10^{7}$ | N[f] |
| R2 | E88 | QKVYFKGQCLE(0.912)E(0.088) | 67.153 | $2.46 \times 10^{-4}$ | $2.16 \times 10^{7}$ | 0.276 |
| | E88/ E89 | QKVYFKGQCLE(0.45)E(0.55) | 74.76 | $1.84 \times 10^{-4}$ | $6.68 \times 10^{6}$ | 0.0851 |
| R3 | E88 | QKVYFKGQCLE(0.893)E(0.106) | 72.652 | $1.81 \times 10^{-4}$ | $1.40 \times 10^{7}$ | 0.229 |
| | E88/ E89 | QKVYFKGQCLE(0.54)E(0.46) | 75.045 | $1.80 \times 10^{-4}$ | $4.34 \times 10^{6}$ | 0.0708 |

a. Peptide sequences identified by searching against sequence of PDEδ (Uniprot ID, O43924) and a contaminant database with a false discovery rate of 1%, with localisation probability value (0 to 1) indicating the probability for a correct localisation of the modification from a MS/MS spectrum;
b. The Andromeda score of the best identified modified peptide containing this site;
c. The posterior error probability (PEP) of the best identified modified peptide containing this site;
d. Summed up intensities of the modified peptides;
e. Ratio of the intensities of the modified peptides to the unmodified peptides, not quantitative due to possibility of unequal ionisation efficiency;
f. No unmodified peptide of the specified sequence was identified in this sample.

**Supplementary Table 2.** Reactome Pathway Analysis of Global Proteome upon **22a** Treatment.

| **Table 2: Reactome Pathway Overrepresentation[6] of Significant Changes in Global Proteome.** | | | | | | |
|---|---|---|---|---|---|---|
| **Pathway name** | **#Entities found** | **Entities *p*-value** | **Entities FDR** | **#Reactions found** | **#Reactions total** | **Reactions ratio** |
| Retrograde transport at the Trans-Golgi-Network | 2 | 2.20E-03 | 1.79E-01 | 7 | 20 | 1.30E-03 |
| ARL13B-mediated ciliary trafficking of INPP5E | 1 | 3.78E-03 | 1.79E-01 | 4 | 4 | 2.61E-04 |
| Interleukin-10 signaling | 2 | 5.26E-03 | 1.79E-01 | 1 | 15 | 9.78E-04 |
| TNFR1-mediated ceramide production | 1 | 7.56E-03 | 1.88E-01 | 3 | 3 | 1.96E-04 |
| Interferon alpha/beta signaling | 2 | 1.15E-02 | 1.88E-01 | 2 | 25 | 1.63E-03 |
| Endosomal/Vacuolar pathway | 1 | 1.88E-02 | 1.88E-01 | 3 | 4 | 2.61E-04 |
| ATF6 (ATF6-alpha) activates chaperone genes | 1 | 1.88E-02 | 1.88E-01 | 3 | 5 | 3.26E-04 |
| Interferon gamma signaling | 2 | 2.08E-02 | 1.88E-01 | 2 | 23 | 1.50E-03 |
| ATF6 (ATF6-alpha) activates chaperones | 1 | 2.13E-02 | 1.88E-01 | 3 | 10 | 6.52E-04 |
| mRNA decay by 5' to 3' exoribonuclease | 1 | 2.62E-02 | 1.88E-01 | 3 | 3 | 1.96E-04 |
| Choline catabolism | 1 | 2.74E-02 | 1.88E-01 | 1 | 8 | 5.21E-04 |
| FOXO-mediated transcription of cell death genes | 1 | 2.87E-02 | 1.88E-01 | 3 | 15 | 9.78E-04 |
| Intra-Golgi and retrograde Golgi-to-ER traffic | 2 | 3.08E-02 | 1.88E-01 | 7 | 48 | 3.13E-03 |
| TNFR1-induced proapoptotic signaling | 1 | 3.24E-02 | 1.88E-01 | 1 | 3 | 1.96E-04 |
| TNFs bind their physiological receptors | 1 | 3.72E-02 | 1.88E-01 | 1 | 13 | 8.47E-04 |
| TGFBR3 expression | 1 | 3.85E-02 | 1.88E-01 | 2 | 8 | 5.21E-04 |
| Myogenesis | 1 | 3.97E-02 | 1.88E-01 | 2 | 14 | 9.13E-04 |
| TNFR1-induced NF-kappa-B signaling pathway | 1 | 4.09E-02 | 1.88E-01 | 5 | 5 | 3.26E-04 |
| ATF4 activates genes in response to endoplasmic reticulum stress | 1 | 4.21E-02 | 1.88E-01 | 1 | 7 | 4.56E-04 |
| Cytokine Signaling in Immune system | 4 | 4.55E-02 | 1.88E-01 | 6 | 791 | 5.16E-02 |
| Antigen Presentation: Folding, assembly and peptide loading of class I MHC | 1 | 5.06E-02 | 1.88E-01 | 13 | 16 | 1.04E-03 |
| PERK regulates gene expression | 1 | 5.18E-02 | 1.88E-01 | 1 | 11 | 7.17E-04 |
| RAS processing | 1 | 5.66E-02 | 1.88E-01 | 4 | 21 | 1.37E-03 |
| DAP12 interactions | 1 | 6.37E-02 | 1.88E-01 | 3 | 33 | 2.15E-03 |
| mRNA Splicing - Minor Pathway | 1 | 6.61E-02 | 1.88E-01 | 5 | 5 | 3.26E-04 |

Top 25 pathways ranked by entities *p*-values were shown.

**Supplementary Table 3.** Reactome Pathway Analysis of Upregulated Kinases upon **22a** Treatment.

**Table 3: Reactome Pathway Overrepresentation[6] of Significant Upregulated Kinases**

| Pathway name | #Entities found | Entities *p*-value | Entities FDR | #Reactions found | #Reactions total | Reactions ratio |
|---|---|---|---|---|---|---|
| Intracellular signaling by second messengers | 14 | 1.80E-12 | 1.20E-09 | 41 | 116 | 7.56E-03 |
| Signaling by Receptor Tyrosine Kinases | 16 | 2.02E-11 | 6.69E-09 | 75 | 759 | 4.95E-02 |
| Co-inhibition by CTLA4 | 6 | 1.58E-10 | 2.72E-08 | 3 | 5 | 3.26E-04 |
| VEGFA-VEGFR2 Pathway | 9 | 1.64E-10 | 2.72E-08 | 23 | 79 | 5.15E-03 |
| Signaling by VEGF | 9 | 3.34E-10 | 4.25E-08 | 23 | 86 | 5.61E-03 |
| Co-stimulation by CD28 | 7 | 3.86E-10 | 4.25E-08 | 15 | 19 | 1.24E-03 |
| Fc epsilon receptor (FCERI) signaling | 10 | 9.52E-10 | 9.04E-08 | 22 | 65 | 4.24E-03 |
| Signal Transduction | 28 | 1.33E-09 | 1.11E-07 | 341 | 2582 | 1.68E-01 |
| Regulation of KIT signaling | 5 | 2.81E-09 | 2.00E-07 | 4 | 8 | 5.21E-04 |
| Calmodulin induced events | 6 | 3.93E-09 | 2.00E-07 | 14 | 23 | 1.50E-03 |
| CaM pathway | 6 | 3.93E-09 | 2.00E-07 | 14 | 24 | 1.56E-03 |
| Fcgamma receptor (FCGR) dependent phagocytosis | 9 | 5.35E-09 | 2.52E-07 | 7 | 42 | 2.74E-03 |
| Ca-dependent events | 6 | 9.56E-09 | 3.92E-07 | 14 | 27 | 1.76E-03 |
| Interleukin-3, Interleukin-5 and GM-CSF signaling | 6 | 9.56E-09 | 3.92E-07 | 5 | 38 | 2.48E-03 |
| Signaling by SCF-KIT | 6 | 1.07E-08 | 4.15E-07 | 22 | 39 | 2.54E-03 |
| Regulation of T cell activation by CD28 family | 7 | 1.15E-08 | 4.15E-07 | 21 | 35 | 2.28E-03 |
| DAG and IP3 signaling | 6 | 1.35E-08 | 4.71E-07 | 15 | 28 | 1.83E-03 |
| Constitutive Signaling by AKT1 E17K in Cancer | 5 | 2.97E-08 | 9.81E-07 | 18 | 18 | 1.17E-03 |
| CD209 (DC-SIGN) signaling | 5 | 3.51E-08 | 1.09E-06 | 3 | 11 | 7.17E-04 |
| Diseases of signal transduction by growth factor receptors and second messengers | 12 | 3.93E-08 | 1.18E-06 | 67 | 516 | 3.36E-02 |
| Signaling by ERBB2 | 6 | 5.81E-08 | 1.63E-06 | 5 | 46 | 3.00E-03 |
| PLC beta mediated events | 6 | 6.33E-08 | 1.71E-06 | 14 | 32 | 2.09E-03 |
| C-type lectin receptors (CLRs) | 8 | 8.95E-08 | 2.33E-06 | 15 | 68 | 4.43E-03 |
| G-protein mediated events | 6 | 1.03E-07 | 2.54E-06 | 14 | 41 | 2.67E-03 |
| PECAM1 interactions | 4 | 1.06E-07 | 2.54E-06 | 1 | 7 | 4.56E-04 |

Top 25 pathways ranked by entities *p*-values were shown.

**Supplementary Table 4.** Reactome Pathway Analysis of Downregulated Kinases upon **22a** Treatment.

| **Table 4: Reactome Pathway Overrepresentation[6] of Significant Downregulated Kinases** | | | | | | |
|---|---|---|---|---|---|---|
| **Pathway name** | **#Entities found** | **Entities *p*-value** | **Entities FDR** | **#Reactions found** | **#Reactions total** | **Reactions ratio** |
| Signal Transduction | 7 | 5.72E-04 | 4.00E-03 | 45 | 2574 | 1.72E-01 |
| Disease | 4 | 6.66E-02 | 6.66E-02 | 37 | 1917 | 1.28E-01 |
| Oncogenic MAPK signaling | 3 | 2.86E-05 | 3.14E-04 | 33 | 46 | 3.07E-03 |
| Diseases of signal transduction by growth factor receptors and second messengers | 3 | 4.55E-03 | 1.91E-02 | 33 | 514 | 3.44E-02 |
| MAPK1/MAPK3 signaling | 3 | 1.04E-03 | 6.26E-03 | 32 | 82 | 5.48E-03 |
| MAPK family signaling cascades | 3 | 1.51E-03 | 9.05E-03 | 32 | 122 | 8.15E-03 |
| RAF/MAP kinase cascade | 3 | 9.72E-04 | 5.83E-03 | 25 | 75 | 5.01E-03 |
| MAP2K and MAPK activation | 3 | 3.38E-06 | 7.10E-05 | 12 | 12 | 8.02E-04 |
| Macroautophagy | 2 | 5.04E-03 | 1.91E-02 | 9 | 96 | 6.42E-03 |
| Autophagy | 2 | 6.10E-03 | 1.91E-02 | 9 | 117 | 7.82E-03 |
| RAF activation | 3 | 2.10E-06 | 7.10E-05 | 8 | 12 | 8.02E-04 |
| RAF-independent MAPK1/3 activation | 2 | 1.45E-04 | 1.16E-03 | 7 | 12 | 8.02E-04 |
| Signaling by RAF1 mutants | 3 | 3.63E-06 | 7.10E-05 | 6 | 7 | 4.68E-04 |
| Generic Transcription Pathway | 2 | 2.44E-01 | 2.44E-01 | 6 | 878 | 5.87E-02 |
| RNA Polymerase II Transcription | 2 | 2.80E-01 | 2.80E-01 | 6 | 939 | 6.28E-02 |
| Gene expression (Transcription) | 2 | 3.48E-01 | 3.48E-01 | 6 | 1138 | 7.61E-02 |
| Negative regulation of MAPK pathway | 3 | 4.17E-06 | 7.10E-05 | 5 | 17 | 1.14E-03 |
| Paradoxical activation of RAF signaling by kinase inactive BRAF | 3 | 5.07E-06 | 7.10E-05 | 5 | 7 | 4.68E-04 |
| Signaling by moderate kinase activity BRAF mutants | 3 | 5.07E-06 | 7.10E-05 | 5 | 7 | 4.68E-04 |
| Signaling downstream of RAS mutants | 3 | 5.07E-06 | 7.10E-05 | 5 | 7 | 4.68E-04 |
| Signaling by RAS mutants | 3 | 5.07E-06 | 7.10E-05 | 5 | 9 | 6.02E-04 |
| Signaling by BRAF and RAF1 fusions | 3 | 1.39E-05 | 1.67E-04 | 5 | 5 | 3.34E-04 |
| Energy dependent regulation of mTOR by LKB1-AMPK | 1 | 2.17E-02 | 4.34E-02 | 5 | 7 | 4.68E-04 |
| Transcriptional Regulation by TP53 | 2 | 2.87E-02 | 4.66E-02 | 5 | 257 | 1.72E-02 |
| MTOR signaling | 1 | 3.06E-02 | 4.66E-02 | 5 | 29 | 1.94E-03 |

Top 25 pathways ranked by entities *p*-values were shown.

**Supplementary Table 5.** X-ray Crystallography Data Collection and Refinement Statistics.

Statistics for the highest resolution shell are shown in parentheses.

| **PDB ID** | **9RP6** | **9RP7** |
|---|---|---|
| Crystal and ligand | PDEδ•**13b** | PDEδ•**22a** |
| | | |
| **Data collection** | | |
| Space group | P 32 2 1 | P 32 2 1 |
| Unit cell dimensions | | |
| *a*, *b*, *c* (Å) | *a* = 55.65, *b* = 55.65, *c* = 115.84 | *a* = 55.52, *b* = 55.52, *c* = 114.96 |
| α, β, γ (°) | α =90, β =90, γ = 120 | α =90, β =90, γ = 120 |
| Wavelength | 1.00 | 1.00 |
| Resolution range (Å) | 48.19 - 1.8 (1.89 - 1.8) | 38.32 - 1.9 (1.968 - 1.9) |
| Total reflections | 389557 (52957) | 333883 (31736) |
| Unique reflections | 37182 (5297) | 16807 (1651) |
| *R*-meas | 0.09882 (1.965) | 0.1339 (2.457) |
| *R*-pim | 0.0304 (0.6172) | 0.0299 (0.5574) |
| *R*-merge | 0.09397 (1.864) | 0.1304 (2.392) |
| $CC_{1/2}$ | 0.999 (0.634) | 0.999 (0.795) |
| Mean I/σ (I) | 11.94 (0.80) | 13.74 (1.02) |
| Completeness (%) | 99.74 (99.46) | 99.69 (99.21) |
| | | |
| **Refinement** | | |
| Resolution (Å) | 1.8 | 1.9 |
| Reflections used in refinement | 19875 (2760) | 16762 (1641) |
| Reflections used for *R*-free | 996 (138) | 837 (82) |
| *R*-work | 0.2052 (0.3318) | 0.2071 (0.3294) |
| *R*-free | 0.2279 (0.3794) | 0.2231 (0.3745) |
| Protein residues | 147 | 150 |
| Number of non-H atoms | 1346 | 1376 |
| macromolecules | 1218 | 1235 |
| ligands | 50 | 87 |
| solvent | 78 | 97 |
| Wilson B-factor | 35.20 | 39.38 |
| Average B-factor | 42.40 | 49.26 |
| macromolecules | 42.05 | 49.01 |
| ligands | 45.56 | 43.23 |
| solvent | 45.73 | 55.23 |
| Root mean square deviation | | |
| RMS (bonds) (Å) | 0.004 | 0.009 |
| RMS (angles) (°) | 0.62 | 1.15 |
| Ramachandran favoured (%) | 98.60 | 97.97 |
| Ramachandran allowed (%) | 1.40 | 1.35 |
| Ramachandran outliers (%) | 0.00 | 0.68 |
| Rotamer outliers (%) | 0.74 | 0.74 |
| Clashscore | 2.76 | 2.79 |

## 2. Methods

Mass Spectrometry Analysis of Covalent Peptide Adducts of PDEδ after Glu-C Digestion

Covalent adducts of PDEδ or unbound vehicle DMSO treated PDEδ (1.5 µg/sample) were denatured, reduced, alkylated and digested by Glu-C and desalted before analysis by mass spectrometry. For denaturation, to 4 µl of each sample was added 18 µl of 8 M guanidine hydrochloride (cas 50-01-1, Carl Roth, #0037.1) solution (to a final 6.5 M) and boiled for 15 min at 95 °C. For reduction, the solution was then cooled and added 0.5 µl of 50 mM dithiothreitol (DTT, cas 3483-12-3, Gerbu Biotechnik, #1008-100g) solution (to a final 1 mM DTT) and incubated for 20 min at 60 °C. Subsequently, for alkylation, 2.5 µl of 50 mM 2-chloroacetamide (CAA, cas 79-07-2, Sigma Aldrich, #22790) was added to each sample (to a final 5 mM CAA) and incubated for 30 min at room temperature with protection from light. For each sample, 225 µl of 20 mM ammonium bicarbonate (cas 1066-33-7, Sigma Aldrich, #A6141-500g) solution was then added to dilute the concentration of guanidine hydrochloride to less than 0.8 M. For digestion, 1.5 µl of Glu-C (0.05 µg/µl, Promega V1651) was added to each sample (1:20 enzyme to protein, w/w) and incubated overnight at 37 °C, 400 rpm with protection from light. On the next day, the reaction was quenched by adding 5 µl of 10% trifluoracetic acid (TFA, cas 76-05-1, Sigma Aldrich, #302031) and samples were desalted by stage tip purification with C18 extraction disks (Empore™ high performance extraction disks, 47 mm, 3M Bioanalytical Technologies #2215). Each stage tip (2 layers of C18 disks) was activated by 100 µl methanol, washed once by 100 µl of buffer B (containing 0.1% formic acid, 80% acetonitrile in water) followed by twice washing with 100 µl of buffer A (containing 0.1% formic acid in water) prior to sample loading. Loaded stage tip was further washed once with 100 µl of buffer A and sample was eluted with 20 µl of buffer B, with centrifugation at 4000 rpm at room temperature for 5 min and then dried by SpeedVac at 30 °C.

For nanoHPLC-MS/MS analysis, samples were redissolved in 15-20 µl of 0.1% TFA and 3 µl each were loaded onto a pre-column cartridge for desalting. Desalting was performed for 5 min using 0.1% TFA as eluent with a flow to waste (30 µl/min) followed by back-flushing of the sample during the whole analysis from the pre-column to the PepMap100 RSLC C18 nano-HPLC column (2 µm, 100 Å, 75 µm ID × 50 cm, nanoViper, Dionex, Germany) using a linear gradient starting with 95% solvent A ($H_2O$ with 0.1% formic acid)/5% solvent B (acetonitrile with 0.1% formic acid) and increasing to 30% solvent B after 35 min (measurements on Q-Exactive Plus) or increasing stepwise to 20% solvent B after 37 min and further to 32% solvent B after 44 min (measurements on Q-Exactive HF) using a flow rate of 300 nl/min. The nano-HPLC was online coupled to a Quadrupole-Orbitrap Q-Exactive Plus or a Q-Exactive HF Mass Spectrometer using an uncoated SilicaTip (ID 20 µm, Tip-ID 10 µM). Mass range of m/z 300 to 1650 (Q-Exactive Plus) or 375 to 1500 (Q-Exactive HF) was acquired with a resolution of 70000 (Q-Exactive Plus) or 120000 (Q-Exactive HF) for full scan, followed by up to ten (Q-Exactive

Plus) or fifteen (Q-Exactive HF) high energy collision dissociation (HCD) MS/MS scans of the most intense at least doubly charged ions using a resolution of 17500 (Q-Exactive Plus) or 15000 (Q-Exactive HF) and a NCE energy of 25% (Q-Exactive Plus) or 27% (QExactive HF).

Data evaluation was performed using MaxQuant software (v.2.2.0.0).[13] The spectra were queried against the PDE6D sequence (Uniprot ID: O43924) and a contamination database with a 1 % false discovery rate, utilising a decoy database to analyse the false discovery rate. For database search, oxidation of methionine and *N*-terminal acetylation, carbamidomethylation of cysteines, and artificial modification of amino acids cysteines (C), aspartic acids (D), glutamic acids (E), threonine (T), serine (S), tyrosines (Y), lysine K, or histidines (H) have been set as variable modifications. Cleavages by Glu-C were analysed based on the enzyme that was used for digestion. All experiments were performed in three biological triplicates.

Global Proteome and Phosphoproteome Analysis

PA-TU-8902 cells (8 × $10^6$ cells/dish) were seeded in two 15 cm dishes and incubated in a humidified atmosphere at 37 °C and 5% $CO_2$ overnight. Cells were then treated with 5 µM of compound **22a** or DMSO, with 0.1% DMSO adjusted for both conditions in fresh medium for 2 h incubated in a humidified atmosphere at 37 °C and 5% $CO_2$. Cells were washed twice with warm PBS (phosphate buffered saline) and once with warm TBS (Tris buffered saline containing 50 mM Tris-HCl, pH = 7.5, 138 mM NaCl, 2.7 mM KCl) and lysed by incubating with boiling guanidine hydrochloride lysis buffer (1 ml/dish, freshly prepared, containing 6 M guanidine HCl, 100 mM Tris HCI, pH = 8.5, 10 mM TCEP, 40 mM CAA) at 96 °C for 5 min in a thermomixer with protection from light. The collected lysates were sonicated by four cycles of 30s sonication and 30s rest (Bandelin SONOPULS HD2070, 60% power, 70% duty cycle) and centrifuged at 16,000 g for 30 min at room temperature. The supernatants were transferred into new tubes and snap frozen by liquid nitrogen and kept at -80 °C until further usage. Upon thawing, samples were homogenised again with four cycles of sonication and protein concentration was determined by Pierce™ 660 nm protein assay according to the manufacturer's instruction (Thermo Scientific, #22662). For each sample, 1 mg of protein was digested on Sera-Mag beads (GE Healthcare, #45152105050250 and #65152105050250) with trypsin/Lys-C mix (Promega, V5072). A 20 µg/ul suspension of 1:1 mix of hydrophilic and hydrophobic Sera-Mag™ SpeedBead™ carboxylate modified magnetic particles was first prepared and added to prediluted protein samples with 1 M of guanidine HCl. Acetonitrile was added to a final 70% v/v to precipitate proteins on beads with a total of 20 min incubation and occasional mixing. Beads with aggregates of proteins were collected with a magnetic rack, washed once with acetonitrile followed by an additional wash with 70% ethanol and dried for 5 min at room temperature. To each sample, 260 µl of digestion buffer (100 mM

triethylammonium bicarbonate, cas 15715-58-9, Sigma Aldrich, T7408-100ml; 10% 2,2,2-trifluoroethanol TFE, cas 75-89-8, Fisher Scientific, #10051560) was added followed by 40 µl of trypsin/Lys-C mix (1 µg/µl in resuspension buffer containing 50 mM acetic acid, 1:25 w/w enzyme to protein ratio) and incubated at 37 °C in a thermomixer with 1000 rpm overnight. The reaction was quenched by adding 15 µl of 10% TFA to a final 0.5% v/v and the supernatant was collected in a fresh tube. Approximately 10 µg/sample was desalted by stage tip purification with C18 extraction disks (Empore$^{TM}$ high performance extraction disks, 47 mm, 3M Bioanalytical Technologies #2215) and subsequently used for LC-MSMS analysis for global proteome profiling. The rest of the protein digest (approximately 990 µg/sample) was enriched for phosphopeptides with MagReSyn $TiO_2$ particles (ReSyn Biosciences, MR-TID005) with procedure adapted from the manufacturer's instruction and phosphoproteomics with the EasyPhos platform.[14] The samples were first dried in SpeedVac at 30 °C and resuspended in loading buffer (400 µl/sample, containing 1 M glycolic acid (cas 79-14-1, VWR, #1041060100), 80% acetonitrile, 5% TFA). Supernatants were collected by centrifugation at 10,000 g for 5 min at 4 °C and added to prepared $TiO_2$ particles in loading buffer (200 µl/sample, 2:1 w/w beads to peptides, prewashed with 200 µl of 70% EtOH and twice with 200 µl loading buffer), incubated for 20 min at room temperature with continuous mixing on a rolling shaker. The particles were then washed once with 200 µl loading buffer, twice with 200 µl wash buffer 1 (containing 1% TFA in 80% acetonitrile/$H_2O$) and twice with 200 µl wash buffer 2 (containing 0.2% TFA in 10% acetonitrile/$H_2O$), each time with 550 rpm gentle agitation for 2 min on a thermomixer before removal of wash buffers. Bound phosphopeptides were eluted from the microparticles by adding three times of 80 µl elution buffer (freshly prepared, 1% $NH_4OH$ in water) and incubation for 10 min with constant gentle agitation at 550 rpm. The 80 µl elution buffer was transferred into a new tube containing 20 µl 15% TFA and three rounds of elution were pooled together for a total of 300 µl/sample. The samples were centrifuged at 10,000 g at room temperature for 10 min to remove particulate material and eluates were frozen by liquid nitrogen and kept at -80 °C for 30 min before vacuum drying (to approximately 15 µl/sample) in a SpeedVac at 30 °C. Enriched phosphopeptides were reconstituted in SDB-RPS loading buffer (1% TFA in isopropanol, to 200 µl/sample) and desalted by stage tip purification (2 stage tips/sample, 2 layers of SBD-RPS and 1 layer of C8 on top/stage tip) with SDB-RPS extraction disks (Sigma Aldrich, 66886-U) and C8 extraction disks (Sigma Aldrich, 66882-U). Each stage tip was equilibrated with 50 µl acetonitrile, 50 µl equilibration buffer (containing 30% methanol, 0.2% TFA in water) and 50 µl of wash buffer 1 (containing 0.2% TFA in water) prior to loading. Each loaded stage tip was washed with 100 µl SDB-RPS loading buffer, 100 µl of wash buffer 1 and 100 µl of wash buffer 2 (containing 0.2% TFA, 5% acetonitrile in water), each time by centrifuging the samples to near dryness with 500 g for 5 min at room temperature. Phophopeptides were eluted with freshly prepared elution buffer (containing 0.1% $NH_4OH$ in 60% acetonitrile in water, 60 µl/stage tip, a total of 120 µl/sample) and immediately

dried in a SpeedVac at 45 °C. After approximately 45 min, the samples were acidified by adding 10 µl of 0.3% TFA, 2% acetonitrile in water to each sample, mixed at 1400 rpm for 2 min, centrifuged at 2000 g for 1 min at room temperature and further dried in a SpeedVac at 45 °C.

For nanoHPLC-MS/MS analysis of non-enriched samples, samples were dissolved in 15 µl of 0.1% TFA in water and 4.5 µl were loaded onto a pre-column cartridge for desalting. Desalting was performed for 5 min using 0.1% TFA as eluent with flow to waste (30 µl/min) followed by back-flushing of the sample during the whole analysis from the pre-column to the PepMap100 RSLC C18 nano-HPLC column (2 µm, 100 Å, 75 µm ID × 50 cm, nanoViper, Dionex, Germany) using a linear gradient starting with 95% solvent A ($H_2O$ with 0.1% formic acid)/5% solvent B (acetonitrile with 0.1% formic acid) and increasing to 20% solvent B after 115 min and further increasing to 32% solvent B after 135 min using a flow rate of 300 nl/min. The nano-HPLC was online coupled to a Q-Exactive HF Orbitrap Mass Spectrometer using an uncoated SilicaTip (ID 20 µm, Tip-ID 10 µM). Mass range of m/z 375 to 1500 was acquired with a resolution of 120,000 for full scan, followed by up to fifteen high energy collision dissociation (HCD) MS/MS scans of the most intense at least doubly charged ions using a resolution of 15,000 and a NCE energy of 27%.

For nanoHPLC-MS/MS analysis of phospho-enriched samples, samples were dissolved in 10 µl of 0.1% TFA in water and 5 µl were loaded onto a pre-column cartridge for desalting. Desalting was performed for 5 min using 0.1% TFA as eluent with flow to waste (30 µl/min) followed by back-flushing of the sample during the whole analysis from the pre-column to the PepMap100 RSLC C18 nano-HPLC column (2 µm, 100 Å, 75 µm ID × 50 cm, nanoViper, Dionex, Germany) using a linear gradient starting with 95% solvent A ($H_2O$ with 0.1% formic acid)/5% solvent B (acetonitrile with 0.1% formic acid) and increasing to 20% solvent B after 115 min and further increasing to 32% solvent B after 135 min using a flow rate of 300 nl/min. The nano-HPLC was online coupled to a Q-Exactive HF Orbitrap Mass Spectrometer using an uncoated SilicaTip (ID 20 µm, Tip-ID 10 µM). Mass range of m/z 375 to 1500 was acquired with a resolution of 120,000 for full scan, followed by up to fifteen high energy collision dissociation (HCD) MS/MS scans of the most intense at least doubly charged ions using a resolution of 30,000 and a NCE energy of 27%.

Data evaluation was performed using MaxQuant software (v.2.4.14.0).[13] The spectra were queried against the human taxonomy of Uniprot and a contamination database with a 1% false discovery rate, utilizing a decoy database to analyze the false discovery rate. For database search, carbamidomethylation on cysteine residues was designated as a static modification. Additionally, phosphorylations on serine, threonine, and tyrosine, as well as acetylation on *N*-termini, and oxidation

of methionine were set as variable modifications. Trypsin-specific cleavages allowing up to 2 missed cleavages were included in the analysis. Raw-data of non-enriched and phospho-enriched samples were searched together. For protein quantification just data of non-enriched samples were taken into account. All experiments were performed in biological triplicates. The output files from MaxQuant were further analyzed by PhosphoAnalyst,[15] followed by kinase-substrate enrichment analysis using KSEA App with NetworKIN[7-9] and Reactome pathway overrepresentation.[6] For phospho-enriched samples a site probability threshold above 75 was employed for phosphosites and data were normalized to the total peptide content.

*$Kras^{LSL.G12D/wt}$; $Trp^{53fl/fl}$ (KP)* Mouse Model Study

Lung Cancer Induction

We employed the *$Kras^{LSL.G12D/wt}$;$Trp^{53fl/fl}$ (KP)* mouse line as described previously.[16] For lung tumor induction, 8–15-week-old mice were anesthetized via intraperitoneal injection of ketamine (100 mg/kg) and xylazine (10 mg/kg). Upon reaching adequate surgical tolerance, mice were administered $2.5\times10^7$ PFU of replication-deficient adenovirus encoding Cre-recombinase (Adeno-Cre) intratracheally, and carefully monitored during recovery. Mice were scored for general and disease specific burden and dissection upon reaching heavy burden (score 20 on score sheet).

*In vivo* Tumor Progression Monitoring with µCT

Instilled mice underwent µCT scans to track tumor development using a Skyscan 1176 high-resolution µCT imaging system (Bruker). Mice were anesthetized with 3 Vol. % isoflurane through an isoflurane vaporizer (Dräger). Thoracic µCT images were acquired at 475 µA and 50 kV with a 35 µm resolution using a 0.5 mm aluminum filter. The images were reconstructed using NRecon software (Bruker) and analyzed in DataViewer software for presence of tumors (Bruker).

*In vivo* Lung Cancer Treatment

Mice were included into the survival monitoring upon identification of a tumor in µCT. Tumor bearing mice were randomly assigned to untreated and treated groups. Compound solution for treatment was prepared with 1.5 mg/ml Deltafluorine (**22a**) in 30% 2-Hydroxypropyl-*β*-cyclodextrin and 0.5 % (v/v) Hydroxypropyl-methylcellulose in ddH$_2$O. Mice were treated with intraperitoneal (i.p.) injections at 15 mg/kg/day with a maximum volume of 0.1 mL/10g body weight for 21 days. The GV-SOLAS recommendations for substance administration in experimental animals were followed. In pharmacodynamic preliminary studies approved by the veterinary office of Saarland (file number C1_2.4.2.2 14-2020) conducted on non-tumor-bearing mice, the intraperitoneal administration of 15

mg/kg/day for 21 days was established as the optimal dosage and method of administration. Mice were scored for general and disease specific burden. The treated animals exhibited drowsiness for about 4 h after the first doses and isolated themselves. Additionally, a ruffled coat was observed after the first few days. All observed signs normalized after a few days.

SPD Lung Tumor Volume Measurements

µCT images of mouse lungs were quantified using HOROS (HOROS project) software. Tumors were measured by width and length in the transverse plane. The sum of the product of means (SPD) was used as a metric of tumor size and, when cumulated, tumor load.

3D CT Image Processing

For 3D visualization of mouse lungs and tumors, individual tumors were identified and marked as ROIs within each animal's µCT transverse image stack. Tumors were segmented using threshold values and interpolated in Bruker CTAn software, with 3D visualizations created in Bruker's CTVox software. The analyst was blinded to genotypes and treatments during tumor ROI identification.

Animal Housing

In the animal husbandry of the MPI for Biology of Ageing, gamma-irradiated (25 kGy) laboratory animal feed was used and drinking water was sterile-filtered. The light cycle in the enclosure was 12 h light/12 h dark phase, the humidity was 50 % (± 5 %) and the room temperature was 22 °C (± 1 °C). The air exchange rate in the room was 8-fold. The animals were kept in individually ventilated cages (IVC).

Licensing

The mouse experiments were licensed by the State Agency for Nature, Environment and Consumer Protection (LANUV) under the license 81-02.04.2020.A281. Breeding of mouse lines was permitted by the State Agency for Nature, Environment and Consumer Protection (LANUV) under the license VSG 81-02.04.2019.A009 and 2024-225.

## 3. Chemical Synthesis

General Information

Unless otherwise noted, all commercially available reagents were purchased from Sigma Aldrich or TCI Chemicals and were used as provided without further purifications. Solvents for chromatography were technical grade, purchased from VWR Chemicals. Analytical thin-layer chromatography (TLC) was performed on Merck silica gel aluminium plates with F-254 indicator. Compounds were visualised by irradiation with UV light, potassium permanganate staining with heating by a heat gun. Column chromatography was performed using silica gel Merck 60 (particle size 0.040-0.063 mm).

$^{1}$H-NMR and $^{13}$C-NMR were recorded on a *Bruker DRX400* (400 MHz), *Bruker DRX500* (500 MHz), *INOVA500* (500 MHz) and *Bruker DRX700* (700 MHz) at room temperature using $CDCl_3$ or DMSO-*d6* as solvent. Data are reported in the following order: chemical shift (δ) values are reported in ppm with the solvent resonance as internal standard ($CDCl_3$: δ = 7.26 ppm for $^{1}$H, δ = 77.16 ppm for $^{13}$C; DMSO-*d6*: δ = 2.50 ppm for $^{1}$H, δ = 39.52 ppm for $^{13}$C); multiplicities are indicated br s (broadened singlet), s (singlet), d (doublet), t (triplet), q (quartet), m (multiplet); coupling constants (*J*) are given in Hertz (Hz).

HPLC-MS analyses were performed with an Agilent 1100 Series connected to a Thermo LCQ Advantage mass spectrometer or Velos Pro HPLC using a C18 HPLC column 3 µm from Macherey Nagel. Eluent A: water with 0.1% TFA; Eluent B: acetonitrile with 0.1% TFA. Method: 10-100% B gradient on C18 column, 6 min, ambient temperature; or long analysis method 5-65 % B gradient on C18, 45 min, ambient temperature. High resolution mass spectra were recorded on a *LTQ Orbitrap* mass spectrometer coupled to an *Accela HPLC*-System (HPLC column: *Hypersyl GOLD*, 50 mm x 1 mm, particle size 1.9 µm, ionization method: electron spray ionization). All compounds investigated were isolated and purified and are ≥ 95% pure by HPLC analysis.

General scheme for compound synthesis

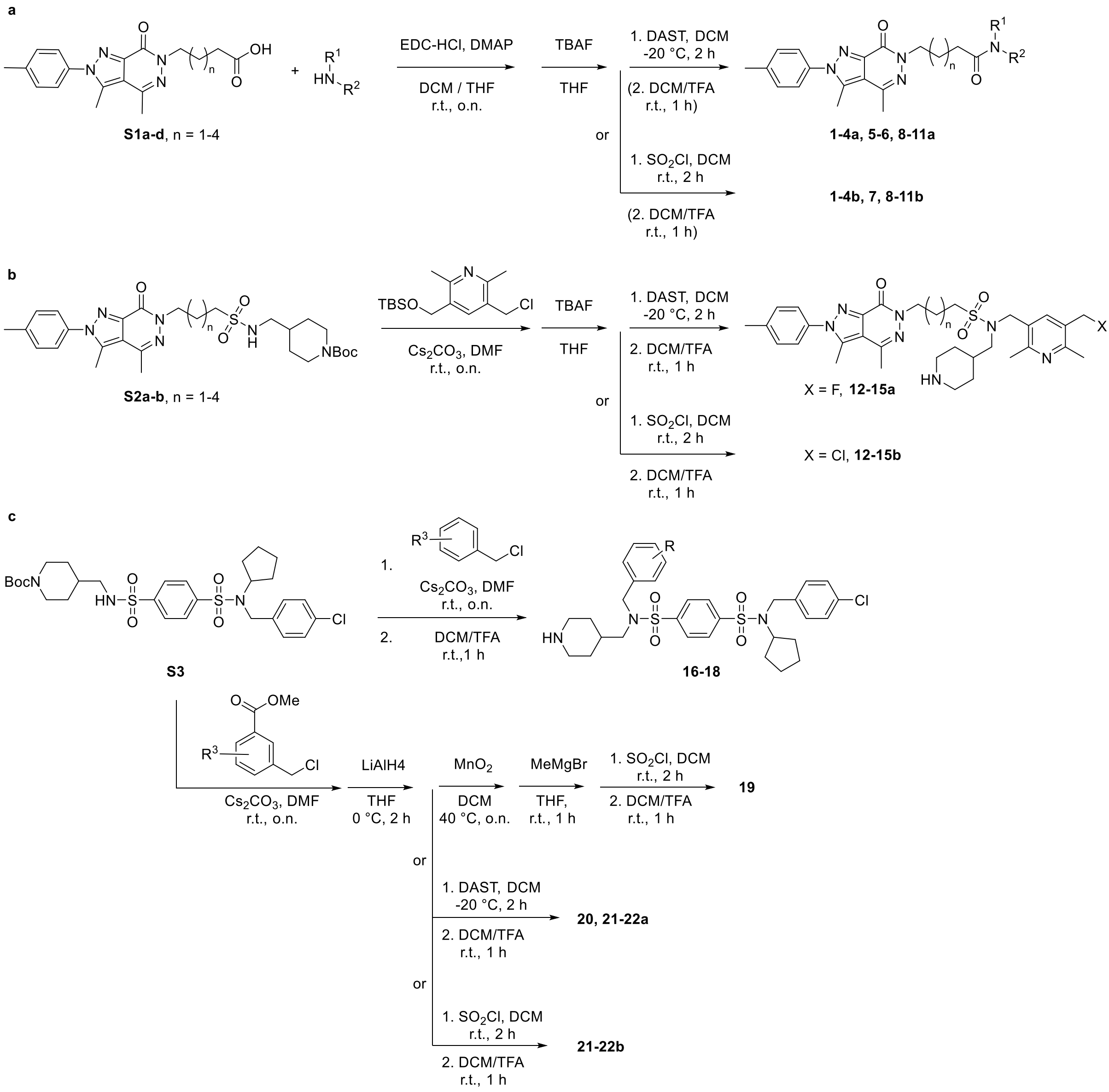


## General procedure for amide coupling (A)

The advanced intermediates **S1a-d** were synthesized according to previously published methods.[2, 5] The following steps starting from **S1** and the respective amine were carried out as one multi-step process and the intermediates were used without further characterization. To a solution of **S1** (1 eq) and DMAP (1.3 eq) in THF/DCM (1:1, 0.12 M) was added EDC-HCl (1.3 eq), followed by the corresponding amine derivative (1.1 eq) and the resulting solution was stirred overnight at room temperature. After completion, the reaction mixture was diluted with DCM and quenched by adding saturated $NaHCO_3$ solution. The aqueous layer was extracted with EtOAc and combined organic layers were sequentially

washed with saturated $NH_4Cl$ solution, $H_2O$ and brine, dried over $MgSO_4$, filtered and concentrated under vacuum.

General procedure for sulfonamide alkylation (B)

The advanced intermediates **S2** and **S3** were synthesized according to previously published methods.[2, 3, 5] The following steps were carried out as one multi-step process from **S2** or **S3** and the intermediates were used without further characterization. A solution of the respective secondary sulfonamide (**S2** or **S3)** in DMF was treated with $Cs_2CO_3$ (4 eq) and stirred at r.t. for 45 min. The corresponding alkyl halide (1.05 eq) was then added, and the mixture was stirred at r.t. until completion. Water was added to quench the reaction and the product was extracted into EtOAc. The combined extracts were dried over $MgSO_4$ and filtered. The crude product was purified by flash column chromatography on silica gel with ethyl acetate/cyclohexane as an eluent.

General procedure for deprotection and halogenation (C)

To amide or sulfonamide resulting from general procedure A or B with TBS protected alcohol (general scheme, **a** and **b**), TBS deprotection was carried out by adding TBAF (1.5 eq) dropwise in dry THF (0.06 M). The reaction was stirred at room temperature for 1 h until full deprotection and followed by the addition of $H_2O$. The mixture was extracted with EtOAc, washed with brine, dried over $MgSO_4$, filtered, concentrated *in vacuo* and used without further purification.

To amide or sulfonamide resulting from general procedure A or B with ester (general scheme, **c**), $LiAlH_4$ (1.0 M in THF, 0.75 eq) was added dropwise at 0 °C in dry THF (0.5 mL/mmol). The solution was stirred for 2 h and then quenched by adding $Na_2SO_4{\cdot}xH_2O$ portion-wise. The mixture was warmed to room temperature and THF was removed *in vacuo.* The product was extracted with ethyl acetate. The organic layers were separated, then washed with brine, dried over $MgSO_4$, filtered and concentrated *in vacuo*. The product was used in the next reaction without further purification.

For secondary alcohol synthesis, activated $MnO_2$ (20 eq) was added to a solution of the primary alcohol (1 eq) in DCM. The resulting suspension solution was stirred overnight at 40 °C and then filtered through Celite. The filter residue was washed with DCM, and the combined filtrate and washings were concentrated to give the aldehyde. To the resulting aldehyde in THF, MeMgBr (1 M solution in THF, 1 eq) was added at 0 °C. The solution was stirred at room temperature for 1 h and then quenched with a solution of sat. aq. $NH_4Cl$, which was extracted with ethyl acetate and the organic layers were separated, washed with brine, dried over $MgSO_4$, filtered and concentrated *in vacuo*. The product was used in the next reaction without further purification.

To the alcohol (1.0 eq) in dry DCM (0.08 M), DAST (3.0 eq) was added dropwise at -20 ºC. The solution was stirred in ice bath for 2 h and then quenched with aq. $NaHCO_3$. The product was extracted with DCM and the organic layers were separated, washed with brine, dried over $Na_2SO_4$, filtered and concentrated *in vacuo*. Then the mixture was added by TFA/DCM (1:1, 0.1 M, if Boc deprotection needed) stirred at room temperature for another 2 h. The reaction was concentrated and purified by column chromatography to give the desired fluoro-substituted products.

Alternatively, to the resulting alcohol (1.0 eq) in dry DCM (0.08 M) at 0 °C was added $SOCl_2$ (2.0 eq) dropwise. The reaction was warmed to room temperature and stirred for 2 h. Then the mixture was added by TFA/DCM (1:1, 0.1 M). After completion, the reaction was concentrated and purified by column chromatography to give the desired chloro-substituted products.

<u>Compound Characterization</u>

**4-(3,4-Dimethyl-7-oxo-2-(*p*-tolyl)-2,7-dihydro-6*H*-pyrazolo[3,4-d]pyridazin-6-yl)-*N*-(3-(fluoromethyl)benzyl)butanamide (1a)**

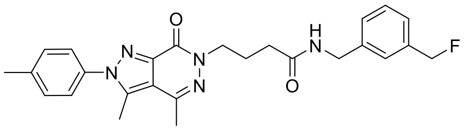

**$^{1}$H NMR (500 MHz, Chloroform-*d*)** δ 7.57 (s, 1H, amide NH), 7.40 – 7.29 (m, 8H), 5.35 (d, *J* = 47.8 Hz, 2H), 4.49 (d, *J* = 6.0 Hz, 2H), 4.24 (t, *J* = 5.9 Hz, 2H), 2.63 (s, 3H), 2.53 (s, 3H), 2.45 (s, 3H), 2.30 – 2.25 (m, 2H), 2.23 – 2.15 (m, 2H). **$^{13}$C NMR (126 MHz, Chloroform-*d*)** δ 173.0, 157.2, 142.0, 141.9, 140.1, 139.4, 136.6, 136.0, 130.0, 129.0, 128.4, 127.1 (2C), 126.4, 125.9 (2C), 117.9, 85.3, 84.0, 48.8, 43.5, 33.5, 25.9, 21.4, 20.0, 12.5. **$^{19}$F NMR (470 MHz, Chloroform-*d*)** δ -207.05 (t, *J* = 47.8 Hz).

***N*-(3-(chloromethyl)benzyl)-4-(3,4-dimethyl-7-oxo-2-(*p*-tolyl)-2,7-dihydro-6*H*-pyrazolo[3,4-d]pyridazin-6-yl)butanamide (1b)**

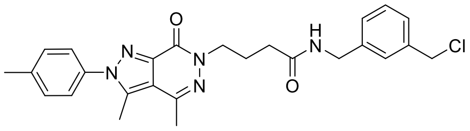

**$^{1}$H NMR (500 MHz, Chloroform-*d*)** δ 7.59 (s, 1H, amide NH), 7.38 – 7.25 (m, 8H), 4.55 (s, 2H), 4.47 (d, *J* = 5.9 Hz, 2H), 4.23 (t, *J* = 5.9 Hz, 2H), 2.62 (s, 3H), 2.53 (s, 3H), 2.44 (s, 3H), 2.31 – 2.23 (m, 2H), 2.23 – 2.14 (m, 2H). **$^{13}$C NMR (126 MHz, Chloroform-*d*)** δ 173.0, 157.2, 142.0, 141.9, 140.0, 139.6, 137.8, 136.6, 136.0, 130.0, 129.1, 128.1, 128.0, 127.5 (2C), 125.9 (2C), 117.9, 48.7, 46.3, 43.4, 33.5, 25.9, 21.4, 20.0, 12.4.

**4-(3,4-Dimethyl-7-oxo-2-(*p*-tolyl)-2,7-dihydro-6*H*-pyrazolo[3,4-d]pyridazin-6-yl)-*N*-(3-(fluoromethyl)benzyl)-*N*-methylbutanamide (2a)**

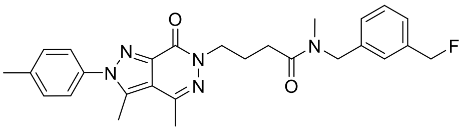

**$^1$H NMR (500 MHz, Chloroform-*d*)** (as a 1 : 0.6 mixture of rotamers) δ 7.38 – 7.11 (m, 16H), 5.36 (d, *J* = 47.7 Hz, 4H), 4.60 (s, 2H, major rotamer), 4.53 (s, 2H, minor rotamer), 4.29 (t, *J* = 6.7 Hz, 2H, major rotamer), 4.23 (t, *J* = 6.7 Hz, 2H, minor rotamer), 2.93 (s, 3H, minor rotamer), 2.90 (s, 3H, major rotamer), 2.62 (s, 3H, major rotamer), 2.61 (s, 3H, minor rotamer), 2.55 (s, 3H, major rotamer), 2.51 (s, 3H, minor rotamer), 2.51 – 2.46 (m, 4H), 2.45 (s, 6H), 2.28 – 2.18 (m, 4H). **$^{13}$C NMR (126 MHz, Chloroform-*d*)** (for a pair of rotamers) δ 173.0, 172.7, 156.5 (2C), 142.2 (2C), 141.0 (2C), 139.9 (2C), 138.2 (2C), 136.6 (2C), 136.2, 136.1, 130.0 (2C), 129.0 (2C), 128.5 (2C), 128.5 (2C), 127.2 (2C), 127.1 (2C), 126.9, 126.6, 126.6, 125.9, 125.4 (2C), 117.8 (2C), 85.3, 84.0, 53.3 (2C), 50.8, 49.1, 35.1, 34.1, 30.7, 30.4, 24.8, 24.6, 21.4 (2C), 20.0 (2C), 12.4 (2C). **$^{19}$F NMR (470 MHz, Chloroform-*d*)** δ -206.97 (t, *J* = 47.8 Hz), -207.75 (t, *J* = 47.7 Hz).

***N*-(3-(chloromethyl)benzyl)-4-(3,4-dimethyl-7-oxo-2-(*p*-tolyl)-2,7-dihydro-6*H*-pyrazolo[3,4-d]pyridazin-6-yl)-*N*-methylbutanamide (2b)**

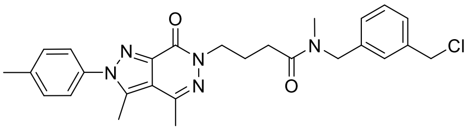

**$^1$H NMR (500 MHz, Chloroform-*d*)** (as a 1 : 0.5 mixture of rotamers) δ 7.38 – 7.27 (m, 12H), 7.26 – 7.07 (m, 4H), 4.58 (s, 2H, major rotamer), 4.56 (s, 2H, major rotamer), 4.56 (s, 2H, minor rotamer), 4.52 (s, 2H, minor rotamer), 4.29 (t, *J* = 6.7 Hz, 2H, major rotamer), 4.23 (t, *J* = 6.8 Hz, 2H, minor rotamer), 2.92 (s, 3H, minor rotamer), 2.90 (s, 3H, major rotamer), 2.62 (s, 3H, major rotamer), 2.60 (s, 3H, major rotamer), 2.54 (s, 3H, major rotamer), 2.51 (s, 3H, minor rotamer), 2.51 – 2.45 (m, 4H), 2.44 (s, 6H), 2.29 – 2.19 (m, 4H). **$^{13}$C NMR (126 MHz, Chloroform-*d*)** (for a pair of rotamers) δ 172.9, 172.7, 156.5, 156.4, 142.1, 142.1, 140.9 (2C), 139.8 (2C), 138.3, 138.3, 137.9, 137.5, 136.2, 136.1, 129.9 (2C), 129.4 (2C), 129.1 (2C), 128.2 (2C), 128.1 (2C), 127.8, 127.6, 126.6, 126.5, 125.9 (2C), 117.8, 117.7, 53.2 (2C), 50.7, 49.0, 48.9 (2C), 46.2, 46.0, 35.0, 34.1, 30.6, 30.3, 24.8, 24.6, 21.4 (2C), 20.0, 20.0, 12.4 (2C).

**4-(3,4-Dimethyl-7-oxo-2-(*p*-tolyl)-2,7-dihydro-6*H*-pyrazolo[3,4-d]pyridazin-6-yl)-*N*-(3-(fluoromethyl)benzyl)-*N*-phenethylbutanamide (3a)**

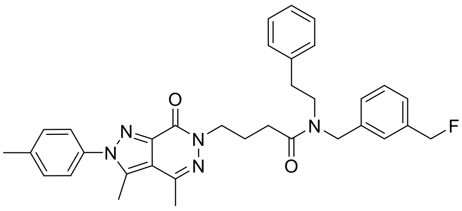

**$^1$H NMR (500 MHz, Chloroform-*d*)** (as a 1 : 0.9 mixture of rotamers) δ 7.38 – 7.30 (m, 10H), 7.30 – 7.22 (m, 8H), 7.22 – 7.12 (m, 4H), 7.10 – 7.05 (m, 4H), 5.35 (d, *J* = 47.8 Hz, 2H, major rotamer), 5.33 (d, *J* = 47.7 Hz, 2H, minor rotamer), 4.61 (s, 2H, major rotamer), 4.37 (s, 2H, minor rotamer), 4.31

– 4.17 (m, 4H), 3.58 – 3.53 (m, 2H, minor rotamer), 3.42 (t, $J$ = 7.5 Hz, 2H, major rotamer), 2.86 – 2.80 (m, 2H, minor rotamer), 2.78 (t, $J$ = 7.5 Hz, 2H, major rotamer), 2.61 (s, 3H, major rotamer), 2.60 (s, 3H, minor rotamer), 2.52 (s, 3H, minor rotamer), 2.51 (s, 3H, major rotamer), 2.45 (s, 6H), 2.44 – 2.40 (m, 2H, minor rotamer), 2.40 – 2.35 (m, 2H, major rotamer), 2.26 – 2.15 (m, 4H). **$^{13}$C NMR (126 MHz, Chloroform-*d*)** (for the major rotamer) δ 172.7, 156.5, 144.6, 143.2, 139.4 (2C), 136.1 (4C), 130.0, 129.0, 128.9 (2C), 128.8, 128.6 (2C), 126.8 (2C), 125.9 (4C), 117.8, 84.0, 49.1, 48.7, 40.1, 35.1, 30.1, 24.8, 21.4, 20.0, 12.4. **$^{19}$F NMR (470 MHz, Chloroform-*d*)** δ -207.04 (t, $J$ = 47.8 Hz), -207.80 (t, $J$ = 47.8 Hz).

***N*-(3-(chloromethyl)benzyl)-4-(3,4-dimethyl-7-oxo-2-(*p*-tolyl)-2,7-dihydro-6*H*-pyrazolo[3,4-d]pyridazin-6-yl)-*N*-phenethylbutanamide (3b)**

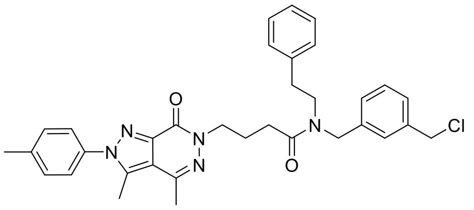

**$^{1}$H NMR (500 MHz, Chloroform-*d*)** (as a 1 : 0.9 mixture of rotamers) δ 7.37 – 7.29 (m, 10H), 7.29 – 7.23 (m, 6H), 7.21 – 7.14 (m, 6H), 7.10 – 7.03 (m, 4H), 4.58 (s, 2H, major rotamer), 4.56 (s, 2H, major rotamer), 4.53 (s, 2H, minor rotamer), 4.35 (s, 2H, minor rotamer), 4.26 – 4.19 (m, 4H), 3.58 – 3.51 (m, 2H, minor rotamer), 3.42 (t, $J$ = 7.5 Hz, 2H, major rotamer), 2.86 – 2.79 (m, 2H, minor rotamer), 2.77 (t, $J$ = 7.5 Hz, 2H, major rotamer), 2.60 (s, 3H, major rotamer), 2.60 (s, 3H, minor rotamer), 2.51 (s, 3H, minor rotamer), 2.51 (s, 3H, major rotamer), 2.44 (s, 6H), 2.43 – 2.35 (m, 4H), 2.25 – 2.15 (m, 4H). **$^{13}$C NMR (126 MHz, Chloroform-*d*)** (for a pair of rotamers) δ 172.8, 172.7, 156.4 (2C), 142.1, 142.1, 141.9, 140.9, 139.8, 139.4, 138.6, 138.3, 137.9, 137.8, 136.2, 136.1, 130.0 (4C), 129.4 (2C), 129.1, 129.0, 128.8 (2C), 128.8 (2C), 128.6 (2C), 128.2, 128.1, 127.8, 127.7, 126.8 (4C), 126.6, 126.5, 126.4, 125.9, 123.3 (2C), 123.2 (2C), 117.8, 117.8, 51.7, 49.1, 48.9, 48.9, 48.7, 48.3, 46.2, 46.0, 35.1, 34.1, 30.6, 30.1, 26.1, 25.8, 24.8, 24.7, 21.4, 20.0, 12.4 (2C).

**4-(3,4-Dimethyl-7-oxo-2-(*p*-tolyl)-2,7-dihydro-6*H*-pyrazolo[3,4-d]pyridazin-6-yl)-*N*,*N*-bis(3-(fluoromethyl)benzyl)butanamide (4a)**

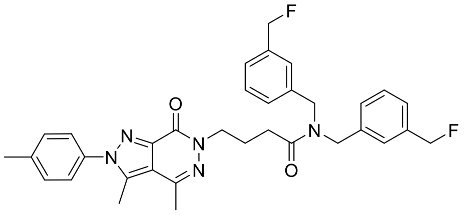

**$^{1}$H NMR (500 MHz, Chloroform-*d*)** δ 7.37 – 7.30 (m, 4H), 7.13 (s, 1H), 6.96 – 6.65 (m, 7H), 4.69 (s, 2H), 4.62 (s, 4H), 4.53 (s, 2H), 4.34 (t, $J$ = 6.7 Hz, 2H), 2.76 – 2.68 (m, 2H), 2.61 (s, 3H), 2.54 (s, 3H), 2.45 (s, 3H), 2.39 – 2.34 (m, 2H). **$^{13}$C NMR (126 MHz, Chloroform-*d*)** δ 172.9, 156.5, 142.2, 141.0, 139.9, 138.1, 138.0, 136.8, 136.2, 136.1, 135.2, 133.5, 131.6, 130.0 (2C), 128.3 (2C), 127.8 (2C), 127.5, 127.0, 126.5, 125.9, 117.8, 75.5, 75.1, 53.2, 51.3, 49.1, 30.8, 25.1, 21.4, 20.0, 12.4. **$^{19}$F NMR (470 MHz, Chloroform-*d*)** δ -207.15 (t, $J$ = 47.9 Hz), -207.98 (t, $J$ = 47.0 Hz).

**N,N-bis(3-(chloromethyl)benzyl)-4-(3,4-dimethyl-7-oxo-2-(p-tolyl)-2,7-dihydro-6H-pyrazolo[3,4-d]pyridazin-6-yl)butanamide (4b)**

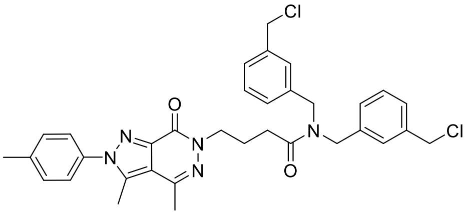

**$^1$H NMR (500 MHz, Chloroform-*d*)** δ 7.39 – 7.27 (m, 8H), 7.23 – 7.15 (m, 2H), 7.13 – 7.06 (m, 2H), 4.60 (s, 2H), 4.56 (s, 2H), 4.56 (s, 2H), 4.45 (s, 2H), 4.26 (t, *J* = 6.7 Hz, 2H), 2.61 (s, 3H), 2.57 – 2.52 (m, 2H), 2.52 (s, 3H), 2.45 (s, 3H), 2.33 – 2.23 (m, 2H). **$^{13}$C NMR (126 MHz, Chloroform-*d*)** δ 173.2, 156.5, 142.1, 141.0, 139.9, 138.4, 138.1, 138.0, 137.3, 136.2, 136.1, 130.0 (2C), 129.5, 129.2, 128.5, 128.4, 127.9, 127.8, 126.8, 126.7, 125.9 (2C), 117.8, 50.1, 49.0, 48.3, 46.3, 46.1, 30.5, 24.9, 21.4, 20.0, 12.4.

**N-(3,5-bis(fluoromethyl)benzyl)-4-(3,4-dimethyl-7-oxo-2-(p-tolyl)-2,7-dihydro-6H-pyrazolo[3,4-d]pyridazin-6-yl)butanamide (5)**

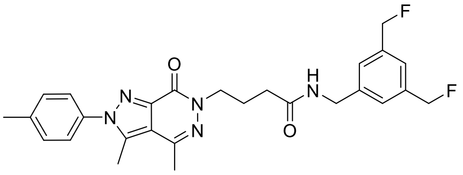

**$^1$H NMR (500 MHz, Chloroform-*d*)** δ 7.58 (s, 1H, amide NH), 7.39 – 7.26 (m, 7H), 5.37 (d, *J* = 46.7 Hz, 2H), 5.26 (d, *J* = 46.7 Hz, 2H), 4.50 (d, *J* = 6.0 Hz, 2H), 4.23 (t, *J* = 5.9 Hz, 2H), 2.63 (s, 3H), 2.53 (s, 3H), 2.44 (s, 3H), 2.31 – 2.24 (m, 2H), 2.23 – 2.14 (m, 2H). **$^{13}$C NMR (126 MHz, Chloroform-*d*)** δ 173.0, 157.1, 142.0, 141.9, 140.1, 139.4, 137.9 (2C), 136.6, 136.0, 130.0, 129.0, 127.9 (2C), 126.5, 125.9 (2C), 117.9, 84.7, 83.1, 48.6, 43.5, 33.5, 25.9, 21.4, 20.0, 12.3.

**4-(3,4-Dimethyl-7-oxo-2-(p-tolyl)-2,7-dihydro-6H-pyrazolo[3,4-d]pyridazin-6-yl)-N-((5-(fluoromethyl)-2,6-dimethylpyridin-3-yl)methyl)butanamide (6)**

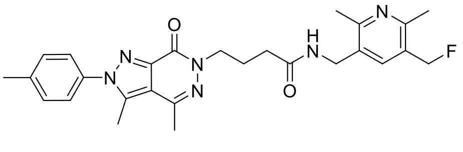

**$^1$H NMR (400 MHz, Chloroform-*d*)** δ 8.37 (s, 1H, amide NH), 7.38 – 7.32 (m, 5H), 5.47 (d, *J* = 46.8 Hz, 2H), 4.51 (d, *J* = 5.8 Hz, 2H), 4.20 (t, *J* = 6.1 Hz, 2H), 2.88 (s, 3H), 2.78 (s, 3H), 2.65 (s, 3H), 2.59 (s, 3H), 2.46 (s, 3H), 2.28 – 2.15 (m, 2H), 1.38 – 1.25 (m, 2H). **$^{13}$C NMR (126 MHz, Chloroform-*d*)** δ 173.1, 155.4 (3C), 142.5 (2C), 140.2, 137.7 (2C), 136.0, 129.8 (4C), 125.1 (2C), 117.4, 82.5, 42.6 (2C), 32.6, 26.1, 23.0, 20.5, 18.7 (2C), 11.7.

***N,N*-bis((5-(chloromethyl)-2,6-dimethylpyridin-3-yl)methyl)-4-(3,4-dimethyl-7-oxo-2-(*p*-tolyl)-2,7-dihydro-6*H*-pyrazolo[3,4-d]pyridazin-6-yl)butanamide (7)**

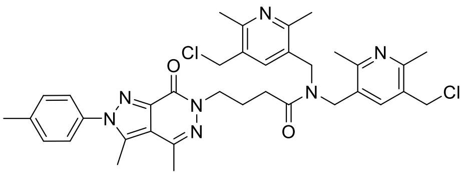

**$^{1}$H NMR (500 MHz, DMSO-*d*$_6$)** δ 7.9 – 7.27 (m, 6H), 4.64 (s, 2H), 4.62 (s, 2H), 4.53 (s, 2H), 4.47 (s, 2H), 4.20 (t, *J* = 6.0 Hz, 2H), 2.62 (s, 6H), 2.61 (s, 3H), 2.59 (s, 6H), 2.52 (s, 3H), 2.43 (s, 3H), 2.55 – 2.44 (m, 2H), 1.40 – 1.25 (m, 2H). **$^{13}$C NMR (126 MHz, DMSO-*d*$_6$)** δ 173.3, 156.9 (4C), 155.0, 142.3, 141.0, 139.5, 138.4, 137.5, 136.2, 135.5, 130.0 (2C), 129.4, 128.9, 126.7 (2C), 125.9 (2C), 117.8, 49.7, 48.6, 46.1, 45.5, 43.2, 30.5, 24.7, 21.4 (2C), 19.9 (4C), 11.9.

**4-((4-(3,4-Dimethyl-7-oxo-2-(*p*-tolyl)-2,7-dihydro-6*H*-pyrazolo[3,4-d]pyridazin-6-yl)-*N*-((5-(fluoromethyl)-2,6-dimethylpyridin-3-yl)methyl)butanamido)methyl)piperidin-1-ium 2,2,2-trifluoroacetate (8a)**

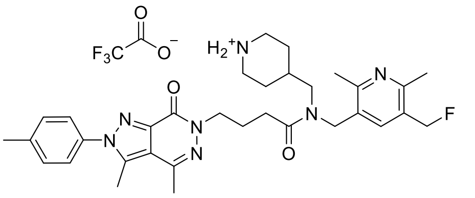

**$^{1}$H NMR (400 MHz, Chloroform-*d*)** δ 9.06 (s, 1H), 8.42 (s, 1H), 7.43 – 7.28 (m, 5H), 5.62 (d, *J* = 47.4 Hz, 2H), 4.55 (s, 2H), 4.29 – 4.17 (m, 4H), 4.17 – 4.06 (m, 2H), 3.30 – 3.16 (m, 2H), 2.76 (s, 3H), 2.71 (s, 3H), 2.61 (s, 3H), 2.57 (s, 3H), 2.46 (s, 3H), 2.45 – 2.42 (m, 2H), 2.26 – 2.19 (m, 2H), 1.66 – 1.59 (m, 2H), 1.27 – 1.24 (m, 1H), 1.12 – 1.05 (m, 2H). **$^{13}$C NMR (151 MHz, DMSO-*d*$_6$)** (for a pair of rotamers) δ 172.6, 172.6, 159.0, 158.8, 158.5, 158.3, 155.6, 155.5, 152.6, 152.2, 141.5 (2C), 140.9 (2C), 139.6 (2C), 137.7, 136.0, 135.9 (2C), 133.4, 133.2, 130.1 (8C), 125.9 (2C), 125.8 (2C), 117.3 (2C), 117.2 (2C), 80.9, 79.8, 52.8, 52.6, 48.4, 48.3, 45.6, 45.2, 43.1, 43.0, 33.1 (2C), 32.5 (2C), 32.1, 32.0, 29.1 (2C), 29.1 (2C), 24.1, 24.0, 21.1 (2C), 20.8 (2C), 19.7 (2C), 19.5 (2C), 11.9, 11.9. **$^{19}$F NMR (565 MHz, DMSO-*d*$_6$)** δ -74.68, -212.24 (t, *J* = 46.9 Hz), -212.88 (t, *J* = 46.9 Hz).

**4-((*N*-((5-(chloromethyl)-2,6-dimethylpyridin-3-yl)methyl)-4-(3,4-dimethyl-7-oxo-2-(*p*-tolyl)-2,7-dihydro-6*H*-pyrazolo[3,4-d]pyridazin-6-yl)butanamido)methyl)piperidin-1-ium 2,2,2-trifluoroacetate (8b)**

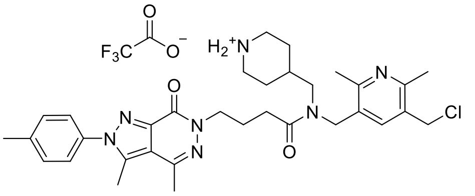

**$^{1}$H NMR (600 MHz, DMSO-*d*$_6$)** (as a 1: 0.9 mixture of rotamers) δ 8.73 (s, 1H), 8.66 (s, 1H), 8.42 (s, 1H), 8.37 (s, 1H), 8.20 (s, 1H), 8.05 (s, 1H), 7.46 – 7.43 (m, 8H), 5.01 (s, 4H), 4.63 (s, 2H), 4.61 (s, 2H), 4.10 (t, *J* = 6.8 Hz, 2H), 4.04 – 4.01 (m, 2H), 3.31 – 3.22 (m, 8H), 2.86 – 2.75 (m, 4H), 2.67 (s, 3H), 2.66 (s, 3H), 2.63 (s, 3H), 2.61 (s, 3H), 2.59 (s, 3H), 2.59 (s, 3H), 2.52 (s, 3H), 2.52 (s, 3H), 2.42 (s, 6H), 2.24 (t, *J* = 7.0 Hz, 4H), 2.04 – 1.95 (m, 4H), 1.80 – 1.72 (m, 4H), 1.39 – 1.26 (m, 6H). **$^{13}$C NMR (151 MHz, DMSO-*d*$_6$)** (for a pair

of rotamers) δ 172.7, 172.6, 159.0, 158.8, 158.5, 158.3, 155.7, 155.6, 151.5, 150.8, 141.5 (2C), 141.3 (2C), 139.6, 139.6, 137.8, 136.0, 135.9 (2C), 133.6, 133.1, 130.1 (8C), 125.9 (4C), 117.3 (2C), 117.2 (2C), 52.8, 52.6, 48.4, 48.3, 45.8, 45.6, 43.2, 43.1, 41.5, 41.4, 33.1 (2C), 32.1 (2C), 29.2 (2C), 28.8 (4C), 24.1 (2C), 24.0 (2C), 20.9 (2C), 19.5 (2C), 19.4 (2C), 11.9 (2C).

**4-((5-(3,4-Dimethyl-7-oxo-2-(*p*-tolyl)-2,7-dihydro-6*H*-pyrazolo[3,4-d]pyridazin-6-yl)-*N*-((5-(fluoromethyl)-2,6-dimethylpyridin-3-yl)methyl)pentanamido)methyl)piperidin-1-ium 2,2,2-trifluoroacetate (9a)**

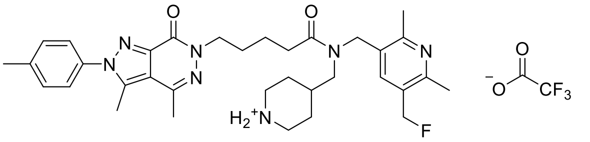

**$^1$H NMR (600 MHz, DMSO-$d_6$)** (as a 1: 0.7 mixture of rotamers) δ 8.79 (s, 2H), 8.70 (s, 2H), 7.97 (s, 1H), 7.88 (s, 1H), 7.46 (d, $J$ = 8.3 Hz, 4H), 7.42 (d, $J$ = 8.3 Hz, 4H), 5.64 (d, $J$ = 46.6 Hz, 2H, minor rotamer), 5.61 (d, $J$ = 46.6 Hz, 2H, major rotamer), 4.71 (s, 2H), 4.56 (s, 2H), 4.05 (t, $J$ = 6.9 Hz, 2H), 3.97 (t, $J$ = 6.9 Hz, 2H), 3.36 – 3.20 (m, 8H), 2.86 – 2.76 (m, 4H), 2.70 (s, 6H), 2.63 (s, 6H), 2.58 (s, 6H), 2.49 (s, 6H), 2.41 (s, 6H), 2.03 – 1.64 (m, 14H), 1.59 – 1.30 (m, 8H). **$^{13}$C NMR (151 MHz, DMSO-$d_6$)** (for a pair of rotamers) δ 173.8, 173.2, 159.2, 158.9, 158.7, 158.5, 155.5, 155.4, 151.4 (2C), 141.5 (2C), 141.3 (2C), 139.6 (2C), 137.7 (2C), 136.1 (2C), 133.2, 131.9, 130.1 (8C), 125.9 (4C), 117.3 (2C), 117.2 (2C), 80.8, 79.7, 52.7, 52.3, 48.7, 48.5, 45.5, 45.4, 43.2, 43.1, 33.2, 33.0, 31.7, 31.6, 28.0, 27.9, 26.3, 26.3, 23.2 (2C), 21.1 (4C), 20.8 (2C), 19.7 (2C), 19.5 (2C), 12.0 (2C). **$^{19}$F NMR (565 MHz, DMSO-$d_6$)** δ -74.92, -214.02 (t, $J$ = 46.3 Hz), -214.26 (t, $J$ = 46.8 Hz).

**4-((*N*-((5-(chloromethyl)-2,6-dimethylpyridin-3-yl)methyl)-5-(3,4-dimethyl-7-oxo-2-(*p*-tolyl)-2,7-dihydro-6*H*-pyrazolo[3,4-d]pyridazin-6-yl)pentanamido)methyl)piperidin-1-ium 2,2,2-trifluoroacetate (9b)**

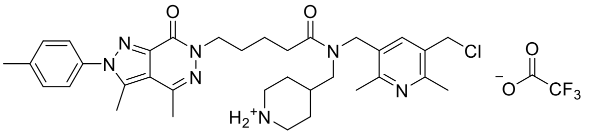

**$^1$H NMR (600 MHz, DMSO-$d_6$)** (as a 1: 0.8 mixture of rotamers) δ 8.72 (s, 1H), 8.65 (s, 1H), 8.41 (s, 1H), 8.35 (s, 1H), 8.01 (s, 1H), 7.97 (s, 1H), 7.47 (d, $J$ = 8.4 Hz, 4H), 7.43 (d, $J$ = 8.3 Hz, 4H), 4.98 (s, 2H), 4.94 (s, 2H), 4.56 (s, 2H), 4.54 (s, 2H), 4.06 (t, $J$ = 7.1 Hz, 2H), 4.01 – 3.95 (m, 2H), 3.38 – 3.20 (m, 8H), 2.89 – 2.76 (m, 4H), 2.71 (s, 3H), 2.67 (s, 3H), 2.66 (s, 3H), 2.65 (s, 3H), 2.59 (s, 3H), 2.58 (s, 3H), 2.51 (s, 6H), 2.42 (s, 6H), 2.26 (t, $J$ = 7.2 Hz, 2H), 2.00 – 1.91 (m, 2H), 1.83 – 1.60 (m, 10H), 1.59 – 1.46 (m, 4H), 1.43 – 1.26 (m, 4H). **$^{13}$C NMR (151 MHz, DMSO-$d_6$)** (for a pair of rotamers) δ 173.1, 173.0, 159.0, 158.7, 158.5, 158.2, 155.4, 155.3, 151.6, 150.8, 141.2 (2C), 141.2 (2C), 139.6 (2C), 137.7, 137.7, 136.0 (2C), 134.3, 132.9, 130.0 (8C), 125.8 (4C), 117.2 (2C), 117.2 (2C), 52.8, 52.6, 48.7, 48.4, 45.5, 45.4, 43.2, 43.1, 41.4, 41.4, 33.2 (2C), 31.7, 31.5, 27.9, 27.9, 26.3, 26.2, 21.9 (6C), 20.8 (2C), 19.5 (2C), 19.5 (2C), 12.0 (2C).

**4-((6-(3,4-Dimethyl-7-oxo-2-(*p*-tolyl)-2,7-dihydro-6*H*-pyrazolo[3,4-d]pyridazin-6-yl)-*N*-((5-(fluoromethyl)-2,6-dimethylpyridin-3-yl)methyl)hexanamido)methyl)piperidin-1-ium 2,2,2-trifluoroacetate (10a)**

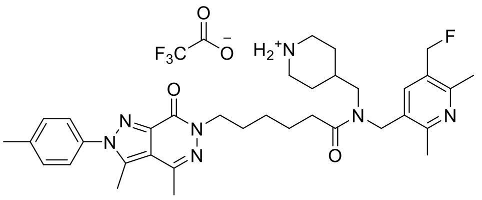


**$^1$H NMR (600 MHz, DMSO-$d_6$)** (as a 1: 0.8 mixture of rotamers) δ 8.77 (s, 2H), 8.70 (s, 2H), 7.95 (s, 1H), 7.84 (s, 1H), 7.46 (d, $J$ = 8.3 Hz, 5H), 7.42 (d, $J$ = 8.3 Hz, 8H), 5.65 (d, $J$ = 46.6 Hz, 2H, minor rotamer), 5.63 (d, $J$ = 46.6 Hz, 2H, major rotamer), 4.69 (s, 2H), 4.56 (s, 2H), 4.06 – 4.01 (m, 2H), 4.01 – 3.95 (m, 2H), 3.34 – 3.20 (m, 8H), 2.86 – 2.76 (m, 3H), 2.67 (s, 3H), 2.66 (s, 3H), 2.65 (s, 3H), 2.65 (s, 3H), 2.58 (s, 3H), 2.57 (s, 3H), 2.51 (s, 6H), 2.42 (s, 6H), 1.79 – 1.50 (m, 14H), 1.42 – 1.20 (m, 12H). **$^{13}$C NMR (151 MHz, DMSO-$d_6$)** (for a pair of rotamers) δ 173.3, 173.2, 159.1, 158.8, 158.6, 158.4, 155.4, 155.3, 151.4 (2C), 141.3 (2C), 141.2 (2C), 139.6 (2C), 137.7, 137.7, 136.0 (2C), 133.2, 131.5, 130.1 (8C), 125.9 (4C), 117.3 (2C), 117.3 (2C), 80.9, 79.8, 52.7, 51.9, 48.9, 48.8, 45.4, 45.3, 43.2, 43.0, 33.1 (2C), 32.0, 31.3, 28.4, 28.3, 26.3, 26.2, 26.1, 25.9, 24.7 (2C), 24.6 (2C), 21.1 (2C), 20.8 (2C), 19.5 (2C), 19.5 (2C), 12.0 (2C). **$^{19}$F NMR (565 MHz, DMSO-$d_6$)** δ -74.75, -214.16 (t, $J$ = 47.8 Hz).

**4-((*N*-((5-(chloromethyl)-2,6-dimethylpyridin-3-yl)methyl)-6-(3,4-dimethyl-7-oxo-2-(*p*-tolyl)-2,7-dihydro-6*H*-pyrazolo[3,4-d]pyridazin-6-yl)hexanamido)methyl)piperidin-1-ium 2,2,2-trifluoroacetate (10b)**

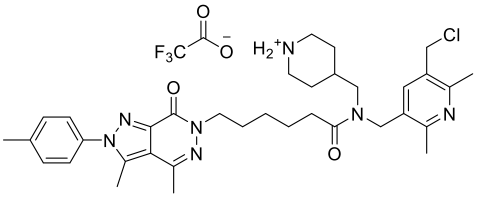


**$^1$H NMR (600 MHz, DMSO-$d_6$)** (as a 1: 0.8 mixture of rotamers) δ 8.74 (s, 1H), 8.66 (s, 1H), 8.37 (s, 2H), 8.02 (s, 1H), 7.96 (s, 1H), 7.46 (d, $J$ = 8.4 Hz, 4H), 7.42 (d, $J$ = 8.3 Hz, 4H), 4.99 (s, 2H), 4.96 (s, 2H), 4.61 (s, 2H), 4.60 (s, 2H), 4.03 (t, $J$ = 7.2 Hz, 2H), 4.00 – 3.95 (m, 2H), 3.34 – 3.19 (m, 8H), 2.88 – 2.77 (m, 4H), 2.66 (s, 3H), 2.65 (s, 3H), 2.59 (s, 3H), 2.58 (s, 6H), 2.57 (s, 3H), 2.51 (s, 6H), 2.41 (s, 6H), 2.20 (t, $J$ = 7.3 Hz, 4H), 1.80 – 1.67 (m, 10H), 1.68 – 1.49 (m, 4H), 1.42 – 1.19 (m, 8H). **$^{13}$C NMR (151 MHz, DMSO-$d_6$)** δ 173.3, 173.3, 159.0, 158.8, 158.5, 158.3, 155.4, 155.3, 150.9, 150.8, 141.3 (2C), 141.3 (2C), 139.6 (2C), 137.7 (2C), 136.0 (2C), 133.6, 133.0, 130.1 (8C), 125.9 (4C), 117.3 (2C), 117.3 (2C), 52.8, 52.6, 49.0, 48.9, 45.5, 45.4, 43.1, 43.1, 41.6, 41.4, 33.1, 33.1, 32.0, 32.0, 28.4 (2C), 26.2, 26.0, 25.8 (2C), 24.8 (2C), 24.7 (2C), 20.9 (4C), 19.5 (2C), 19.5 (2C), 12.0 (2C).

**4-((7-(3,4-Dimethyl-7-oxo-2-(*p*-tolyl)-2,7-dihydro-6*H*-pyrazolo[3,4-d]pyridazin-6-yl)-*N*-((5-(fluoromethyl)-2,6-dimethylpyridin-3-yl)methyl)heptanamido)methyl)piperidin-1-ium 2,2,2-trifluoroacetate (11a)**

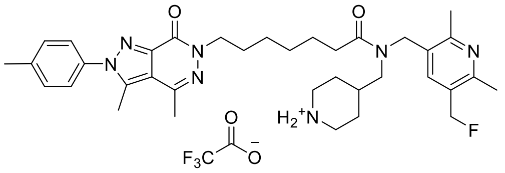


**$^1$H NMR (600 MHz, DMSO-$d_6$)** (as a 1: 0.6 mixture of rotamers) δ 8.80 (s, 2H), 8.70 (s, 2H), 7.97 (s, 1H), 7.87 (s, 1H), 7.46 (d, *J* = 8.4 Hz, 4H), 7.41 (d, *J* = 8.3 Hz, 4H), 5.65 (d, *J* = 46.6 Hz, 2H, minor rotamer), 5.62 (d, *J* = 46.6 Hz, 2H, major rotamer), 4.69 (d, *J* = 11.0 Hz, 2H), 4.56 (s, 2H), 4.03 (t, *J* = 7.2 Hz, 2H), 3.98 (t, *J* = 7.2 Hz, 2H), 3.35 – 3.18 (m, 8H), 2.88 – 2.74 (m, 4H), 2.68 (s, 6H), 2.67 (s, 6H), 2.58 (s, 6H), 2.50 (s, 6H), 2.41 (s, 6H), 1.78 – 1.63 (m, 8H), 1.59 – 1.44 (m, 6H), 1.36 – 1.20 (m, 16H). **$^{13}$C NMR (151 MHz, DMSO-$d_6$)** (for a pair of rotamers) δ 173.5, 173.4, 159.2, 158.9, 158.7, 158.5, 155.4, 155.4, 151.7, 150.5, 141.3 (2C), 141.3 (2C), 139.6 (2C), 137.7, 137.7, 136.1 (2C), 134.1, 131.4, 130.1 (8C), 125.9 (4C), 117.3 (4C), 80.7, 79.6, 52.7, 52.0, 49.0, 48.9, 45.4 (2C), 43.2, 43.1, 33.1 (2C), 32.1, 32.0, 28.5, 28.4, 26.3, 26.2, 26.1 (2C), 26.1 (2C), 24.9 (2C), 24.8 (2C), 20.8 (4C), 19.5 (2C), 19.5 (2C), 12.0 (2C). **$^{19}$F NMR (565 MHz, DMSO-$d_6$)** δ -74.97, -214.49 – -214.77 (m).

**4-((*N*-((5-(chloromethyl)-2,6-dimethylpyridin-3-yl)methyl)-7-(3,4-dimethyl-7-oxo-2-(*p*-tolyl)-2,7-dihydro-6*H*-pyrazolo[3,4-d]pyridazin-6-yl)heptanamido)methyl)piperidin-1-ium 2,2,2-trifluoroacetate (11b)**

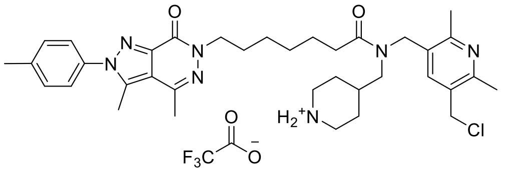


**$^1$H NMR (700 MHz, DMSO-$d_6$)** (as a 1: 0.8 mixture of rotamers) δ 8.76 (s, 1H), 8.67 (s, 1H), 8.42 (s, 1H), 8.37 (s, 1H), 8.03 (s, 1H), 7.99 (s, 1H), 7.45 (d, *J* = 8.3 Hz, 4H), 7.41 (d, *J* = 8.3 Hz, 4H), 5.00 (s, 2H), 4.95 (s, 2H), 4.59 (s, 2H), 4.54 (s, 2H), 4.02 (t, *J* = 7.2 Hz, 2H), 3.98 (t, *J* = 7.3 Hz, 2H), 3.34 – 3.19 (m, 8H), 3.04 – 2.76 (m, 4H), 2.72 (s, 6H), 2.66 (s, 6H), 2.57 (s, 6H), 2.49 (s, 6H), 2.41 (s, 6H), 1.79 – 1.61 (m, 8H), 1.61 – 1.43 (m, 6H), 1.41 – 1.18 (m, 16H). **$^{13}$C NMR (176 MHz, DMSO-$d_6$)** (for a pair of rotamers) δ 173.4, 173.3, 159.0, 158.8, 158.6, 158.4, 155.4, 155.3, 152.2, 151.6, 150.9, 150.8, 141.3 (2C), 141.2 (2C), 139.6 (2C), 137.7, 137.6, 136.0 (2C), 134.4, 133.0, 130.0 (8C), 125.9 (4C), 117.3 (2C), 117.3 (2C), 52.8, 51.9, 49.0, 48.9, 45.4, 45.3, 43.2, 43.1, 41.4, 41.3, 33.1 (2C), 32.1 (2C), 28.5, 28.4, 26.3, 26.2, 26.1 (2C), 26.1 (2C), 24.8 (2C), 24.7 (2C), 20.8 (4C), 19.5 (4C), 12.0 (2C).

**4-(((3-(3,4-Dimethyl-7-oxo-2-(*p*-tolyl)-2,7-dihydro-6*H*-pyrazolo[3,4-d]pyridazin-6-yl)-*N*-((5-(fluoromethyl)-2,6-dimethylpyridin-3-yl)methyl)propyl)sulfonamido)methyl)piperidin-1-ium 2,2,2-trifluoroacetate (12a)**

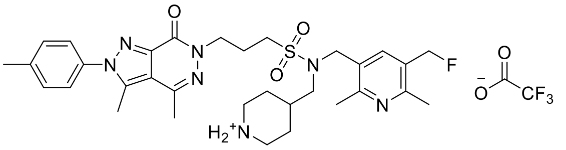

**$^1$H NMR (400 MHz, DMSO-$d_6$)** δ 8.65 (s, 1H), 8.32 (s, 1H), 8.03 (s, 1H), 7.52 – 7.40 (m, 4H), 5.60 (d, $J$ = 47.0 Hz, 2H), 4.52 (s, 2H), 4.19 (t, $J$ = 7.1 Hz, 2H), 3.39 – 3.29 (m, 2H), 3.24 – 3.13 (m, 4H), 2.74 – 2.62 (m, 2H), 2.61 (s, 6H), 2.58 (s, 3H), 2.53 (s, 3H), 2.43 (s, 3H), 2.22 – 2.08 (m, 2H), 1.77 – 1.64 (m, 2H), 1.55 – 1.41 (m, 1H), 1.26 – 1.11 (m, 2H). **$^{13}$C NMR (126 MHz, DMSO-$d_6$)** δ 158.4, 158.2, 155.3 (2C), 141.5, 141.1, 139.4, 137.6, 135.8 (2C), 129.9 (4C), 125.7 (2C), 117.2 (2C), 81.5, 80.2, 54.3, 49.1, 47.6, 47.1, 42.6 (2C), 32.4 (2C), 26.1 (2C), 22.4, 20.7, 19.4 (3C), 11.9. **$^{19}$F NMR (470 MHz, DMSO-$d_6$)** δ -74.30, -212.84.

**4-(((*N*-((5-(chloromethyl)-2,6-dimethylpyridin-3-yl)methyl)-3-(3,4-dimethyl-7-oxo-2-(*p*-tolyl)-2,7-dihydro-6*H*-pyrazolo[3,4-d]pyridazin-6-yl)propyl)sulfonamido)methyl)piperidin-1-ium 2,2,2-trifluoroacetate (12b)**

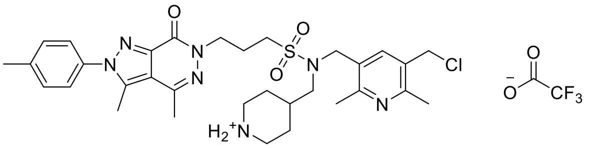

**$^1$H NMR (600 MHz, DMSO-$d_6$)** δ 8.66 (s, 1H), 8.35 (s, 1H), 8.05 (s, 1H), 7.48 (d, $J$ = 8.4 Hz, 2H), 7.44 (d, $J$ = 8.2 Hz, 2H), 4.93 (s, 2H), 4.49 (s, 2H), 4.19 (t, $J$ = 7.1 Hz, 2H), 3.36 – 3.29 (m, 2H), 3.23 – 3.14 (m, 4H), 2.71 – 2.63 (m, 2H), 2.63 (s, 3H), 2.61 (s, 3H), 2.59 (s, 3H), 2.53 (s, 3H), 2.43 (s, 3H), 2.21 – 2.10 (m, 2H), 1.71 (d, $J$ = 12.6 Hz, 2H), 1.45 – 1.35 (m, 1H), 1.23 – 1.14 (m, 2H). **$^{13}$C NMR (151 MHz, DMSO-$d_6$)** δ 158.4, 158.2, 155.3 (2C), 141.5, 141.1, 139.4, 137.6, 135.9 (2C), 129.9 (4C), 125.7 (2C), 117.2 (2C), 54.5, 49.2, 47.6, 47.0, 42.7 (2C), 32.5 (2C), 26.1 (2C), 22.4, 20.8, 19.4 (3C), 11.9.

**4-(((4-(3,4-Dimethyl-7-oxo-2-(*p*-tolyl)-2,7-dihydro-6*H*-pyrazolo[3,4-d]pyridazin-6-yl)-*N*-((5-(fluoromethyl)-2,6-dimethylpyridin-3-yl)methyl)butyl)sulfonamido)methyl)piperidin-1-ium 2,2,2-trifluoroacetate (13a)**

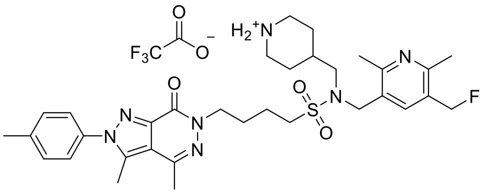

**$^1$H NMR (400 MHz, DMSO-$d_6$)** δ 8.60 (s, 1H), 8.28 (s, 1H), 7.97 (s, 1H), 7.53 – 7.39 (m, 4H), 5.57 (d, $J$ = 47.1 Hz, 2H), 4.46 (s, 2H), 4.11 (t, $J$ = 6.7 Hz, 2H), 3.37 – 3.25 (m, 2H), 3.19 (d, $J$ = 12.6 Hz, 2H), 3.12 (d, $J$ = 7.2 Hz, 2H), 2.72 – 2.62 (m, 2H), 2.60 (s, 3H), 2.58 – 2.57 (m, 3H), 2.56 (s, 3H), 2.53 (s, 3H), 2.43 (s, 3H), 1.93 – 1.80 (m, 2H), 1.78 – 1.64 (m, 4H), 1.51 – 1.36 (m, 1H), 1.22 – 1.07 (m, 2H). **$^{13}$C NMR (126 MHz, DMSO-$d_6$)** δ 158.4, 158.1, 155.3 (2C), 141.2, 141.1, 139.4, 137.6, 135.9 (2C), 129.9 (4C), 125.7 (2C),

117.1 (2C), 81.7, 80.4, 54.3, 49.3, 48.7, 48.0, 42.6 (2C), 32.5 (2C), 27.0, 26.1, 20.7 (2C), 20.0 (2C), 19.4 (2C), 11.9. **$^{19}$F NMR (470 MHz, DMSO-*d*$_6$)** δ -74.22, -212.15.

**4-(((*N*-((5-(chloromethyl)-2,6-dimethylpyridin-3-yl)methyl)-4-(3,4-dimethyl-7-oxo-2-(*p*-tolyl)-2,7-dihydro-6*H*-pyrazolo[3,4-d]pyridazin-6-yl)butyl)sulfonamido)methyl)piperidin-1-ium 2,2,2-trifluoroacetate (13b)**

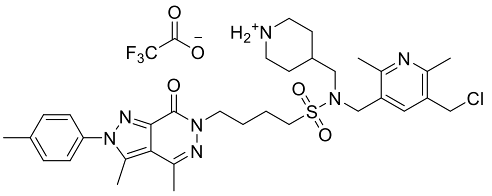


**$^1$H NMR (600 MHz, DMSO-*d*$_6$)** δ 8.68 (s, 1H), 8.37 (s, 1H), 8.09 (s, 1H), 7.41 (d, *J* = 8.3 Hz, 2H), 7.36 (d, *J* = 8.2 Hz, 2H), 4.89 (s, 2H), 4.43 (s, 2H), 3.98 (t, *J* = 7.2 Hz, 2H), 3.21 – 3.04 (m, 6H), 2.61 (s, 3H), 2.59 – 2.57 (m, 1H), 2.56 (s, 3H), 2.56 – 2.55 (m, 1H), 2.53 (s, 3H), 2.45 (s, 3H), 2.36 (s, 3H), 1.72 – 1.59 (m, 6H), 1.43 – 1.35 (m, 2H), 1.35 – 1.30 (m, 1H), 1.30 – 1.23 (m, 2H), 1.19 – 1.07 (m, 2H). **$^{13}$C NMR (151 MHz, DMSO-*d*$_6$)** δ 158.6, 158.3, 155.2 (2C), 141.1, 141.0, 139.4, 137.5, 135.9 (2C), 129.9 (4C), 125.7 (2C), 117.1 (2C), 58.3, 54.6, 49.1, 48.7, 42.6, 42.3, 32.5, 28.0, 27.4, 26.1, 25.5 (2C), 22.6 (2C), 20.7 (2C), 11.9.

**4-(((5-(3,4-Dimethyl-7-oxo-2-(*p*-tolyl)-2,7-dihydro-6*H*-pyrazolo[3,4-d]pyridazin-6-yl)-*N*-((5-(fluoromethyl)-2,6-dimethylpyridin-3-yl)methyl)pentyl)sulfonamido)methyl)piperidin-1-ium 2,2,2-trifluoroacetate (14a)**

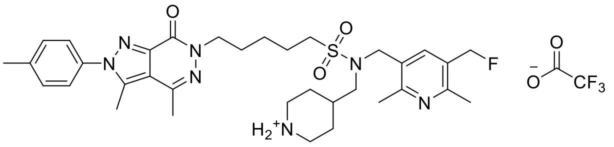


**$^1$H NMR (500 MHz, DMSO-*d*$_6$)** δ 8.66 (s, 1H), 8.32 (s, 1H), 8.07 (s, 1H), 7.48 (d, *J* = 8.4 Hz, 2H), 7.43 (d, *J* = 8.3 Hz, 2H), 5.60 (d, *J* = 46.9 Hz, 2H), 4.50 (s, 2H), 4.06 (t, *J* = 7.0 Hz, 2H), 3.31 – 3.09 (m, 6H), 2.75 – 2.63 (m, 3H), 2.62 (s, 3H), 2.60 (s, 3H), 2.59 (s, 3H), 2.52 (s, 3H), 2.43 (s, 3H), 1.80 – 1.66 (m, 6H), 1.49 – 1.37 (m, 3H), 1.21 – 1.11 (m, 2H). **$^{13}$C NMR (126 MHz, DMSO-*d*$_6$)** δ 158.5, 158.2, 155.2 (2C), 141.1, 141.0, 139.4, 137.5, 135.9 (2C), 129.9 (4C), 125.7 (2C), 117.1 (2C), 81.4, 80.2, 54.4, 49.1, 49.0, 48.5, 42.6 (2C), 32.5 (2C), 27.8, 26.1, 24.8 (2C), 22.4 (2C), 20.7, 19.4 (2C), 11.9. **$^{19}$F NMR (470 MHz, DMSO-*d*$_6$)** δ -74.29, -213.06.

**4-(((*N*-((5-(chloromethyl)-2,6-dimethylpyridin-3-yl)methyl)-5-(3,4-dimethyl-7-oxo-2-(*p*-tolyl)-2,7-dihydro-6*H*-pyrazolo[3,4-d]pyridazin-6-yl)pentyl)sulfonamido)methyl)piperidin-1-ium 2,2,2-trifluoroacetate (14b)**

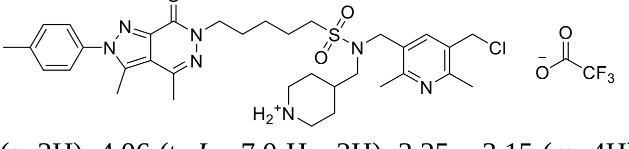


**$^1$H NMR (600 MHz, DMSO-*d*$_6$)** δ 8.52 (s, 1H), 8.22 (s, 1H), 7.99 (s, 1H), 7.48 (d, *J* = 8.4 Hz, 2H), 7.44 (d, *J* = 8.3 Hz, 2H), 4.91 (s, 2H), 4.45 (s, 2H), 4.06 (t, *J* = 7.0 Hz, 2H), 3.25 – 3.15 (m, 4H), 3.12 (d, *J* = 7.3 Hz, 2H), 2.70 – 2.61 (m, 2H),

2.60 (s, 6H), 2.56 (s, 3H), 2.53 (s, 3H), 2.43 (s, 3H), 1.79 – 1.67 (m, 6H), 1.46 – 1.38 (m, 2H), 1.38 – 1.31 (m, 1H), 1.20 – 1.10 (m, 2H). **$^{13}$C NMR (151 MHz, DMSO-*d*$_6$)** δ 158.3, 158.0, 155.2 (2C), 141.1, 141.0, 139.4, 137.5, 135.9 (2C), 129.9 (4C), 125.7 (2C), 117.1 (2C), 54.5, 49.3, 48.9, 48.5, 42.7 (2C), 32.6 (2C), 27.8, 26.1, 24.8 (2C), 22.4 (2C), 20.7, 19.4 (2C), 11.9.

**4-(((6-(3,4-Dimethyl-7-oxo-2-(*p*-tolyl)-2,7-dihydro-6*H*-pyrazolo[3,4-d]pyridazin-6-yl)-*N*-((5-(fluoromethyl)-2,6-dimethylpyridin-3-yl)methyl)hexyl)sulfonamido)methyl)piperidin-1-ium 2,2,2-trifluoroacetate (15a)**

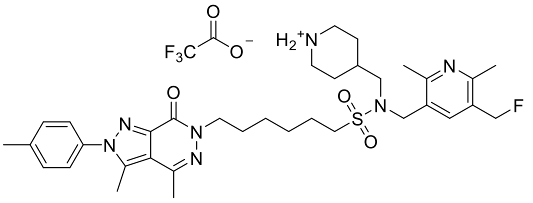


**$^1$H NMR (500 MHz, DMSO-*d*$_6$)** δ 8.49 (s, 1H), 8.20 (s, 1H), 7.91 (d, *J* = 9.0 Hz, 1H), 7.48 (d, *J* = 8.4 Hz, 2H), 7.43 (d, *J* = 8.2 Hz, 2H), 5.55 (d, *J* = 47.2 Hz, 2H), 4.44 (d, *J* = 9.9 Hz, 2H), 4.05 (t, *J* = 7.2 Hz, 2H), 3.21 – 3.09 (m, 6H), 2.71 – 2.61 (m, 2H), 2.59 (s, 3H), 2.56 (s, 3H), 2.53 (s, 3H), 2.52 (s, 3H), 2.43 (s, 3H), 1.76 – 1.65 (m, 6H), 1.50 – 1.37 (m, 3H), 1.38 – 1.30 (m, 3H), 1.19 – 1.08 (m, 2H). **$^{13}$C NMR (126 MHz, DMSO-*d*$_6$)** δ 158.2, 157.9, 155.1 (2C), 141.1, 141.0, 139.4, 137.5, 135.9 (2C), 129.9 (4C), 125.7 (2C), 117.1 (2C), 82.6, 49.1, 48.6, 42.6 (2C), 32.5 (2C), 28.0, 27.4, 26.1, 25.5 (2C), 22.6 (2C), 20.7, 19.4 (2C), 11.9. **$^{19}$F NMR (470 MHz, DMSO-*d*$_6$)** δ -74.06, -211.02.

**4-(((*N*-((5-(chloromethyl)-2,6-dimethylpyridin-3-yl)methyl)-6-(3,4-dimethyl-7-oxo-2-(*p*-tolyl)-2,7-dihydro-6*H*-pyrazolo[3,4-d]pyridazin-6-yl)hexyl)sulfonamido)methyl)piperidin-1-ium 2,2,2-trifluoroacetate (15b)**

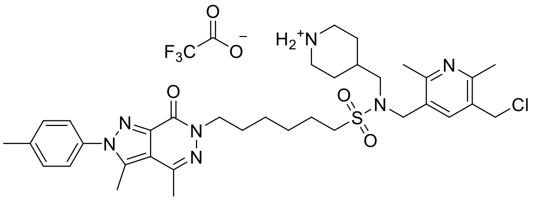


**$^1$H NMR (600 MHz, DMSO-*d*$_6$)** δ 8.57 (s, 1H), 8.27 (s, 1H), 7.96 (s, 1H), 7.48 (d, *J* = 8.3 Hz, 2H), 7.44 (d, *J* = 8.2 Hz, 2H), 4.90 (s, 2H), 4.44 (s, 2H), 4.11 (t, *J* = 6.8 Hz, 2H), 3.33 – 3.26 (m, 2H), 3.21 – 3.15 (m, 2H), 3.11 (d, *J* = 7.2 Hz, 2H), 2.68 – 2.61 (m, 2H), 2.60 (s, 6H), 2.55 (s, 3H), 2.53 (s, 3H), 2.43 (s, 3H), 1.86 (p, *J* = 7.0 Hz, 2H), 1.76 – 1.65 (m, 4H), 1.39 – 1.33 (m, 1H), 1.19 – 1.09 (m, 2H). **$^{13}$C NMR (151 MHz, DMSO-*d*$_6$)** δ 158.3, 158.0, 155.3 (2C), 141.2, 141.1, 139.4, 137.6, 135.9 (2C), 129.9 (4C), 125.7 (2C), 117.1 (2C), 54.5, 51.7, 49.4, 48.6, 48.1, 42.7 (2C), 32.6 (2C), 27.0, 26.1, 25.0 (2C), 20.7 (2C), 20.0, 19.4 (2C), 11.9.

**4-(((4-(*N*-(4-chlorobenzyl)-*N*-cyclopentylsulfamoyl)-*N*-((6-(chloromethyl)pyridin-2-yl)methyl)phenyl)sulfonamido)methyl)piperidin-1-ium 2,2,2-trifluoroacetate (16)**

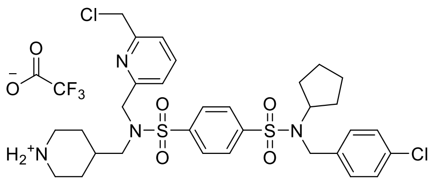

**$^{1}$H NMR (400 MHz, Chloroform-*d*) δ** 11.52 (s, 2H), 7.99 (t, *J* = 7.7 Hz, 1H), 7.91 (s, 4H), 7.67 (d, *J* = 7.7 Hz, 1H), 7.57 (d, *J* = 7.6 Hz, 1H), 7.32 – 7.27 (m, 4H), 4.66 (s, 2H), 4.58 (s, 2H), 4.34 (s, 2H), 4.32 – 4.23 (m, 1H), 3.48 – 3.27 (m, 1H), 3.27 – 3.10 (m, 2H), 2.94 – 2.60 (m, 2H), 2.19 (s, 2H), 1.90 – 1.73 (m, 2H), 1.69 – 1.59 (m, 2H), 1.58 – 1.37 (m, 6H), 1.37 – 1.23 (m, 3H). **$^{13}$C NMR (101 MHz, Chloroform-*d*)** δ 161.0, 155.3, 154.8, 145.3, 142.0, 141.7, 136.9, 133.4, 128.8 (2C), 128.6 (2C), 128.2 (2C), 128.2 (2C), 124.2, 124.1, 117.1, 60.0, 55.0, 53.6, 47.2, 44.1, 43.7, 32.8, 31.0, 29.6 (2C), 26.3 (2C), 23.4 (2C).

**4-(((4-(*N*-(4-chlorobenzyl)-*N*-cyclopentylsulfamoyl)-*N*-(3-(chloromethyl)-2,4,6-trimethylbenzyl)phenyl)sulfonamido)methyl)piperidin-1-ium 2,2,2-trifluoroacetate (17)**

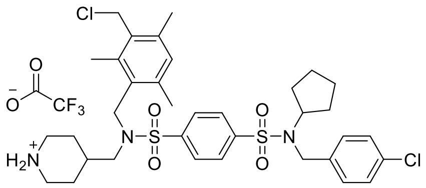

**$^{1}$H NMR (400 MHz, Chloroform-*d*)** δ 9.06 (s, 1H), 8.60 (s, 1H), 7.98 (d, *J* = 8.2 Hz, 2H), 7.91 (d, *J* = 8.2 Hz, 2H), 7.30 (d, *J* = 2.2 Hz, 4H), 6.88 (s, 1H), 4.64 (s, 2H), 4.37 (s, 2H), 4.28 (d, *J* = 7.8 Hz, 4H), 3.20 (d, *J* = 11.9 Hz, 2H), 2.74 (d, *J* = 6.6 Hz, 2H), 2.55 – 2.46 (m, 2H), 2.42 (s, 3H), 2.37 (s, 3H), 2.26 (s, 1H), 2.22 (s, 3H), 1.57 – 1.36 (m, 6H), 1.32 – 1.12 (m, 6H), 1.02 (d, *J* = 11.8 Hz, 1H). **$^{13}$C NMR (101 MHz, Chloroform-*d*)** δ 157.6, 145.0, 140.8, 138.6, 138.4, 138.3, 137.0, 136.9, 133.5, 133.4, 131.2 (2C), 128.9, 128.8, 128.6, 128.4 (2C), 128.1 (2C), 117.6, 59.9, 53.4, 48.9, 47.2, 43.7 (2C), 41.5, 34.5, 29.5 (2C), 26.5 (2C), 23.4 (2C), 20.6, 19.4, 15.5.

**4-(((4-(*N*-(4-chlorobenzyl)-*N*-cyclopentylsulfamoyl)-*N*-(3-(chloromethyl)-5-methoxybenzyl)phenyl)sulfonamido)methyl)piperidin-1-ium 2,2,2-trifluoroacetate (18)**

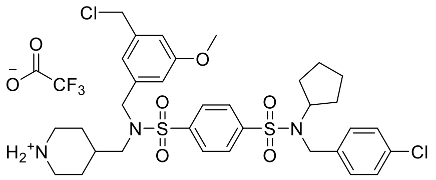

**$^{1}$H NMR (400 MHz, Chloroform-*d*)** δ 9.24 (s, 1H), 8.77 (s, 1H), 7.91 (q, *J* = 8.2 Hz, 4H), 7.28 (d, *J* = 1.8 Hz, 4H), 6.85 (d, *J* = 12.9 Hz, 2H), 6.67 (s, 1H), 4.50 – 4.45 (m, 2H), 4.35 (s, 2H), 4.32 – 4.25 (m, 1H), 4.23 (s, 2H), 3.75 (s, 3H), 3.29 (s, 2H), 3.02 (d, *J* = 6.5 Hz, 2H), 2.62 (s, 2H), 1.81 – 1.69 (m, 2H), 1.68 – 1.59 (m, 2H), 1.59 – 1.45 (m, 4H), 1.46 – 1.41 (m, 2H), 1.32 – 1.22 (m, 3H). **$^{13}$C NMR (101 MHz, Chloroform-*d*)** δ 160.3 (2C), 145.0, 142.7, 139.8, 137.7, 136.9, 133.3, 128.8 (2C), 128.6 (2C), 128.2 (2C), 128.0 (2C), 121.0 (2C), 114.4, 113.8, 59.9, 55.5, 54.5, 54.0, 47.1, 45.9, 43.7 (2C), 33.4, 29.5 (2C), 26.4 (2C), 23.4 (2C).

**4-(((4-(*N*-(4-chlorobenzyl)-*N*-cyclopentylsulfamoyl)-*N*-((6-(1-chloroethyl)pyridin-2-yl)methyl)phenyl)sulfonamido)methyl)piperidin-1-ium 2,2,2-trifluoroacetate (19)**

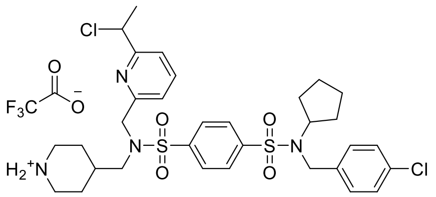

**$^1$H NMR (400 MHz, Chloroform-*d*)** (as a racemic mixture) δ 9.45 (s, 1H), 9.03 (s, 1H), 7.89 – 7.82 (m, 4H), 7.67 (t, *J* = 7.8 Hz, 1H), 7.38 – 7.27 (m, 6H), 4.94 (q, *J* = 6.8 Hz, 1H), 4.57 – 4.38 (m, 2H), 4.33 (s, 2H), 4.33 – 4.18 (m, 2H), 3.47 (q, *J* = 7.0 Hz, 1H), 3.32 (br.s, 2H), 3.20 (d, *J* = 6.9 Hz, 2H), 2.68 (s, 2H), 1.84 – 1.73 (m, 4H), 1.66 – 1.57 (m, 2H), 1.54 – 1.38 (m, 6H), 1.30 – 1.22 (m, 2H). **$^{13}$C NMR (101 MHz, Chloroform-*d*)** δ 160.6 (2C), 155.4, 144.8, 143.0, 138.1, 137.0, 133.3, 128.8, 128.6, 128.1 (2C), 128.0 (4C), 122.7 (2C), 120.7, 59.9, 58.6, 54.5, 54.4, 47.2, 43.6 (2C), 33.0, 29.5 (2C), 26.4 (2C), 24.4, 23.4 (2C).

**4-(((*N*-(3,5-bis(fluoromethyl)benzyl)-4-(*N*-(4-chlorobenzyl)-*N*-cyclopentylsulfamoyl)phenyl)sulfonamido)methyl)piperidin-1-ium 2,2,2-trifluoroacetate (20)**

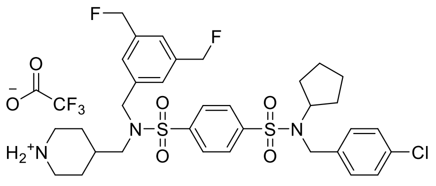

**$^1$H NMR (400 MHz, Chloroform-*d*)** δ 9.43 (s, 1H), 8.91 (s, 1H), 7.91 (q, *J* = 7.9 Hz, 4H), 7.29 (s, 5H), 7.26 (s, 3H), 5.40 (s, 2H), 5.28 (s, 2H), 4.36 (s, 2H), 4.32 (s, 2H), 4.30 – 4.25 (m, 1H), 3.26 (s, 2H), 3.02 (d, *J* = 6.9 Hz, 2H), 2.59 (s, 2H), 1.78 – 1.69 (m, 2H), 1.68 – 1.60 (m, 2H), 1.56 – 1.51 (m, 2H), 1.47 – 1.42 (m, 2H), 1.38 – 1.24 (m, 5H). **$^{13}$C NMR (101 MHz, Chloroform-*d*)** δ 158.0, 145.2, 142.7, 137.9 (2C), 137.7, 136.9, 133.4, 128.8, 128.7, 128.2 (4C), 128.1 (4C), 127.5, 117.6, 84.6, 83.0, 60.0, 54.6, 54.0, 47.2, 43.5 (2C), 33.7, 29.6 (2C), 26.4 (2C), 23.4 (2C).

***N*$^1$-(3-(*tert*-butyl)-5-(fluoromethyl)benzyl)-*N*$^4$-(4-chlorobenzyl)-*N*$^4$-cyclopentyl-*N*$^1$-(piperidin-4-ylmethyl)benzene-1,4-disulfonamide (21a)**

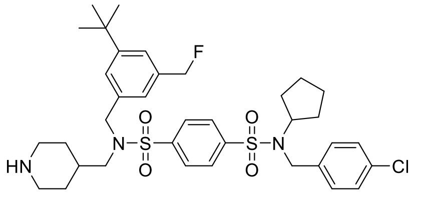

**$^1$H NMR (700 MHz, Chloroform-*d*)** *δ* 8.04 – 7.82 (m, 4H), 7.33 (s, 1H), 7.29 (s, 5H), 7.07 (s, 1H), 5.35 (d, *J* = 47.7 Hz, 2H), 4.39 (s, 1H), 4.32 (td, *J* = 19.3, 17.5, 10.7 Hz, 2H), 4.24 (d, *J* = 13.4 Hz, 1H), 3.04 (d, *J* = 7.5 Hz, 1H), 3.01 (s, 1H), 2.80 (t, *J* = 13.0 Hz, 1H), 2.62 (t, *J* = 12.7 Hz, 1H), 1.83 – 1.44 (m, 12H), 1.32 (s, 3H), 1.12 – 1.01 (m, 2H). **$^{13}$C NMR (176 MHz, Chloroform-*d*)** δ 148.0, 145.0, 143.4, 136.9 (2C), 135.9, 133.5, 128.9 (2C), 128.7 (2C), 128.2 (4C), 128.0 (2C), 125.0, 84.1, 60.0, 54.7, 54.3 (2C), 47.2 (2C), 42.8, 35.3, 34.9 (2C), 31.4 (3C), 29.6 (2C), 23.4 (2C).

**4-(((*N*-(3-(*tert*-butyl)-5-(chloromethyl)benzyl)-4-(*N*-(4-chlorobenzyl)-*N*-cyclopentylsulfamoyl)phenyl)sulfonamido)methyl)piperidin-1-ium 2,2,2-trifluoroacetate (21b)**

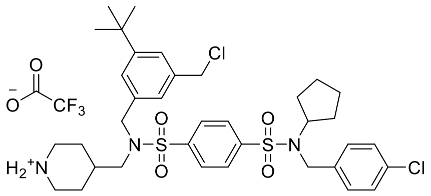

**$^{1}$H NMR (400 MHz, Chloroform-*d*)** *δ* 8.99 (s, 1H), 8.64 (s, 1H), 8.01 – 7.83 (m, 4H), 7.35 – 7.27 (m, 5H), 7.26 (s, 1H), 7.23 – 7.20 (m, 1H), 4.67 – 4.40 (m, 4H), 4.40 – 4.15 (m, 5H), 3.34 – 3.20 (m, 2H), 3.08 – 2.85 (m, 3H), 2.70 – 2.57 (m, 3H), 1.80 – 1.58 (m, 4H), 1.58 – 1.32 (m, 6H), 1.32 – 1.23 (m, 3H), 1.27 (s, 9H). **$^{13}$C NMR (101 MHz, Chloroform-*d*)** *δ* 161.3, 152.9, 152.5, 145.0, 142.5, 142.2, 138.1, 136.7, 135.9, 133.3, 128.7, 128.5, 128.1, 128.0, 127.9, 126.1, 125.8, 125.7, 59.8, 54.4, 47.1 (2C), 46.2, 43.7 (2C), 34.9, 34.8, 34.7, 33.4, 31.3 (2C), 31.2, 31.1, 29.5, 26.3, 26.0, 23.3 (2C).

**4-(((4-(*N*-(4-chlorobenzyl)-*N*-cyclopentylsulfamoyl)-*N*-((5-(fluoromethyl)-2,6-dimethylpyridin-3-yl)methyl)phenyl)sulfonamido)methyl)piperidin-1-ium 2,2,2-trifluoroacetate (22a)**

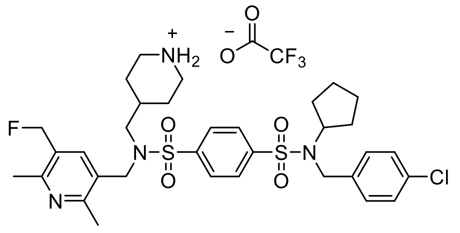

**$^{1}$H NMR (600 MHz, DMSO-*d*$_6$)** δ 8.54 (s, 1H), 8.22 (s, 1H), 8.07 (d, *J* = 8.4 Hz, 2H), 8.02 (d, *J* = 8.3 Hz, 2H), 7.69 (s, 1H), 7.43 (d, *J* = 8.5 Hz, 2H), 7.40 (d, *J* = 8.5 Hz, 2H), 5.47 (d, *J* = 47.3 Hz, 2H), 4.41 (s, 2H), 4.39 (s, 2H), 4.27 (p, *J* = 8.5 Hz, 1H), 3.20 (d, *J* = 12.4 Hz, 2H), 3.11 (d, *J* = 7.3 Hz, 2H), 2.66 (q, *J* = 11.8 Hz, 2H), 1.66 (d, *J* = 13.0 Hz, 2H), 1.58 – 1.41 (m, 5H), 1.42 – 1.30 (m, 2H), 1.27 – 1.09 (m, 4H). **$^{13}$C NMR (151 MHz, DMSO-*d*$_6$)** δ 158.2, 158.0, 155.1, 154.1, 143.8, 141.8, 138.4 (2C), 131.5 (2C), 128.7 (2C), 128.3, 128.2 (4C), 117.6, 115.6, 81.8, 80.8, 59.1, 54.3, 49.6, 46.2, 42.6 (2C), 32.5, 28.6 (2C), 26.0 (2C), 22.9 (2C), 20.5, 19.9. **$^{19}$F NMR (470 MHz, DMSO-*d*$_6$)** δ -74.34, -213.45. **HRMS:** calc. for $[M+H]^+$, $C_{33}H_{43}ClFN_4O_4S_2$ = 677.2393, found: 677.2392.

**4-(((4-(*N*-(4-chlorobenzyl)-*N*-cyclopentylsulfamoyl)-*N*-((5-(chloromethyl)-2,6-dimethylpyridin-3-yl)methyl)phenyl)sulfonamido)methyl)piperidin-1-ium 2,2,2-trifluoroacetate (22b)**

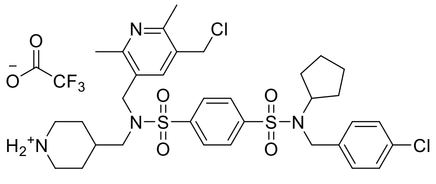

**$^{1}$H NMR (500 MHz, DMSO-*d*$_6$)** δ 8.72 (s, 1H), 8.40 (s, 1H), 8.16 – 8.03 (m, 5H), 7.48 – 7.37 (m, 4H), 4.91 (s, 2H), 4.44 (s, 2H), 4.42 (s, 2H), 4.33 – 4.23 (m, 1H), 3.21 (d, *J* = 13.0 Hz, 2H), 3.14 (d, *J* = 7.2 Hz, 2H), 2.65 (s, 3H), 2.57 (s, 3H), 1.76 – 1.65 (m, 2H), 1.55 – 1.42 (m, 5H), 1.42 – 1.30 (m, 2H), 1.27 – 1.14 (m, 4H). **$^{13}$C NMR (126 MHz, DMSO-*d*$_6$)** δ 158.6, 158.3, 153.4, 153.1, 143.9, 141.7, 138.4 (2C), 131.6 (2C), 128.7 (2C), 128.4, 128.3 (4C), 117.3, 115.0, 59.2, 54.9, 49.3 (2C), 46.2, 42.6 (2C), 32.5, 28.6 (2C), 26.1 (2C), 22.9 (2C), 18.9, 18.4.

Spectral Characterization for Representative Compounds

**$^{1}$H NMR** Spectrum of **12a** (400 MHz, DMSO-$d_6$)

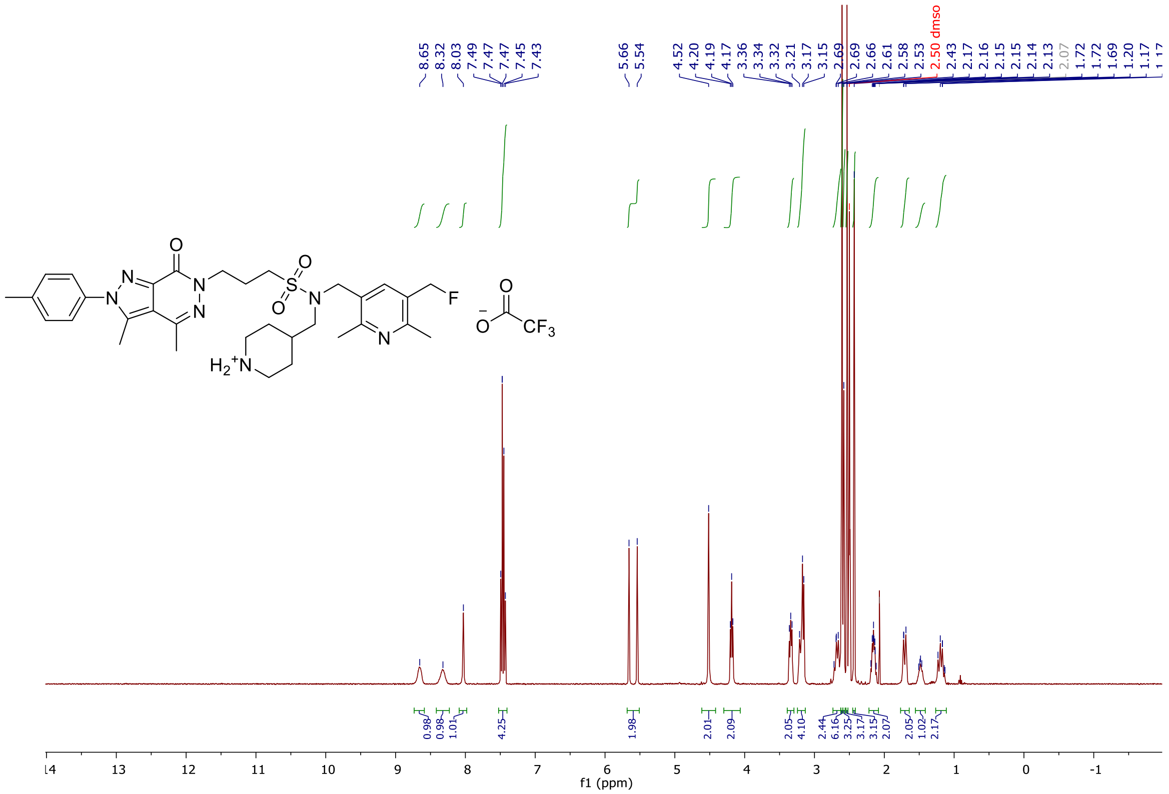

**$^{13}$C NMR** Spectrum of **12a** (126 MHz, DMSO-$d_6$)

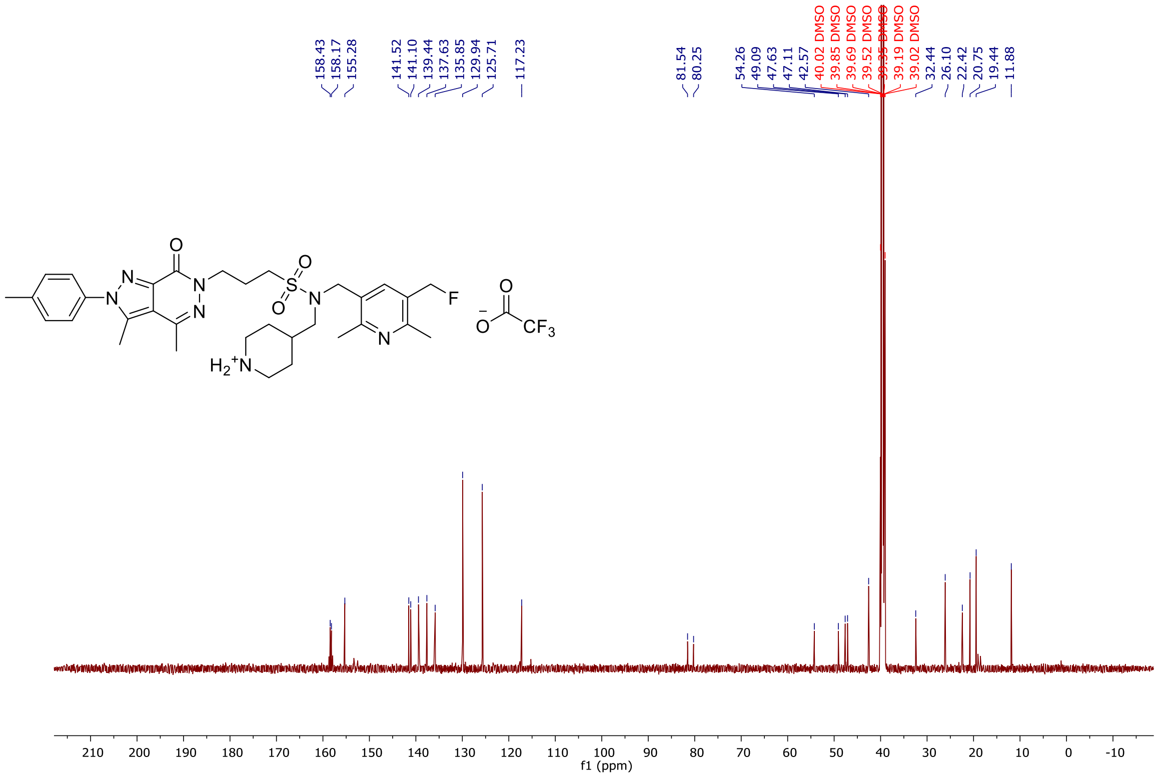

$^{1}$**H NMR** Spectrum of **12b** (600 MHz, DMSO-$d_6$)

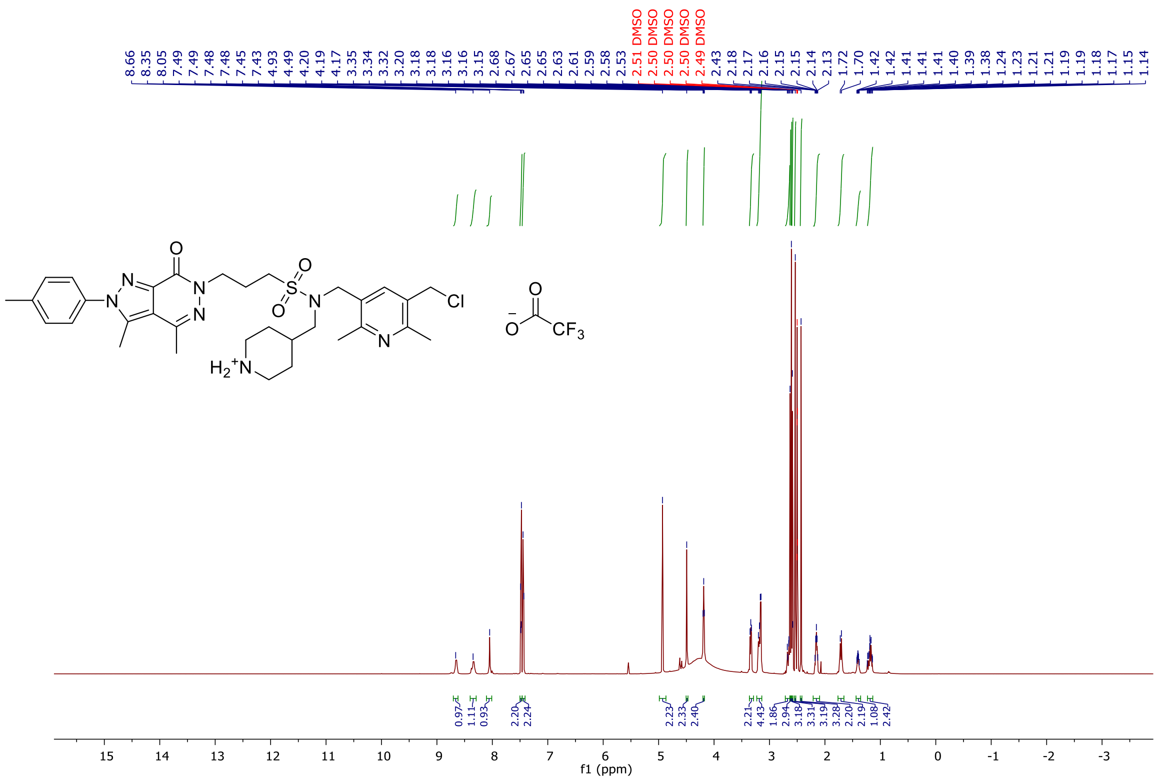

$^{13}$**C NMR** Spectrum of **12b** (151 MHz, DMSO-$d_6$)

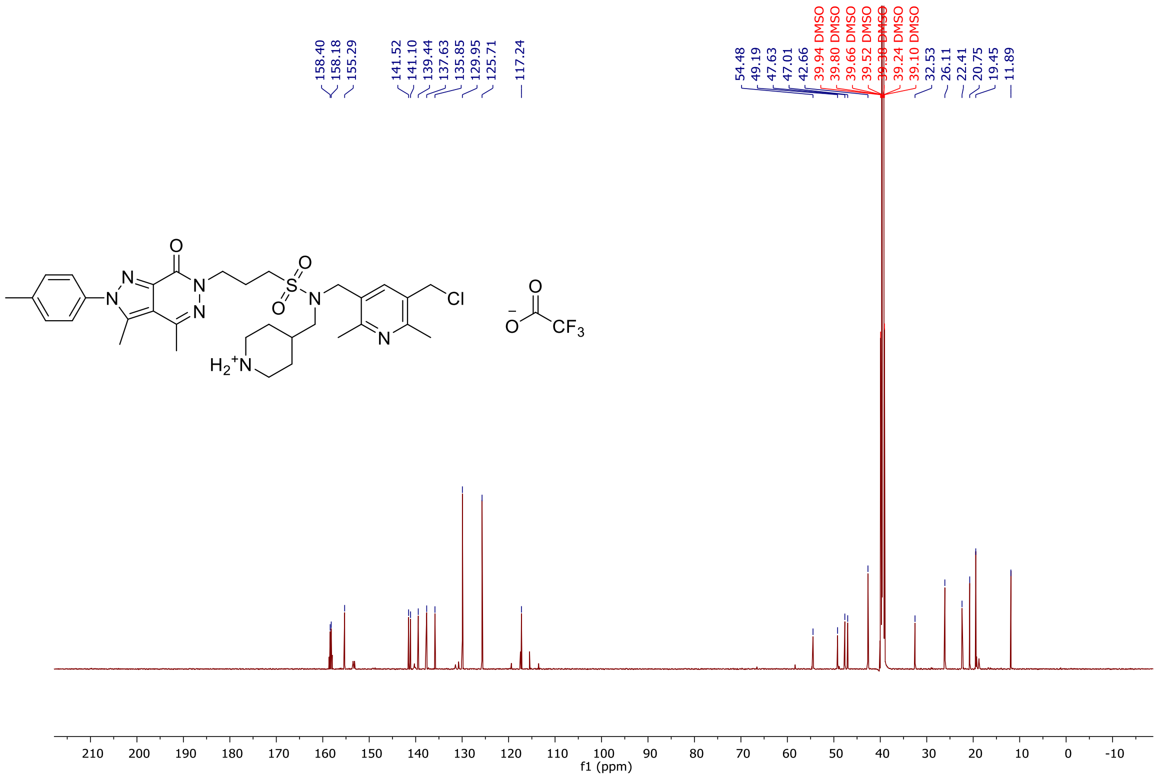

**$^{1}$H NMR** Spectrum of **13a** (400 MHz, DMSO-$d_6$)

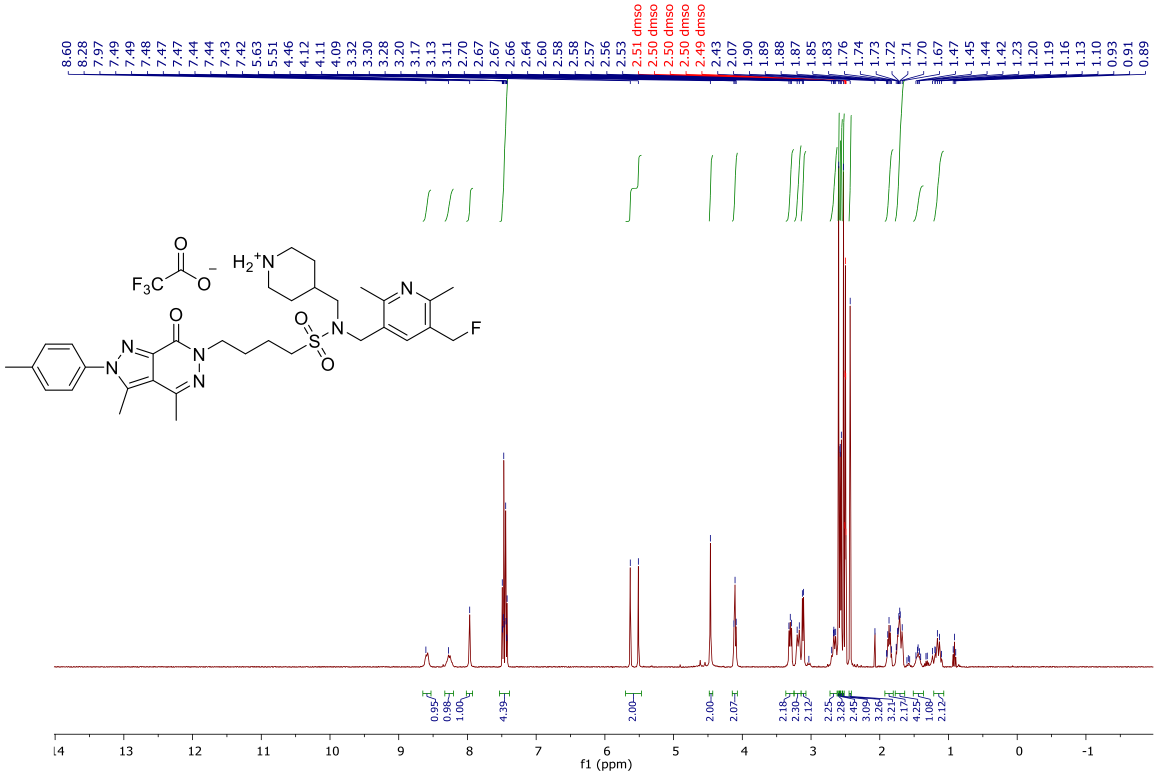

**$^{13}$C NMR** Spectrum of **13a** (126 MHz, DMSO-$d_6$)

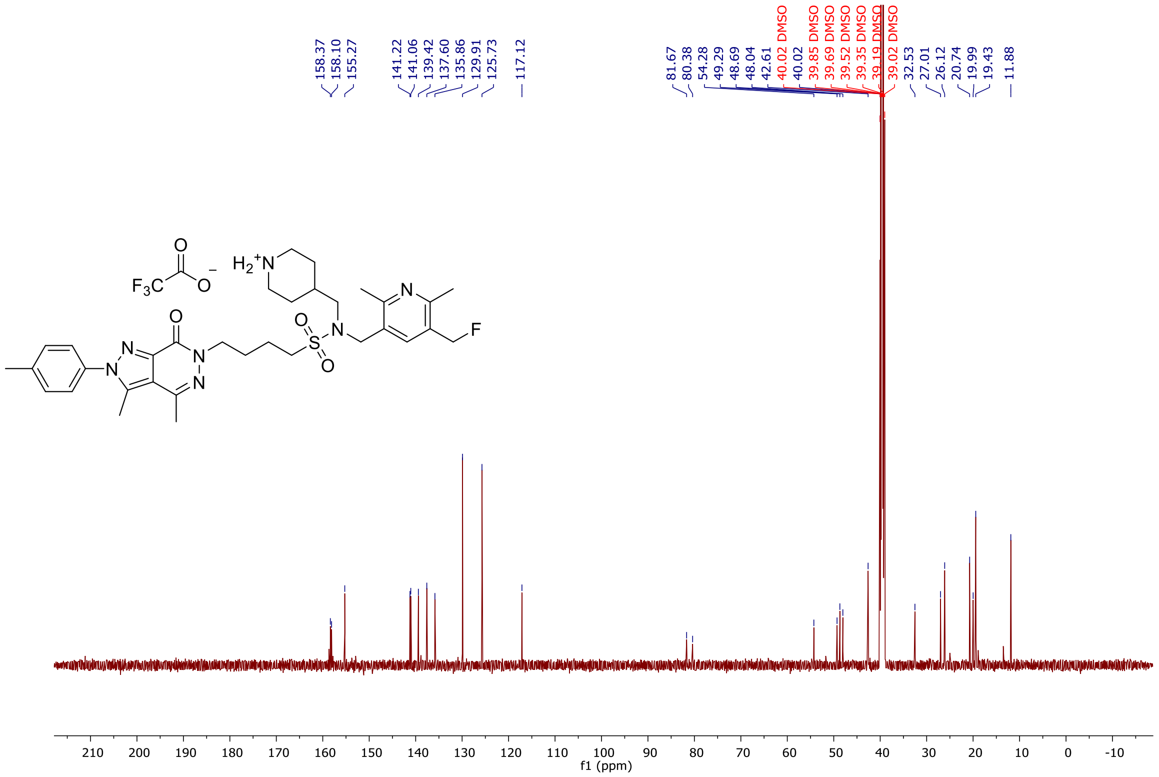

**$^{1}$H NMR** Spectrum of **13b** (600 MHz, DMSO-$d_6$)

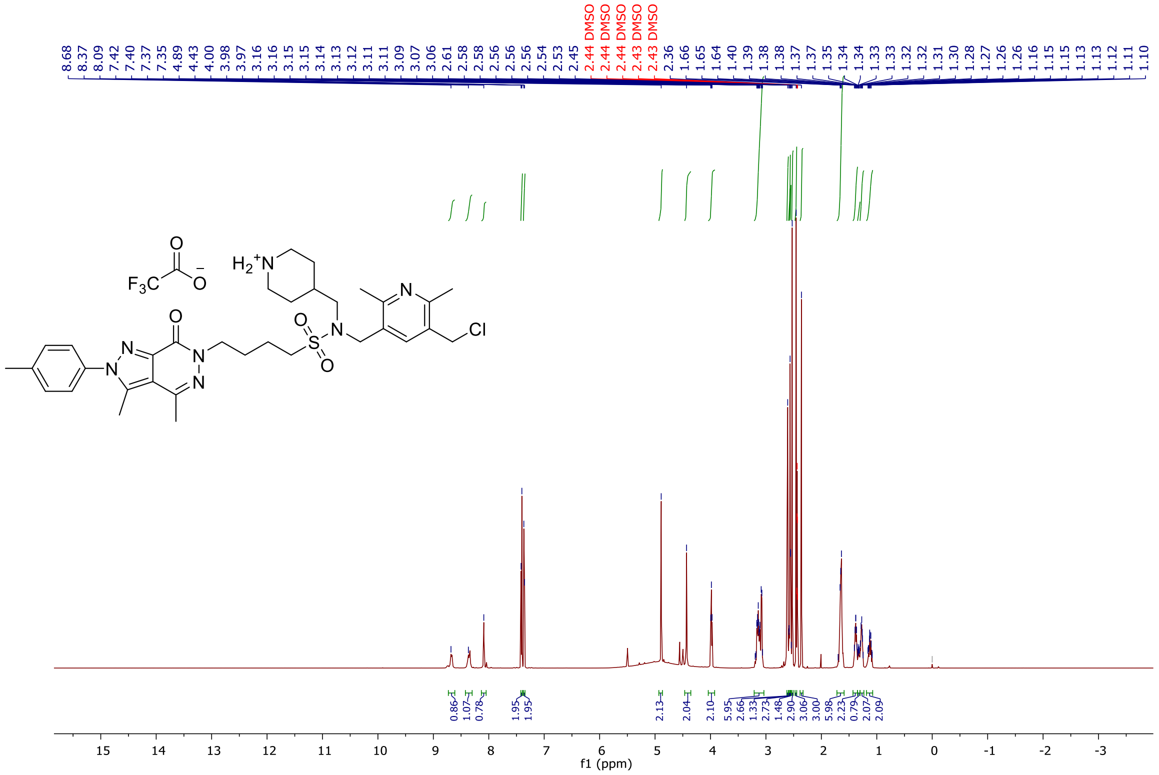


**$^{13}$C NMR** Spectrum of **13b** (151 MHz, DMSO-$d_6$)

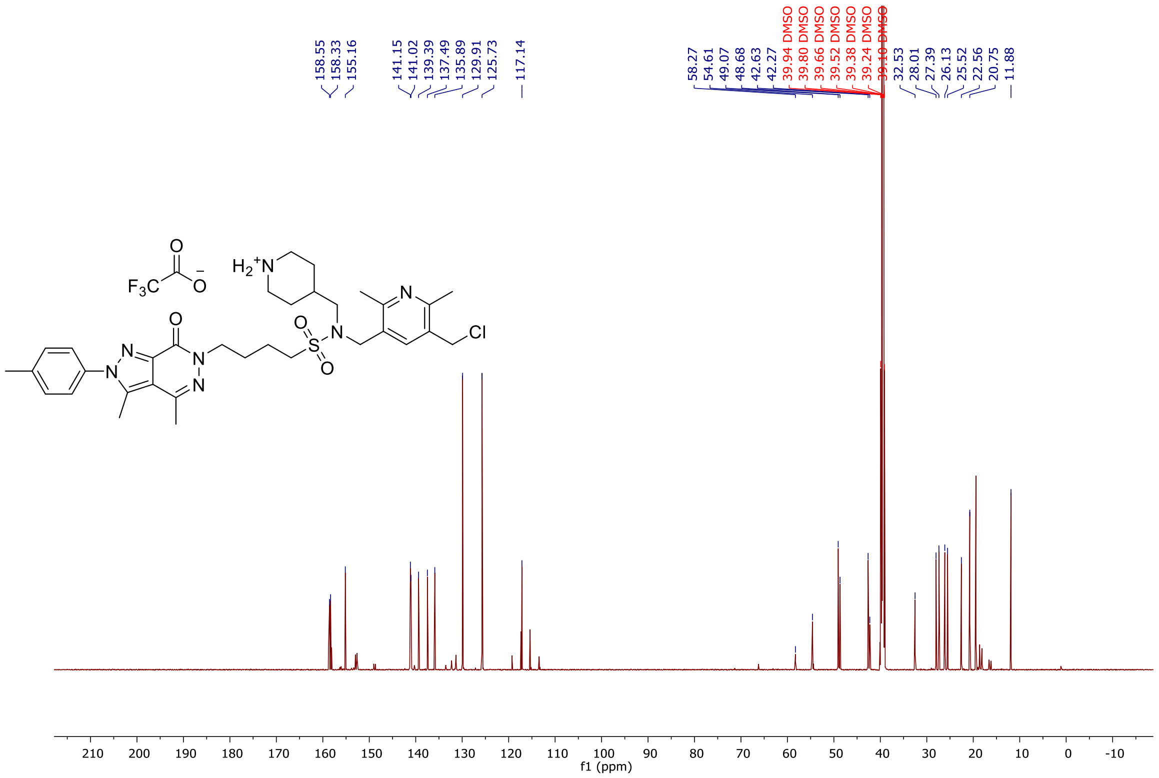

**$^{1}$H NMR** Spectrum of **14a** (500 MHz, DMSO-$d_6$)

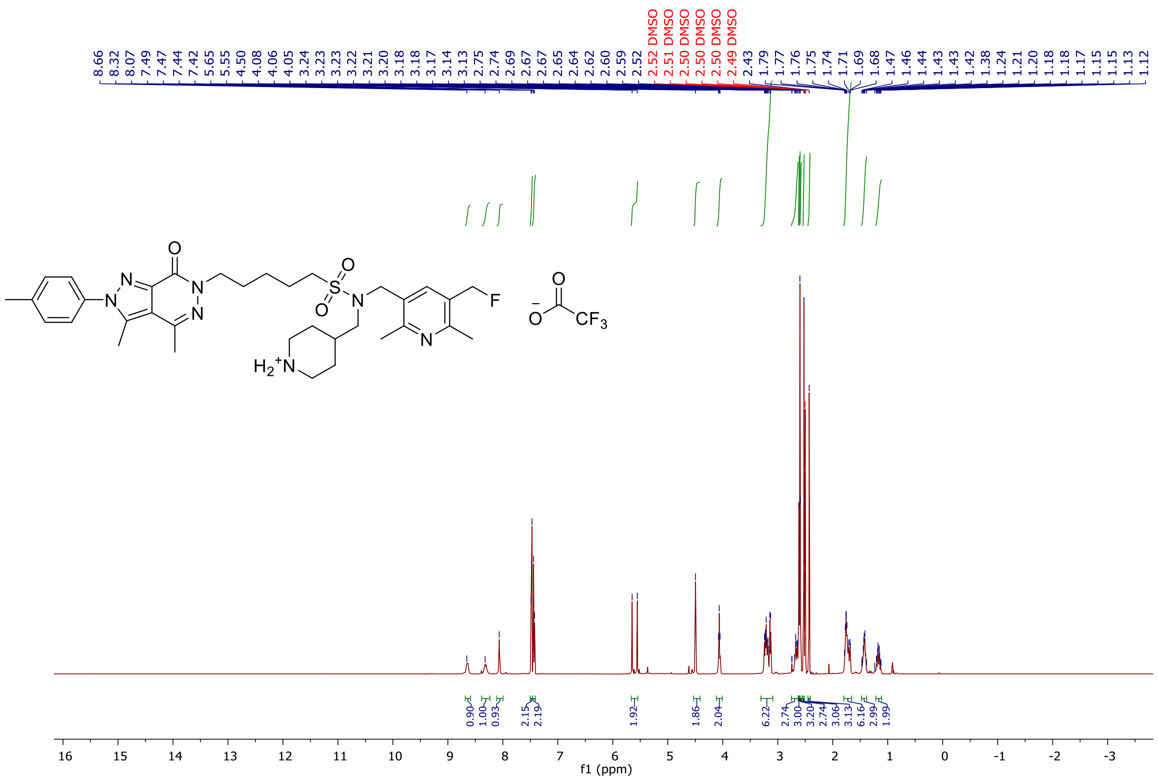

**$^{13}$C NMR** Spectrum of **14a** (126 MHz, DMSO-$d_6$)

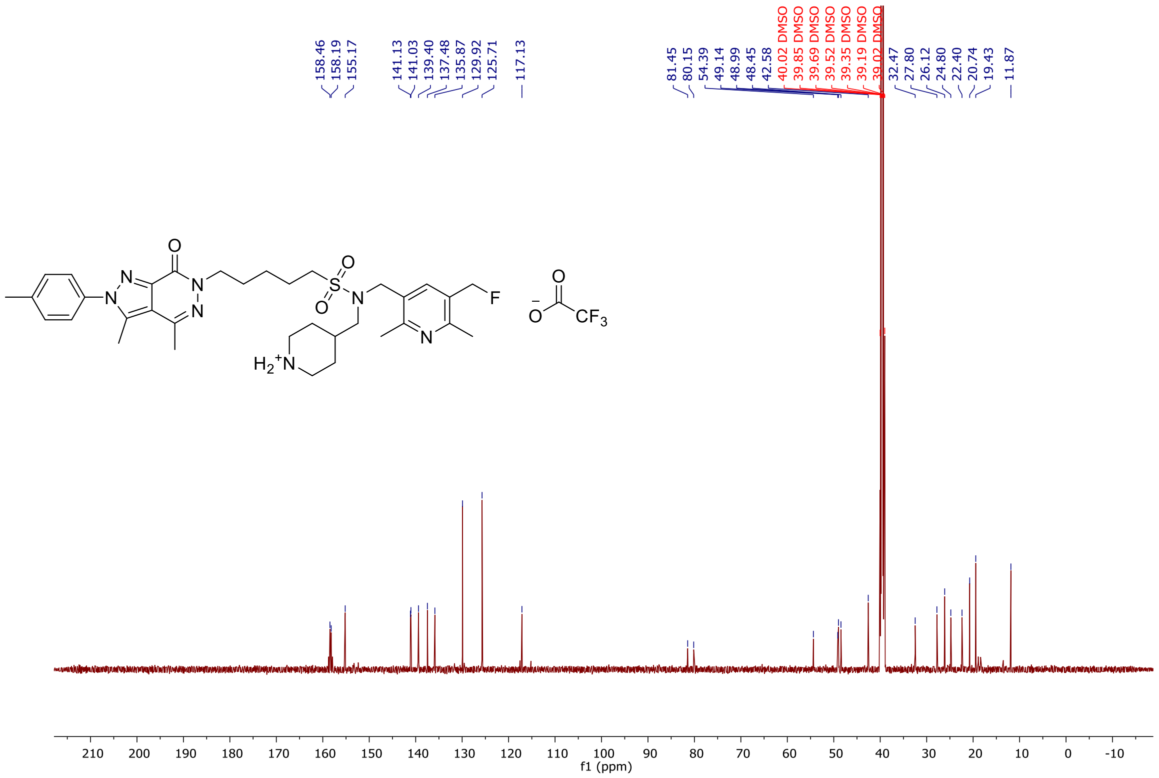

## $^{1}$H NMR Spectrum of **14b** (600 MHz, DMSO-$d_6$)

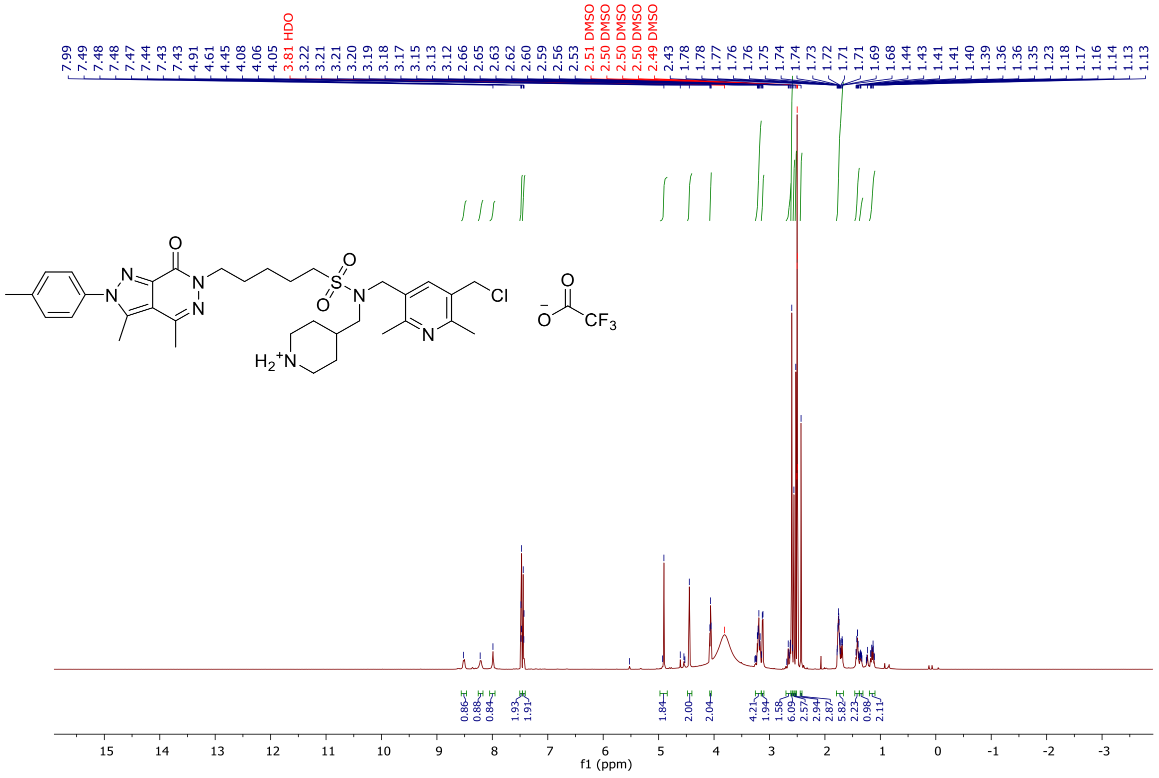


## $^{13}$C NMR Spectrum of **14b** (151 MHz, DMSO-$d_6$)

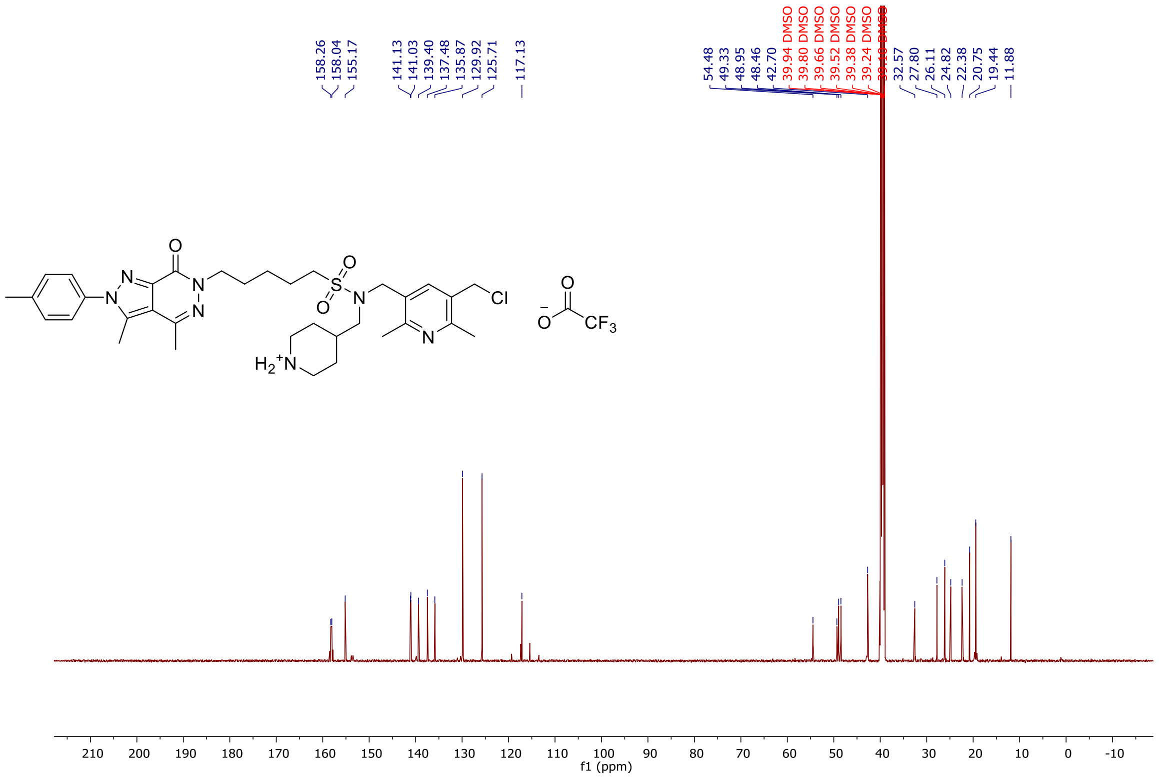

**$^{1}$H NMR** Spectrum of **15a** (500 MHz, DMSO-$d_6$)

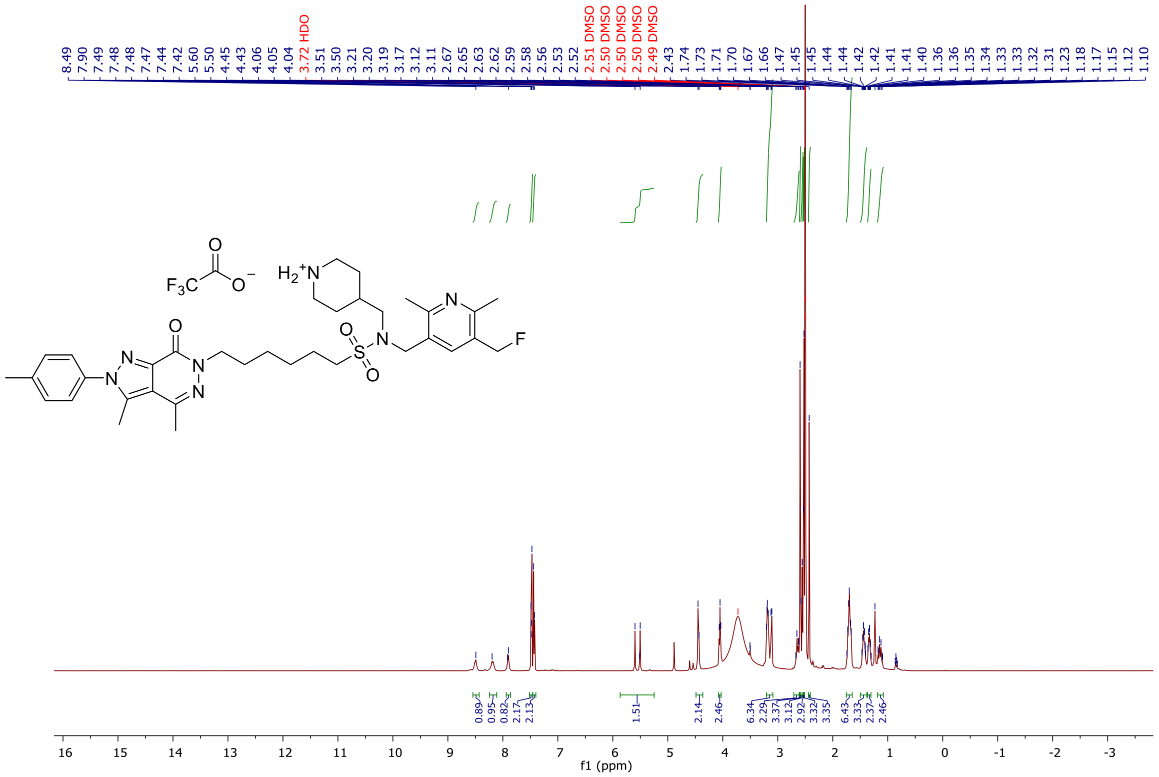

**$^{13}$C NMR** Spectrum of **15a** (126 MHz, DMSO-$d_6$)

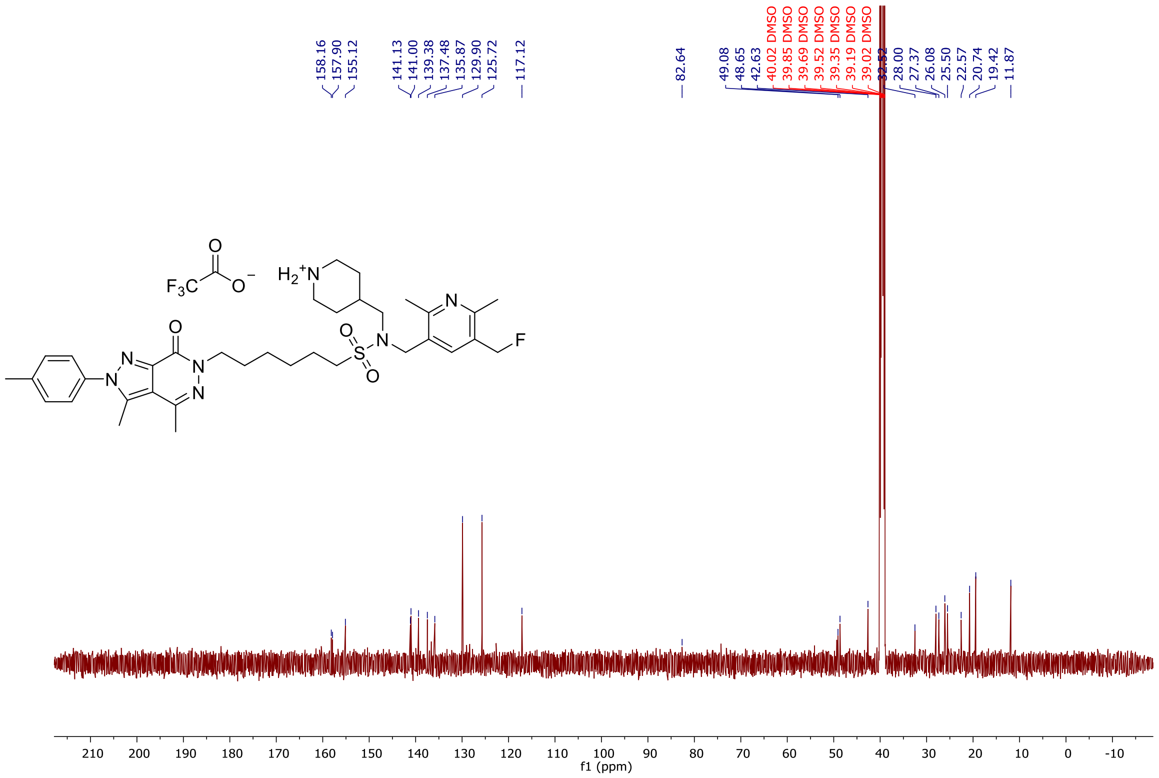

$^{1}$**H NMR** Spectrum of **15b** (600 MHz, DMSO-$d_6$)

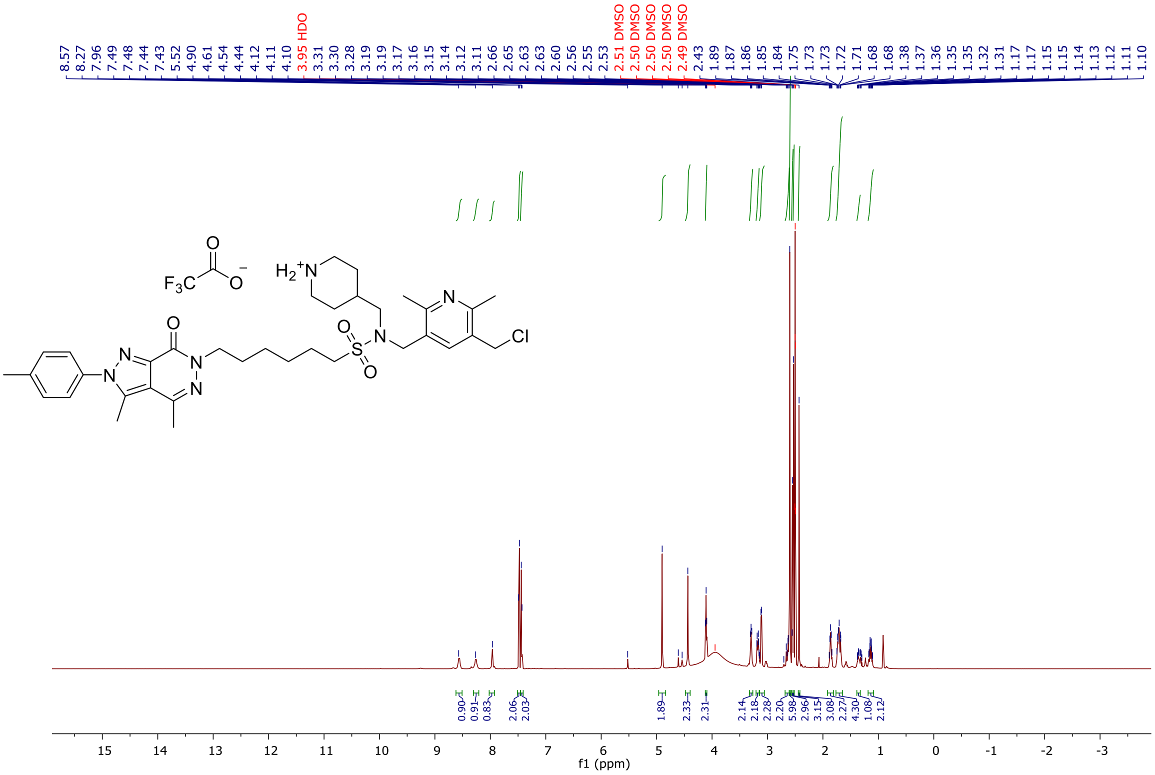

$^{13}$**C NMR** Spectrum of **15b** (151 MHz, DMSO-$d_6$)

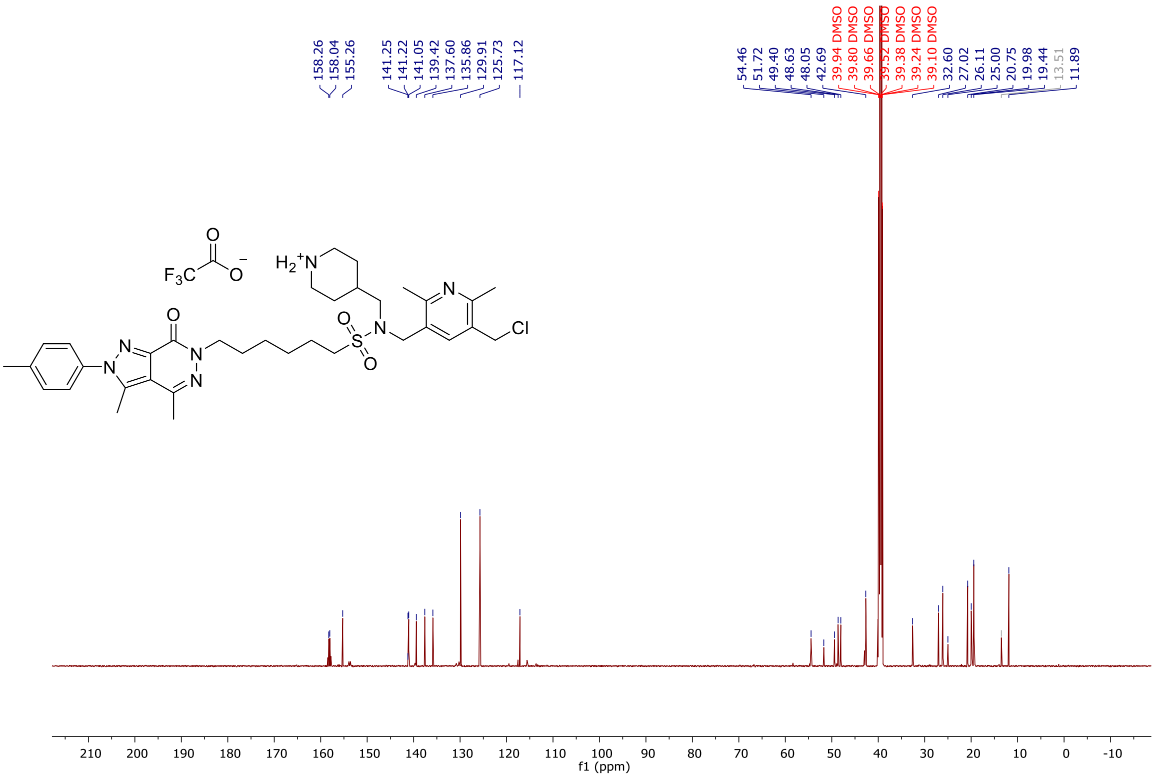

**$^{1}$H NMR** Spectrum of **22a** (600 MHz, DMSO-$d_6$)

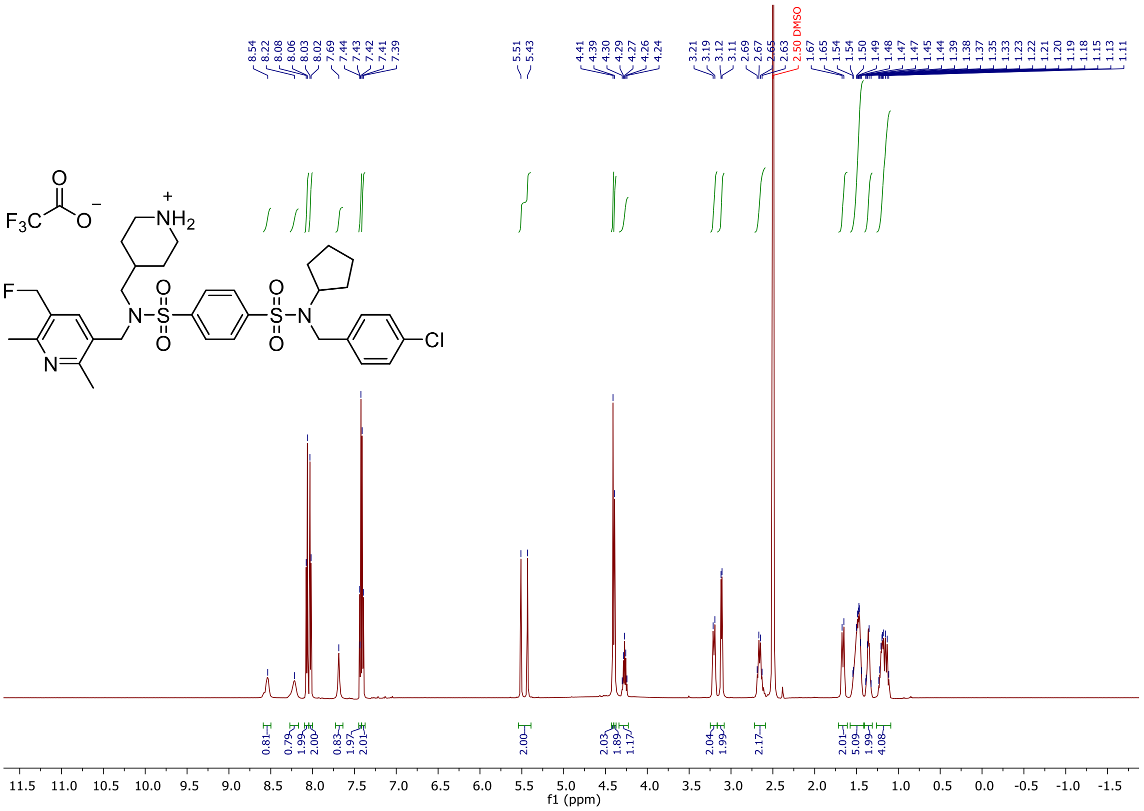


**$^{13}$C NMR** Spectrum of **22a** (176 MHz, DMSO-$d_6$)

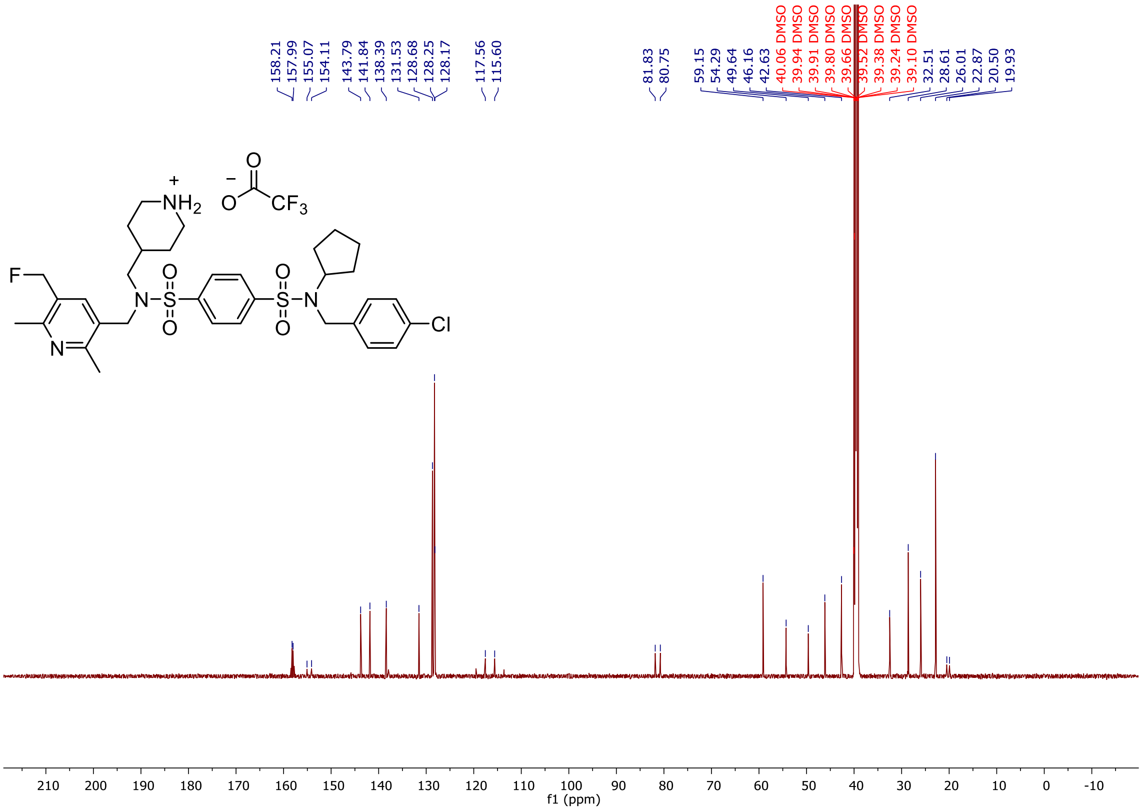

**$^{19}$F NMR** Spectrum of **22a** (176 MHz, DMSO-$d_6$)

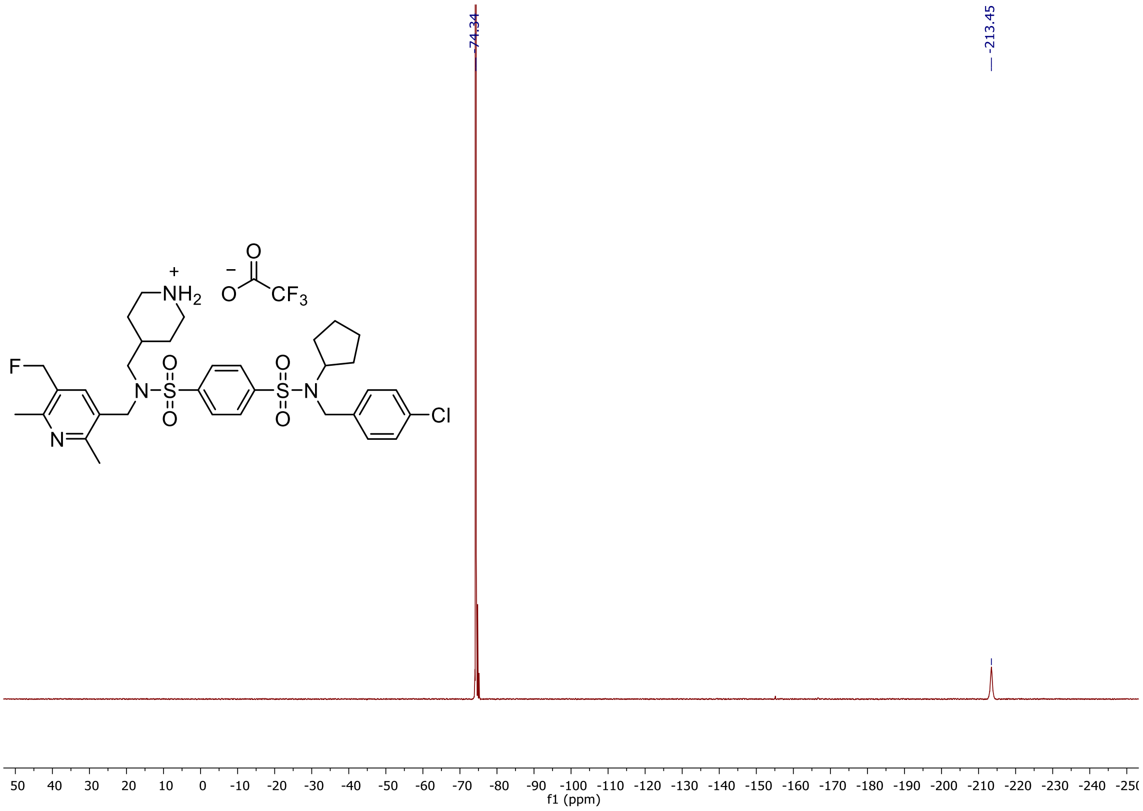


## HPLC purity

5%-65% Acetonitrile/$H_2O$, 0.1% TFA

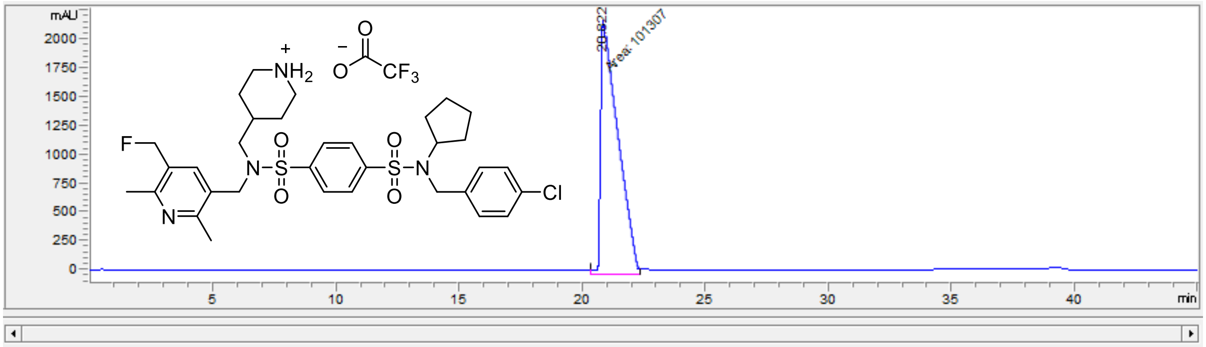


| File Information | |
|---|---|
| LC-File | ST1425_PROD.D |

| # | Time | Area | Height | Width | Area% | Symmetry |
|---|---|---|---|---|---|---|
| 1 | 20.822 | 101307.1 | 2202.8 | 0.7665 | 100.000 | 0.165 |

**$^{1}$H NMR** Spectrum of **22b** (500 MHz, DMSO-$d_6$)

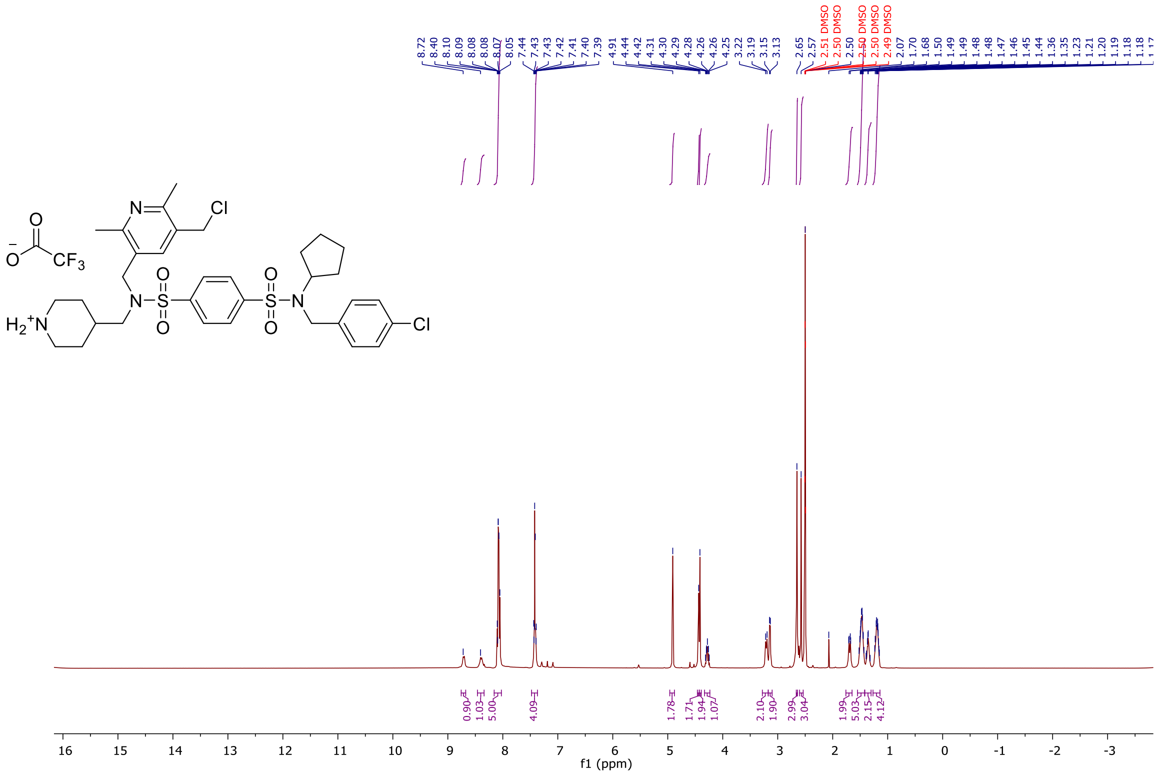


**$^{13}$C NMR** Spectrum of **22b** (126 MHz, DMSO-$d_6$)

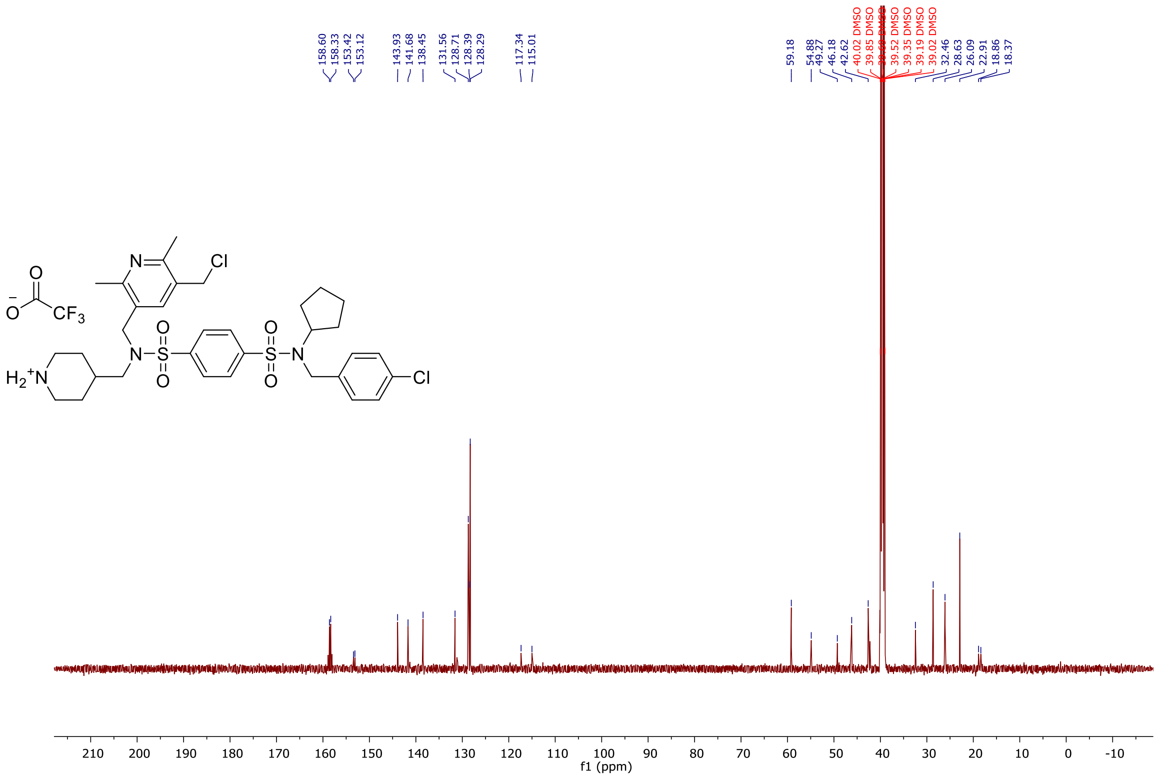

## HPLC traces for the Representative Compounds.

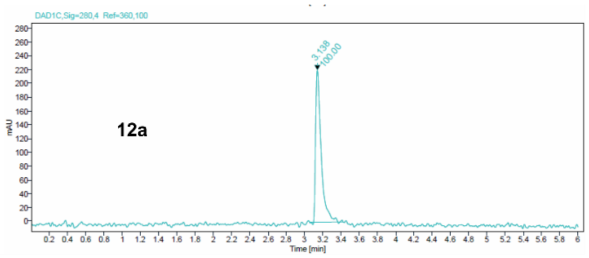


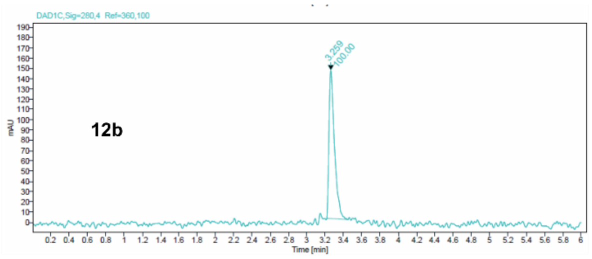


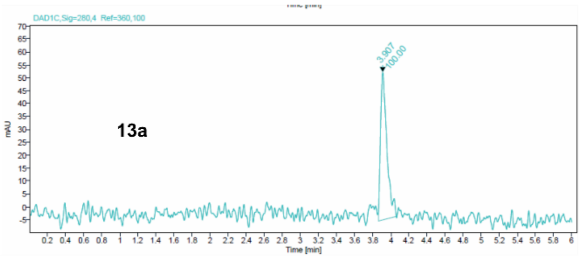


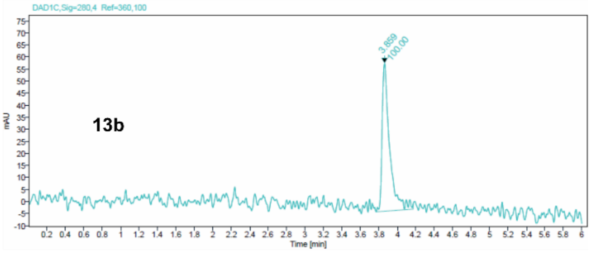


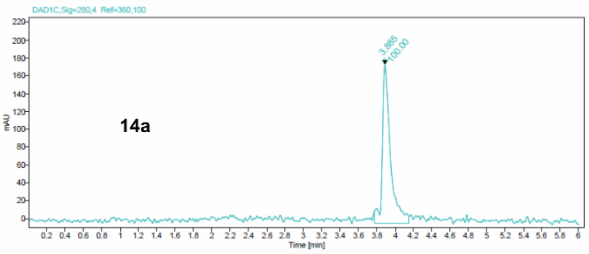


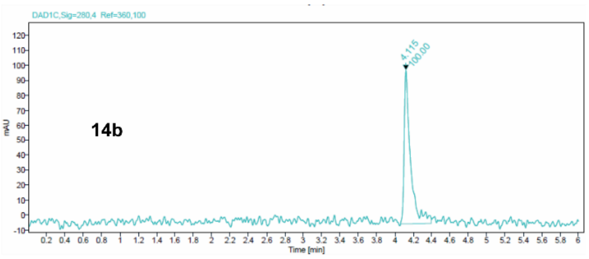


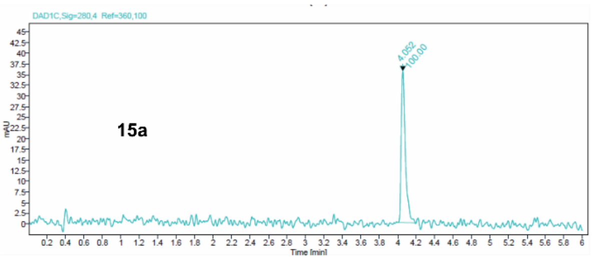


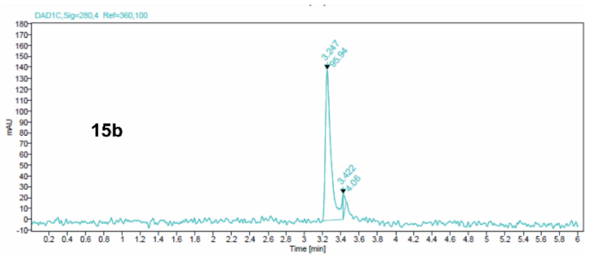


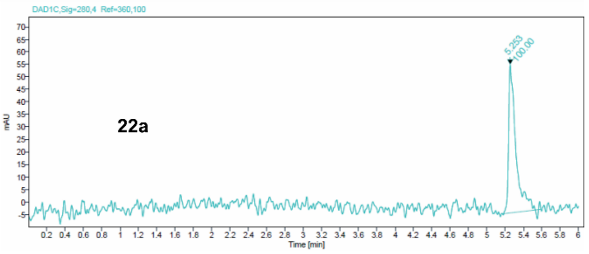


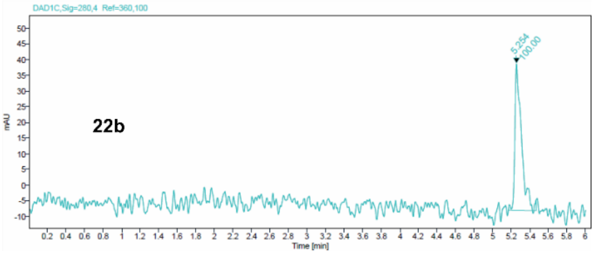


10%-100% Acetonitrile/$H_2O$, 0.1% TFA, C18 column, 6 min flow, ambient temperature.

## 4. Material List

| REAGENT or RESOURCE | SOURCE | IDENTIFIER |
|---|---|---|
| **Chemicals, Peptides, and Recombinant Proteins** | | |
| Dulbecco's Modified Eagle Medium + GlutaMAX | ThermoFisher Scientific | 2902879 |
| 2-Hydroxypropyl-ß-cyclodextrin | Carbolution | CC20035 |
| DMEM medium | PAN Biotech | P04-03550 |
| Non-essential amino acids (NEAA) | PAN Biotech | P08-32100 |
| Sodium pyruvate | PAN Biotech | P04-43100 |
| RMPI-1640 medium | PAN Biotech | P04-18047 |
| MyCoy's 5A medium | PAN Biotech | P04-05500 |
| MEM Eagle | PAN Biotech | P04-08500 |
| FBS | Gibco | #10270-106 |
| Anti-PDEδ antibody | Invitrogen | PA5-22008, RRID: AB_11154288 |
| Anti-S6 Ribosomal Protein (54D2) | Cell Signaling | 2317 |
| Anti-phospho-S6 (S235/236) | Cell Signaling | 4856 |
| Anti-p44/42 Erk1/2 (3A7) | Cell Signaling | 9107 |
| Anti-phospho-p44/42 ERK1/2 (Thr202/Tyr204) | Cell Signaling | 4370 |
| IRDye 800CW-conjugated secondary antibody | LI-COR Biosciences | #926-32210, RRID: AB_621842 |
| Glu-C | Promega | V1651 |
| Trypsin/Lys-C mix | Promega | V5072 |
| Isopropyl β-D-1-thiogalactopyranoside (IPTG) | Sigma-Aldrich | CAS 367-93-1 |
| Tris-HCl | Carl Roth | CAS 1185-53-1 |
| NaCl | VWR | CAS 7647-14-5 |
| β-mercaptoethanol | Sigma-Aldrich | CAS 60-24-2 |
| Phenylmethylsulfonyl fluoride | ThermoFisher Scientific | CAS 329-98-6 |
| Dithiothreitol | Gerbu Biotechnik | CAS 3483-12-3 |
| Imidazole | Sigma-Aldrich | CAS 288-32-4 |
| Sinapinic acid | Sigma-Aldrich | CAS 530-59-6 |
| HEPES | Carl Roth | CAS 7365-45-9 |
| TFA | Sigma Aldrich | CAS 76-05-1 |
| Sodium acetate | VWR | CAS 127-09-3 |
| Sodium citrate | VWR | CAS 6132-04-3 |
| Potassium phosphate | TCI | CAS 7778-77-0 |
| Glutathione | Sigma Aldrich | CAS 70-18-8 |
| EDTA disodium salt dihydrate | TCI | CAS 6381-92-6 |
| Guanidine hydrochloride | Carl Roth | CAS 50-01-1 |
| 2-Chloroacetamide | Sigma Aldrich | CAS 79-07-2 |
| Ammonium bicarbonate | Sigma Aldrich | CAS 1066-33-7 |
| Acetonitrile HPLC grade | Sigma Aldrich | CAS 75-05-8 |
| Triethylammonium bicarbonate | Sigma Aldrich | CAS 15715-58-9 |

| | | |
|---|---|---|
| 2,2,2-Trifluoroethanol | ThermoFisher Scientific | CAS 75-89-8 |
| Sera-Mag™ SpeedBead™ carboxylate modified magnetic particles | GE Healthcare | #45152105050250 and #65152105050250 |
| MagReSyn $TiO_2$ particles | ReSyn Biosciences | MR-TID005 |
| Glycolic acid | VWR | CAS 79-14-1 |
| 4-Bromobenzenesulfonyl chloride | TCI | CAS 98-58-8 |
| 4-Chloro-N-cyclopentylbenzenemethanamine | Sigma Aldrich | CAS 66063-15-8 |
| Triethylamine | Sigma Aldrich | CAS 121-44-8 |
| Dichloromethane | VWR | CAS 75-09-2 |
| Benzyl mercaptan | TCI | CAS 100-53-8 |
| 1,3-Dichloro-5,5-dimethylhydantoin | TCI | CAS 118-52-5 |
| 4-Aminomethylpiperidine-1-carboxylic acid tert-butyl ester | TCI | CAS 144222-22-0 |
| Ethyl acetate | VWR | CAS 141-78-6 |
| Diethyl 2,6-dimethylpyridine-3,5-dicarboxylate | TCI | CAS 1149-24-2 |
| Lithium aluminum hydride, 1M in THF | Sigma Aldrich | CAS 16853-85-3 |
| Dry tetrahydrofuran | Sigma Aldrich | CAS 109-99-9 |
| **Critical Commercial Assays** | | |
| MycoAlert™ Mycoplasma Detection Kit | Lonza | #LT07-318 |
| Pierce™ 660 nm protein assay reagents | Thermo Scientific | #22662 |
| **Experimental Models: Cell Lines** | | |
| Jurkat | DSMZ GmbH | ACC282, RRID: CVCL_0065 |
| PA-TU-8902 | DSMZ GmbH | ACC179, RRID: CVCL_1845 |
| BxPC3 | DSMZ GmbH | ACC760, RRID: CVCL_0186 |
| HCT116 | DSMZ GmbH | ACC581, RRID: CVCL_0291 |
| LS-174T | DSMZ GmbH | ACC759, RRID: CVCL_1384 |
| A549 | DSMZ GmbH | ACC107, RRID: CVCL_0023 |
| MIA PaCa-2 | ATCC (USA) | CRM-CRL-1420, RRID: CVCL_0428 |
| PANC-1 | ATCC (USA) | ATCC-CRL-1469, RRID: CVCL_0480 |
| SW480 | ATCC (USA) | CCL-228, RRID: CVCL_0546 |
| HT-29 | ATCC (USA) | ATCC-HTB-38, RRID: CVCL_0320 |
| HEK293T | ATCC (USA) | ATCC-CRL-3216, RRID: CVCL_0063 |
| NCI-H441 | ATCC (USA) | ATCC-CRM-HTB-174, RRID: CVCL_1561 |
| NCI-H358 | LGC Standards (Germany) | ATCC-CRL-5807, RRID: CVCL_1559 |
| U2OS | CLS Cell Lines Service GmbH | CLS-300364, RRID: CVCL_0042 |
| SK-LU-1 | CLS Cell Lines Service GmbH | CLS-300335, RRID: CVCL_0629 |
| HAP1 wild type | Horizon Discovery | Horizon #C631, RRID: CVCL_Y019 |
| HAP1 PDE6D knockout | Horizon Discovery | Horizon #HZGHC006484c003, RRID: CVCL_XR47 |

| | | |
|---|---|---|
| Hs 578T | NIH/NCI-DTP | RRID: CVCL_0332 |
| **Experimental Models: Mouse Lines** | | |
| *K-Ras*$^{LSL.G12D/wt}$ ; *Trp53*$^{fl/fl}$ | DuPage et al., 2009 | C57BL/6J |
| **Software and Algorithms** | | |
| Adobe Illustrator | Adobe | |
| Inkscape | Inkscape | |
| DataViewer v2.1 | Extron | |
| Microsoft Office 365 | Microsoft | |
| NRecon | Bruker | |
| PRISM | Graphpad | |
| PyRat | Scionics | |
| MaxQuant (v.2.2.0.0) | MaxQuant | |
| Image Lab (v6.0) | Bio-rad | |
| IncuCyte S3 system software | Sartorius | |
| MestReNova x64 | Mestrelab | |
| X-ray Detector Software (XDS) | MPI for Medical Research | |
| XSCALE | MPI for Medical Research | |
| Phaser (Phenix suite) | Phenix | |
| phenix.refine | Phenix | |
| CCP4 program suite with WinCOOT | GNU GPL | |
| PyMOL Molecular Graphics System | Schrödinger, LLC | |
| LigPlot Plus | EMBL-EMI | |

## 5. References


1. Martín-Gago, P.; Fansa, E. K.; Winzker, M.; Murarka, S.; Janning, P.; Schultz-Fademrecht, C.; Baumann, M.; Wittinghofer, A.; Waldmann, H., Covalent Protein Labeling at Glutamic Acids. *Cell Chem. Biol.* **2017,** *24* (5), 589-597.e5.
2. Papke, B.; Murarka, S.; Vogel, H. A.; Martín-Gago, P.; Kovacevic, M.; Truxius, D. C.; Fansa, E. K.; Ismail, S.; Zimmermann, G.; Heinelt, K.; Schultz-Fademrecht, C.; Al Saabi, A.; Baumann, M.; Nussbaumer, P.; Wittinghofer, A.; Waldmann, H.; Bastiaens, P. I. H., Identification of pyrazolopyridazinones as PDEδ inhibitors. *Nat. Commun.* **2016,** *7* (1), 11360.
3. Martín-Gago, P.; Fansa, E. K.; Klein, C. H.; Murarka, S.; Janning, P.; Schürmann, M.; Metz , M.; Ismail, S.; Schultz-Fademrecht, C.; Baumann, M.; Bastiaens, P. I. H.; Wittinghofer, A.; Waldmann, H., A PDE6δ-KRas Inhibitor Chemotype with up to Seven H-Bonds and Picomolar Affinity that Prevents Efficient Inhibitor Release by Arl2. *Angew. Chem., Int. Ed. Engl.* **2017,** *56* (9), 2423-2428.
4. Zimmermann, G.; Papke, B.; Ismail, S.; Vartak, N.; Chandra, A.; Hoffmann, M.; Hahn, S. A.; Triola, G.; Wittinghofer, A.; Bastiaens, P. I. H.; Waldmann, H., Small molecule inhibition of the KRAS–PDEδ interaction impairs oncogenic KRAS signalling. *Nature* **2013,** *497* (7451), 638-642.
5. Waldmann, H. Z., R.; liu, J.; Gasper, R.; Janning, P. , Covalent Modification of Glutamic Acid Inspired by HaloTag Technology. *ChemRxiv. This content is a preprint and has not been peer-reviewed.* **2025**.
6. Milacic, M.; Beavers, D.; Conley, P.; Gong, C.; Gillespie, M.; Griss, J.; Haw, R.; Jassal, B.; Matthews, L.; May, B.; Petryszak, R.; Ragueneau, E.; Rothfels, K.; Sevilla, C.; Shamovsky, V.; Stephan, R.; Tiwari, K.; Varusai, T.; Weiser, J.; Wright, A.; Wu, G.; Stein, L.; Hermjakob, H.; D'Eustachio, P., The Reactome Pathway Knowledgebase 2024. *Nucleic Acids Res.* **2023,** *52* (D1), D672-D678.
7. Casado, P.; Rodriguez-Prados, J.-C.; Cosulich, S. C.; Guichard, S.; Vanhaesebroeck, B.; Joel, S.; Cutillas, P. R., Kinase-Substrate Enrichment Analysis Provides Insights into the Heterogeneity of Signaling Pathway Activation in Leukemia Cells. *Sci. Signaling* **2013,** *6* (268), rs6-rs6.
8. Horn, H.; Schoof, E. M.; Kim, J.; Robin, X.; Miller, M. L.; Diella, F.; Palma, A.; Cesareni, G.; Jensen, L. J.; Linding, R., KinomeXplorer: an integrated platform for kinome biology studies. *Nat. Methods* **2014,** *11* (6), 603-604.
9. Hornbeck, P. V.; Zhang, B.; Murray, B.; Kornhauser, J. M.; Latham, V.; Skrzypek, E., PhosphoSitePlus, 2014: mutations, PTMs and recalibrations. *Nucleic Acids Res.* **2015,** *43* (D1), D512-D520.
10. Ziegler, S.; Sievers, S.; Waldmann, H., Morphological profiling of small molecules. *Cell Chem. Biol.* **2021,** *28* (3), 300-319.
11. Pahl, A.; Schölermann, B.; Lampe, P.; Rusch, M.; Dow, M.; Hedberg, C.; Nelson, A.; Sievers, S.; Waldmann, H.; Ziegler, S., Morphological subprofile analysis for bioactivity annotation of small molecules. *Cell Chem. Biol.* **2023,** *30* (7), 839-853.e7.
12. Tandon, A.; Santura, A.; Waldmann, H.; Pahl, A.; Czodrowski, P., Identification of lysosomotropism using explainable machine learning and morphological profiling cell painting data. *RSC Med. Chem.* **2024,** *15* (8), 2677-2691.
13. Cox, J.; Mann, M., MaxQuant enables high peptide identification rates, individualized p.p.b.-range mass accuracies and proteome-wide protein quantification. *Nat. Biotechnol.* **2008,** *26* (12), 1367-1372.
14. Humphrey, S. J.; Karayel, O.; James, D. E.; Mann, M., High-throughput and high-sensitivity phosphoproteomics with the EasyPhos platform. *Nat. Protoc.* **2018,** *13* (9), 1897-1916.
15. Zhang, H.; Steele, J. R.; Kahrood, H. V.; Lucas, D. D.; Shah, A. D.; Schittenhelm, R. B., Phospho-Analyst: An Interactive, Easy-to-Use Web Platform To Analyze Quantitative Phosphoproteomics Data. *J. Proteome Res.* **2023,** *22* (9), 2890-2899.

16. DuPage, M.; Dooley, A. L.; Jacks, T., Conditional mouse lung cancer models using adenoviral or lentiviral delivery of Cre recombinase. *Nat. Protoc.* **2009,** *4* (7), 1064-1072.
